# Probiotics are beneficial for liver cirrhosis: a systematic review and meta-analysis of randomized control trials

**DOI:** 10.3389/fmed.2024.1379333

**Published:** 2024-03-28

**Authors:** Xing Yang, Langhuan Lei, Wei Shi, Xiaozhen Li, Xiaozhi Huang, Liuyan Lan, Jiali Lin, Qiuyu Liang, Wei Li, Jianrong Yang

**Affiliations:** ^1^Health Management Research Institute, People’s Hospital of Guangxi Zhuang Autonomous Region and Guangxi Academy of Medical Sciences, Nanning, China; ^2^Health Management Center, People's Hospital of Guangxi Zhuang Autonomous Region and Guangxi Academy of Medical Sciences, Nanning, China; ^3^Office of Hospital Quality and Safety Management Committee, People's Hospital of Guangxi Zhuang Autonomous Region and Guangxi Academy of Medical Sciences, Nanning, China

**Keywords:** probiotics, liver cirrhosis, hepatic encephalopathy, liver function, meta-analysis

## Abstract

**Introduction:**

Gut dysbiosis may play a pivotal role in the pathogenesis of cirrhosis and the severity of complications. Numerous studies have investigated the probiotics as treatments for cirrhosis. However, there is still a lack of definitive evidence confirming the beneficial effects of probiotics on cirrhosis.

**Methods:**

Databases including PubMed, Embase, Web of Science, and the Cochrane Library were systematically searched for randomized controlled trials that compared the effects of probiotic intervention and control treatments, including placebo, no treatment, and active control, on cirrhosis, published from inception to February 2024. Outcomes included hepatic encephalopathy (HE) reversal, safety and tolerability of probiotics, liver function, quality of life, and other cirrhotic-related outcomes. A meta-analysis was conducted to synthesize evidence.

**Results:**

Thirty studies were included. The quantitative synthesis results showed that compared with the control group, probiotics significantly reverse minimal hepatic encephalopathy (MHE) (risk ratio [RR] 1.54, 95% confidence interval [CI] 1.03 to 2.32) and improve HE (RR 1.94, 95% CI 1.24 to 3.06). Additionally, probiotics demonstrated higher safety and tolerability by causing a lower incidence of serious adverse events (RR 0.71, 95% CI 0.58 to 0.87). Probiotics could potentially improve liver function by reducing the Model for End-Stage Liver Disease (MELD) scores (standardized mean difference [SMD] -0.57, 95% CI -0.85 to −0.30), and displayed favorable changes in quality of life (SMD 0.51, 95% CI 0.27 to 0.75) and gut flora (SMD 1.67, 95% CI 1.28 to 2.06).

**Conclusion:**

This systematic review and meta-analysis offers compelling evidence that probiotics are beneficial for cirrhosis by demonstrating reversal of HE, potential for liver function improvements, enhancements in quality of life, and regulation of gut dysbiosis. Furthermore, the apparent safety profile suggests that probiotics are a promising intervention for treating cirrhosis.

**Clinical trial registration number:**

CRD42023478380.

## 1 Introduction

Liver cirrhosis is the end stage of chronic liver disease, commonly caused by viral hepatitis, nonalcoholic steatohepatitis, and alcohol (1). Cirrhosis is within the top 20 causes of disability-adjusted life years and years of life lost, accounting for 1.6 and 2.1% of the global burden (2). Being a major cause of morbidity and mortality among individuals with chronic liver disease worldwide, cirrhosis affects over 160 million people and results in more than 1.3 million deaths each year (3–5). As currently one of the top 10 leading causes of death globally, cirrhosis imposes a great health burden in many countries (6). The burden has escalated at the worldwide level since 1990, partly because of population growth and aging (5). Thus, it is meaningful to explore effective treatments for reversing cirrhosis and preventing severe liver function and even systemic damage.

It has been proven that the occurrence and progression of cirrhosis are directly or indirectly associated with local and systemic immune and inflammatory changes (7). The gut microbiota can contribute to systemic inflammation (8). Changes in the gut microbiota are related to immune homeostasis disturbances (9). Therefore, studies have indicated that gut dysbiosis may play a role in the pathogenesis of cirrhosis, contributing to the severity of complications such as hepatic encephalopathy (HE), hepatocellular carcinoma, and the progression of acute-on-chronic liver failure (10, 11). Recognizing the association between gut imbalance and liver cirrhosis, an increasing number of studies have focused on the use of probiotics among patients with cirrhosis.

Probiotics are presently defined as “live microorganisms that, when administered in adequate amounts, confer a health benefit on the host” (12). In the context of aging, evidence shows that probiotics are valuable modulators of age-related pathologies and morbidity (13). Emerging studies are exploring various probiotic supplements for the treatment of cirrhosis. Some studies have suggested the effectiveness of probiotic therapy in cirrhotic patients. For example, one study employed *Lactobacillus* to prevent cirrhosis, and the results indicated an improvement in dysbiosis (14). Another clinical trial demonstrated that *Bifidobacterium* can promote the transformation of macrophages and control the inflammatory response among cirrhotic patients (15). However, some other studies did not demonstrate a significant protective effect of probiotic supplementation on cirrhosis (16, 17). These conflicting results may be partially due to the small size of cohorts or the biased design of individual trials, which could be solved by a meta-analysis. Although there were meta-analyses exploring the effect of probiotics on cirrhosis, most of the studies focused on patients during the progressive period of minimal hepatic encephalopathy (MHE) or HE (18, 19). Early detection and timely treatment of cirrhosis are essential to improving the outcomes of cirrhotic patients. Moreover, there is a study (20) that did not exclusively focus on randomized controlled trials (RCTs) to conduct a comprehensive analysis, preventing it from reaching the pinnacle of the evidence pyramid. Clear evidence is urgently needed to determine whether probiotics have beneficial effects on cirrhosis during any progressive period.

Thus, this systematic review and meta-analysis were conducted based on RCTs to assess the comparative outcomes of cirrhosis, including HE reversal, liver function, gut microbial taxonomy, and mortality, between probiotic and control treatments using quantitative statistical methods. A definitive conclusion on the therapeutic effects of probiotics will be derived to provide evidence for the efficacy of probiotics among cirrhotic patients.

## 2 Materials and methods

This systematic review and meta-analysis was conducted based on the Preferred Reporting Items for Systematic Reviews and Meta-Analyses guidelines (21).

### 2.1 Search strategy

PubMed, Embase, Web of Science, and the Cochrane Library were systematically searched for RCTs comparing the effects of probiotic intervention with control treatments in patients with cirrhosis, published in English from inception to February 2024. A search strategy was developed based on keywords, medical subject headings (MeSH) terms, and synonyms (Supplementary Table S1). In addition to the database searches, we meticulously reviewed the reference lists of reviews, original studies, and related systematic reviews to identify additional studies potentially eligible for inclusion that could have been overlooked in our initial search.

### 2.2 Selection criteria

To meet the inclusion criteria, studies had to: (1) be conducted among patients aged more than 18 years, with any type of liver cirrhosis irrespective of etiology, during any disease-progressive period; (2) be RCTs that compared any probiotic intervention at any dose for any duration in a treatment group against a control group receiving placebo, no treatment, or active control treatment, including lactulose (22), rifaximin (23), placebo, standard treatment, or no treatment; and (3) report clinical outcomes related to cirrhosis such as ammonia levels, adverse events after receiving probiotics, liver functions, and mortality. In the study conducting co-interventions of probiotics and prebiotics or medication, equal doses of prebiotics or medication had to be administered in the control groups to ensure exploring the effect of probiotic intervention alone.

The exclusion criteria were as follows: (1) animal experiments or *in vitro* studies; (2) reviews, meta-analysis, comment, letter, poster abstract, editorial, case report, and correction; (3) papers could not be downloaded from databases; (4) a lack of data information available for synthesis analysis.

### 2.3 Study selection and data extraction

The titles and abstracts identified through the database searches were exported to EndNote X9, and duplicates were removed. The review process was carried out according to the guidelines laid out in the QUOROM statement (24). Two investigators (XY and LL) independently reviewed the titles and abstracts of all identified studies that were eligible for inclusion. Then, a full-text review of the potential papers was conducted to determine the final included studies. Another reviewers (WS and XL) were available for the final determination of whether a publication should be included if there were discrepancies. Data extraction of the included studies was done using a pre-designed standardized Excel form that included the following information: author, country, study period, target population, interventions of treatment or control group, sample size, treatment period, study duration, and main findings.

### 2.4 Quality assessment

The quality of individual trials was assessed using the Cochrane Risk of Bias instrument (25), evaluating seven key domains: random sequence generation, allocation concealment, blinding of participants and personnel, blinding of outcome assessment, incomplete outcome data, selective reporting, and other bias. Two reviewers (XY and LL) independently worked on the quality assessment. If there were any disagreements between reviewers, another two reviewers (WS and XL) were found to arbitrate.

### 2.5 Assessment of heterogeneity and publication bias

Both the *Chi*^2^ test and the Higgins *I*^2^ test were applied to assess the heterogeneity of studies in meta-analysis. When the *p*-value of the *Chi*^2^ test was less than 0.05 and I^2^ exceeded 50%, a random-effects model was selected, while a fixed model would be chosen if the results showed a *p*-value more than 0.05 and *I*^2^ below 50%. Sensitivity analysis would be conducted if the model was unstable. Publication bias was assessed by funnel plot analysis. Egger’s test was used for continuous outcomes, while Peter’s test was conducted among dichotomous outcomes. A *p*-value >0.05 means no evidence of publication bias; otherwise, there would be a publication bias.

### 2.6 Outcome measures

Two researchers (XY and LL) coded the outcome measurements related to the effect of probiotics on cirrhosis for each included study separately. If there existed discrepancies, the two coding results were compared and discussed with another two researchers (WS and XL). Outcomes were finally categorized into the following seven main aspects: HE reversal, safety and tolerability of probiotics, liver function measurements, quality of life, effect on gut flora, inflammatory cytokines changes, and mortality.

### 2.7 Statistical analysis

Risk ratio (RR) and corresponding 95% confidence interval (CI) were used to assess the differences between probiotics and control groups when outcomes were dichotomous, while standardized mean difference (SMD) and 95% CI were used to evaluate the differences for continuous outcomes. If the outcomes were measured at a different time point, the terminal follow-up visits were chosen to be analyzed. Additionally, we conducted subgroup analyses for different types of probiotics and different intervention durations. A fixed-effects or random-effects model was selected according to the deviance information criterion (DIC). All tests were two-sided, with a *p*-value of 0.05 set as the threshold for significance. Through R 4.3.1, a forest plot was built for each outcome in the R package meta,^1^ and the result of the risk of bias was visualized by the R package robvis.^2^

## 3 Results

### 3.1 Literature selection

**Figure 1:**
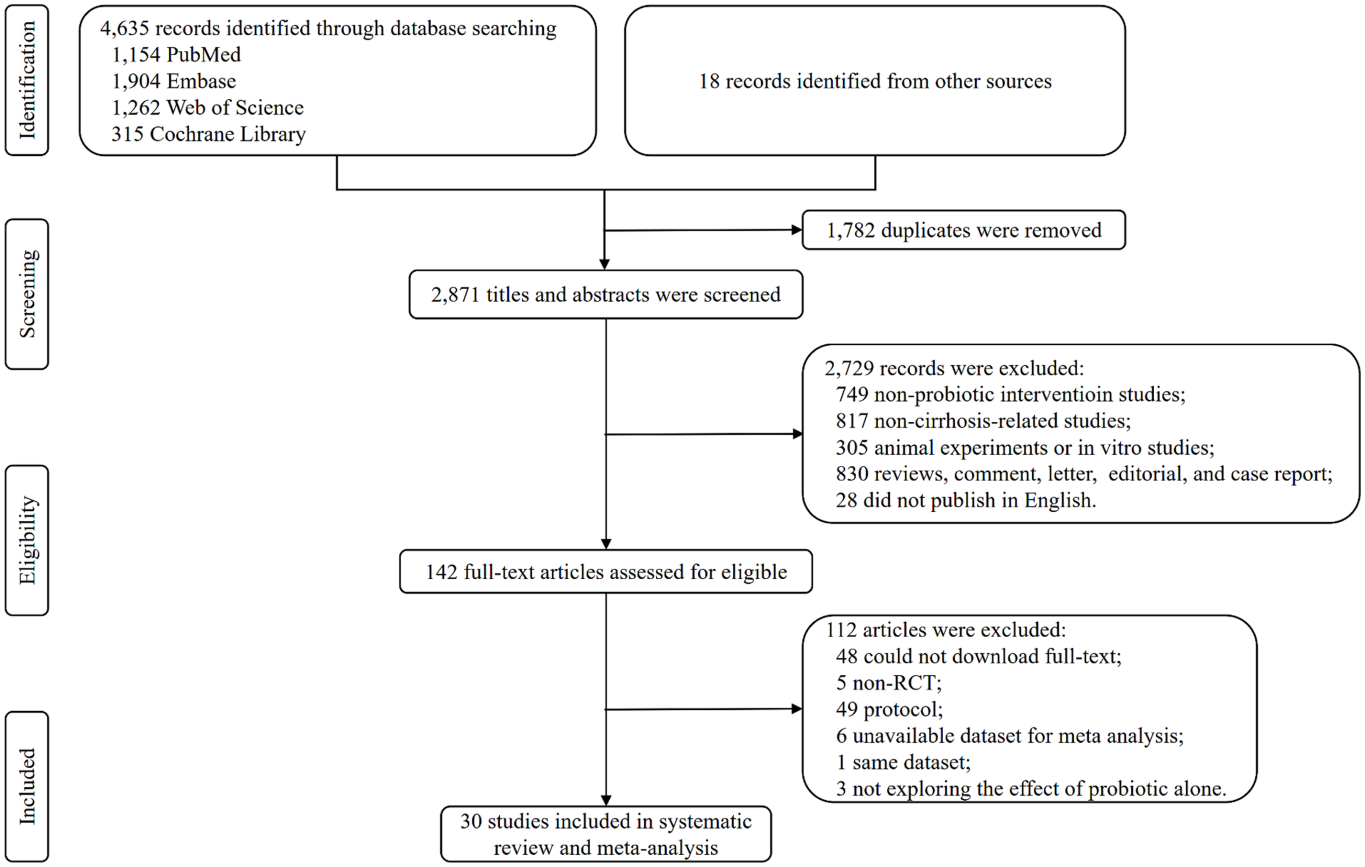
Flow chart of the study selection procedure.

Database searching identified 4,635 records, and 18 records were identified from other sources. There were 2,871 titles and abstracts screening after duplicates were removed. Then, 2,729 irrelevant and ineligible records were excluded, and 142 articles were used to conduct a full-text screening. After further excluding 112 articles for various reasons, a total of 30 RCTs were finally included in the systematic review and meta-analysis (Figure 1).

### 3.2 Study characteristics

Among the 30 trials that compared the therapeutic effect of probiotic treatment with control treatment in liver cirrhosis, 17 compared probiotics with placebo (17, 26–41), 7 compared probiotics with lactulose (42–47) or fermentable fiber (48), 3 compared probiotics with standard treatment (49–51), and 2 compared probiotics with no treatment (52, 53). And the control treatments in the remaining trial were lactulose and rifaximin (54). These studies were from 14 countries and contained a total of 2,084 cirrhotic patients, including 1,049 in the probiotic group and 1,035 in the control group (Table 1). Different subtypes and dosages of probiotics were used in different trials (Supplementary Table S2). More comprehensive details of the included studies are presented in Supplementary Tables S2–S3.

**Table 1:** Study characteristics of the included studies.

Author, Year [Ref]	Country	Study period	Target population	Interventions of treatment group (sample size)	Treatment duration	Study duration	Interventions of control group (sample size)	Main findings
Agrawal A, 2012	India	2008.10–2009.12	Consecutive cirrhotic patients recovered from HE	Probiotics: Lactobacillus, Bifidobacterium, and *Streptococcus sali*var*ius* subsp. thermophilus (*n* = 77)	3 months	12 months	No treatment (*n* = 78)	HE reversal, safety and tolerability, mortality.
Bajaj J S, 2008	USA	2005.10–2007.1	Nonalcoholic MHE cirrhotics	Probiotic yogurt: *S. thermophilus*, *L. bulgaricus*, *L. acidophilus*, Bidobacteria, and *L. casei* (n = 17)	2 months	2 months	No treatment (*n* = 8)	HE reversal, safety and tolerability, liver function, quality of life, inflammatory cytokines change.
Bajaj J S, 2014	USA	NA	Cirrhosis with MHE	Probiotic: Lactobacillus GG (LGG) (*n* = 14)	2 months	2 months	Placebo (*n* = 16)	Safety and tolerability, inflammatory cytokines change.
Dhiman R K, 2014	India	2010.1–2012.9	Cirrhosis recovered from HE	Probiotic: VSL#3 (*n* = 66)	6 months	6 months	Placebo (*n* = 64)	Safety and tolerability, mortality.
Efremova I, 2024	Russia	NA	Cirrhosis	Probiotic: S. boulardii CNCM I-745 (*n* = 20)	3 months	2 years	Placebo (*n* = 13)	Safety and tolerability, liver function, mortality.
Gupta N, 2013	India	NA	Cirrhotic patients having large esophageal varices	Probiotic: VSL#3 (*n* = 31)	2 months	2 months	Placebo (*n* = 32)	Safety and tolerability.
Horvath A, 2016	Austria	2012.7–2013.9	Cirrhosis	Probiotic: Bifidobacterium, Lactobacillus, and Lactococcus (*n* = 44)	6 months	12 months	Placebo (*n* = 36)	Safety and tolerability, liver function.
Jayakumar S, 2013	Canada	NA	Decompensated cirrhosis	Probiotic: VSL#3 (n = 7)	2 months	2 months	Placebo (n = 8)	Liver function.
Koga H, 2013	Japan	2005.10–2006.10	Alcoholic cirrhosis	Probiotic: beverage Yakult 400 (Y400) (*n* = 18)	2 weeks	1 month	Placebo (*n* = 19)	Effect on gut flora.
Liu Q, 2004	China	NA	Cirrhosis with MHE	Synbiotic preparation: consisiting of 4 bacteria along with fermentable fiber (*n* = 20)	1 month	1 month	Fermentable fiber (*n* = 20)	HE reversal, liver function, effect on gut flora, inflammatory cytokines change.
Loguercio C, 1987	Italy	NA	Cirrhosis	Probiotic: Enterococcus SF68 (*n* = 20)	10 days	20 days	Lactulose (*n* = 20)	HE reversal.
Loguercio C, 1995	Italy	NA	Cirrhotic patients with HE	Probiotic: Enterococcus SF68 (*n* = 21)	3 months	3 months	Lactulose (*n* = 19)	HE reversal.
Lunia M K, 2014	India	2012.1–2013.3	Cirrhosis	Probiotic: VSL#3 (*n* = 86)	3 months	3 months	Standard treatment (*n* = 74)	HE reversal, safety and tolerability, mortality.
Macnaughtan J, 2020	UK	NA	Cirrhosis	Probiotic: *Lactobacillus casei* Shirota (*n* = 44)	6 months	6 months	Placebo (*n* = 43)	Safety and tolerability, quality of life.
Manzhalii E, 2022	Ukraine	2017.1–2020.3	Cirrhosis with MHE	Probiotic: *Escherichia coli* Nissle 1917 strain (*n* = 15)	1 month	1 month	(1)Lactulose (*n* = 15) (2)Rifaximin (*n* = 15)	HE reversal.
Maslennikov R, 2022	Russia	NA	Consecutive cirrhosis	Probiotics:Saccharomyces boulardii (*n* = 24)	3 months	3 months	Placebo (*n* = 16)	HE reversal, safety and tolerability, liver function.
Mittal V V, 2011	India	2007.10–2009.10	Cirrhosis with MHE	Probiotics: subtype not available (*n* = 40)	3 months	3 months	Standard treatment (*n* = 40)	HE reversal, safety and tolerability.
Pande C, 2012	India	2005.4–2007.8	Cirrhotic patients with ascites	Probiotics: *E. faecalis* JPC, *C. butyricum*, B. mesentericus JPC, *Bacillus coagulans* (*n* = 55)	6 months	6 months	Placebo (*n* = 55)	Safety and tolerability, mortality.
Pereg D, 2011	Israel	NA	Cirrhosis	Probiotic: Lactobacillus, Bifidobacterium, and Streptococcus (*n* = 20)	6 months	6 months	Placebo (*n* = 20)	HE reversal, safety and tolerability, liver function.
Pratap Mouli V, 2015	India	2009.10–2012.6	Cirrhosis with MHE	Probiotic: VSL#3 (*n* = 33)	2 months	2 months	Lactulose (*n* = 40)	HE reversal, safety and tolerability, mortality.
Ramachandran G, 2023	India	2021.7–2022.10	Cirrhosis	Probiotics: VSL#3 (*n* = 108)	6 weeks	6 weeks	Placebo (*n* = 107)	Safety and tolerability, liver function, quality of life, mortality.
Roman E, 2019	Spain	2013.2–2016.3	Consecutive outpatients with cirrhosis	Probiotic: Streptococcus, Bifidobacterium, Lactobacillus (*n* = 18)	3 months	5 months	Placebo (*n* = 18)	Safety and tolerability, liver function, quality of life, mortality.
Saji S, 2011	India	NA	Cirrhosis with MHE	Probiotic: Lactobacillus, Bifidobacterium, and Sacharomyces (*n* = 21)	1 month	1 month	Placebo (*n* = 22)	Safety and tolerability.
Sharma K, 2014	India	2009.8–2010.8	Cirrhosis with MHE	Probiotics (*n* = 32)	2 months	2 months	Placebo (*n* = 30)	HE reversal, mortality.
Sharma P, 2008	India	2005.2–2006.8	Cirrhosis with MHE	Probiotics: *Streptococcus faecalis*, *Clostridium butyricum*, Bacillus mesentricus, lactic acid bacillus (*n* = 35)	1 month	1 month	Lactulose (*n* = 35)	HE reversal, liver function.
Shavakhi A, 2014	Iran	2012.6–2012.10	Cirrhosis with MHE	Synbiotics: Probiotic and Lactulose (*n* = 19)	2 weeks	10 weeks	Lactulose+Placebo (*n* = 21)	Safety and tolerability.
Shi J, 2023	China	2020.8–2021.8	Cirrhosis with MHE	Synbiotics: Probiotic and Lactulose (*n* = 44)	2 weeks	2 weeks	Lactulose (*n* = 44)	HE reversal, liver function, inflammatory cytokines change.
Xia X, 2018	China	NA	Cirrhosis with MHE	Probiotics: *Clostridium butyricum* and *Bifidobacterium infantis* (n = 30)	3 months	3 months	Standard treatment (*n* = 37)	HE reversal, safety and tolerability, liver function.
Zhao XH, 2013	China	NA	Cirrhosis with MHE	Probiotic (*n* = 40)	1 month	1 month	Placebo (*n* = 40)	HE reversal, safety and tolerability.
Ziada DH., 2013	Egypt	2010.3–2012.1	Cirrhosis with MHE	Probiotic: L. acidobacillus acidophilus (*n* = 30)	1 month	1 month	Lactulose (*n* = 30)	HE reversal, liver function, effect on gut flora.

### 3.3 Probiotics reverse HE associated with liver cirrhosis

**Figure 2:**
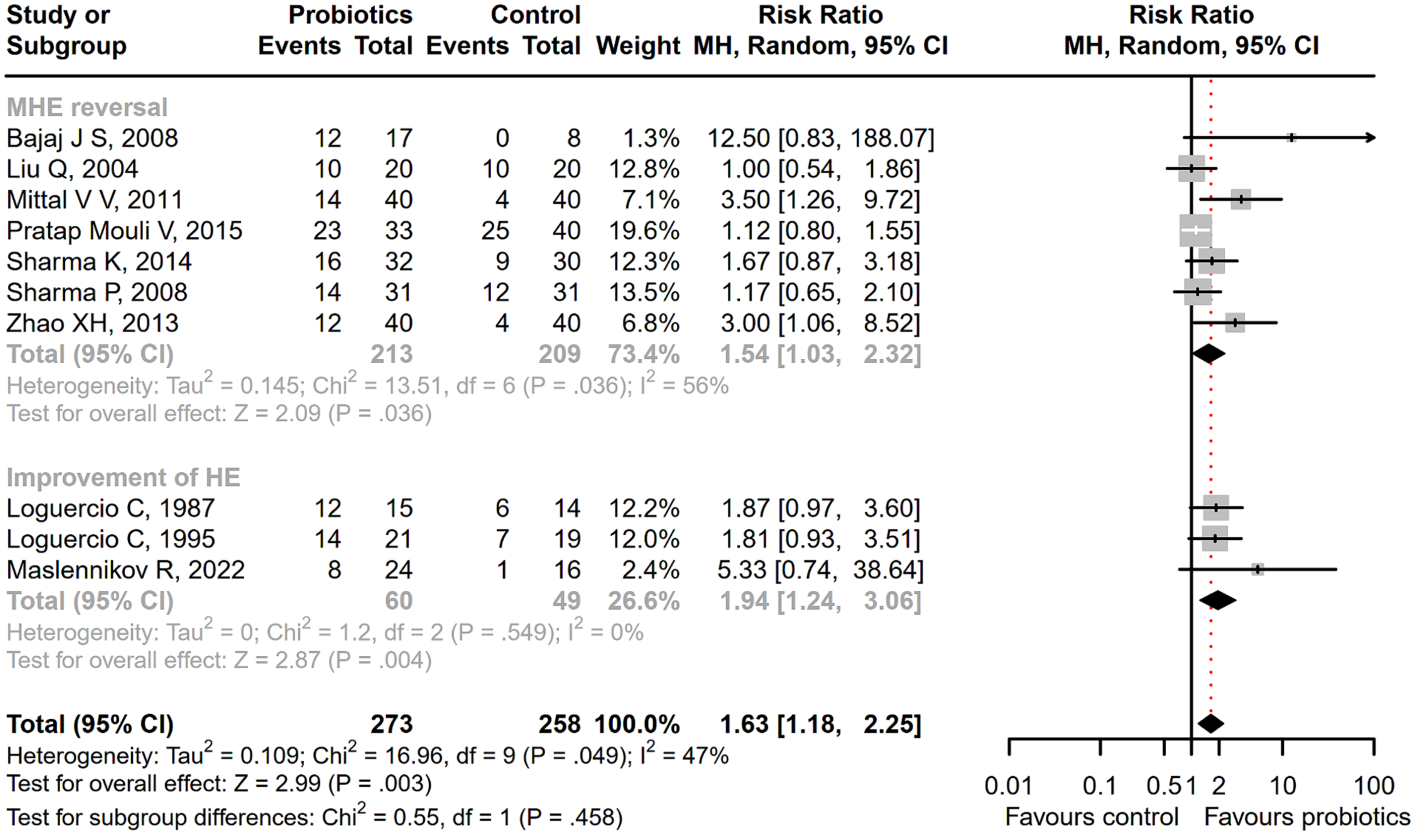
The effect of probiotics on the incidence of MHE and HE. Probiotics could promote MHE reversal and HE improvement.

**Figure 3:**
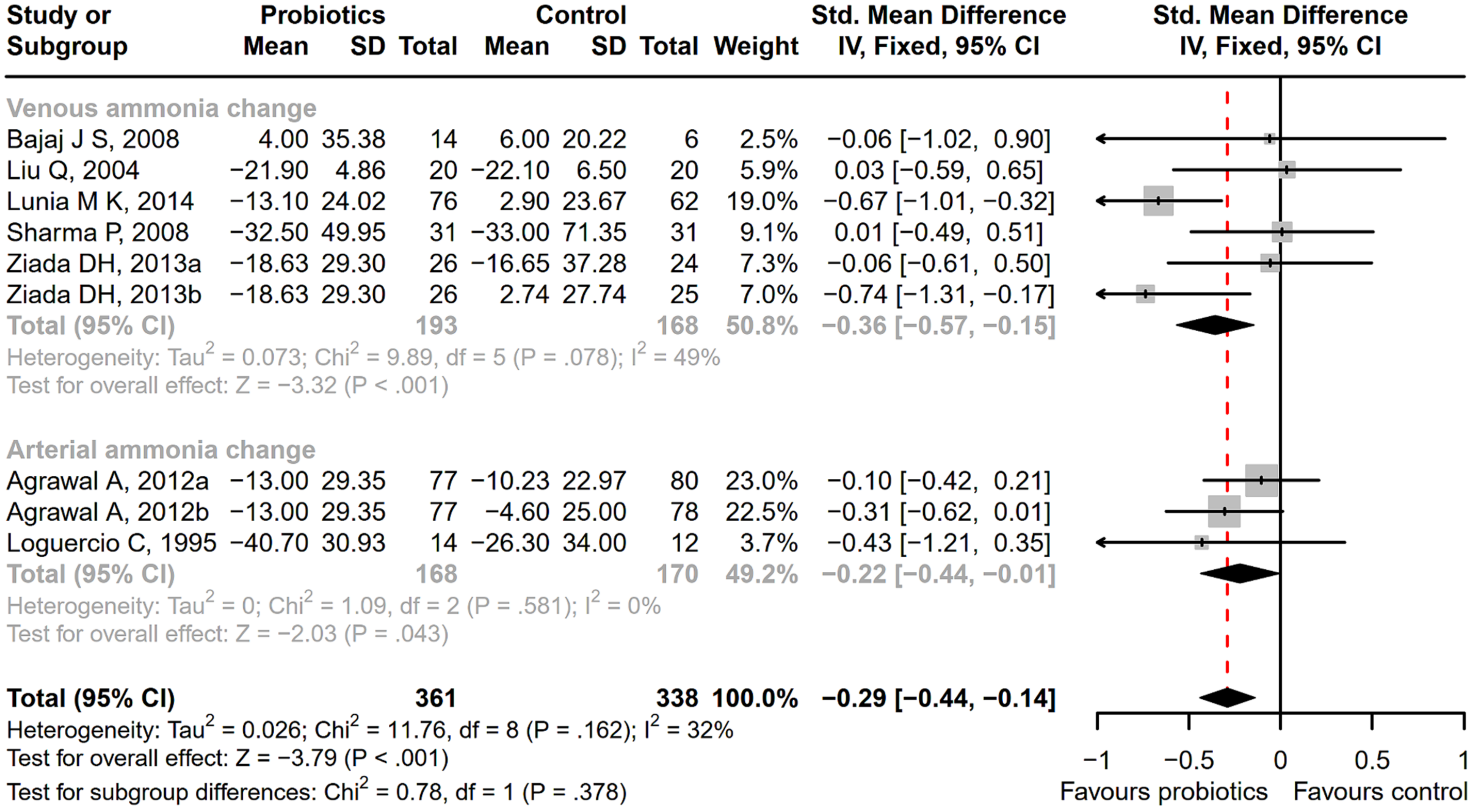
The effect of probiotics on the ammonia level. There was a significant decrease in both venous and arterial ammonia level among the patients receiving probiotics.

**Figure 4:**
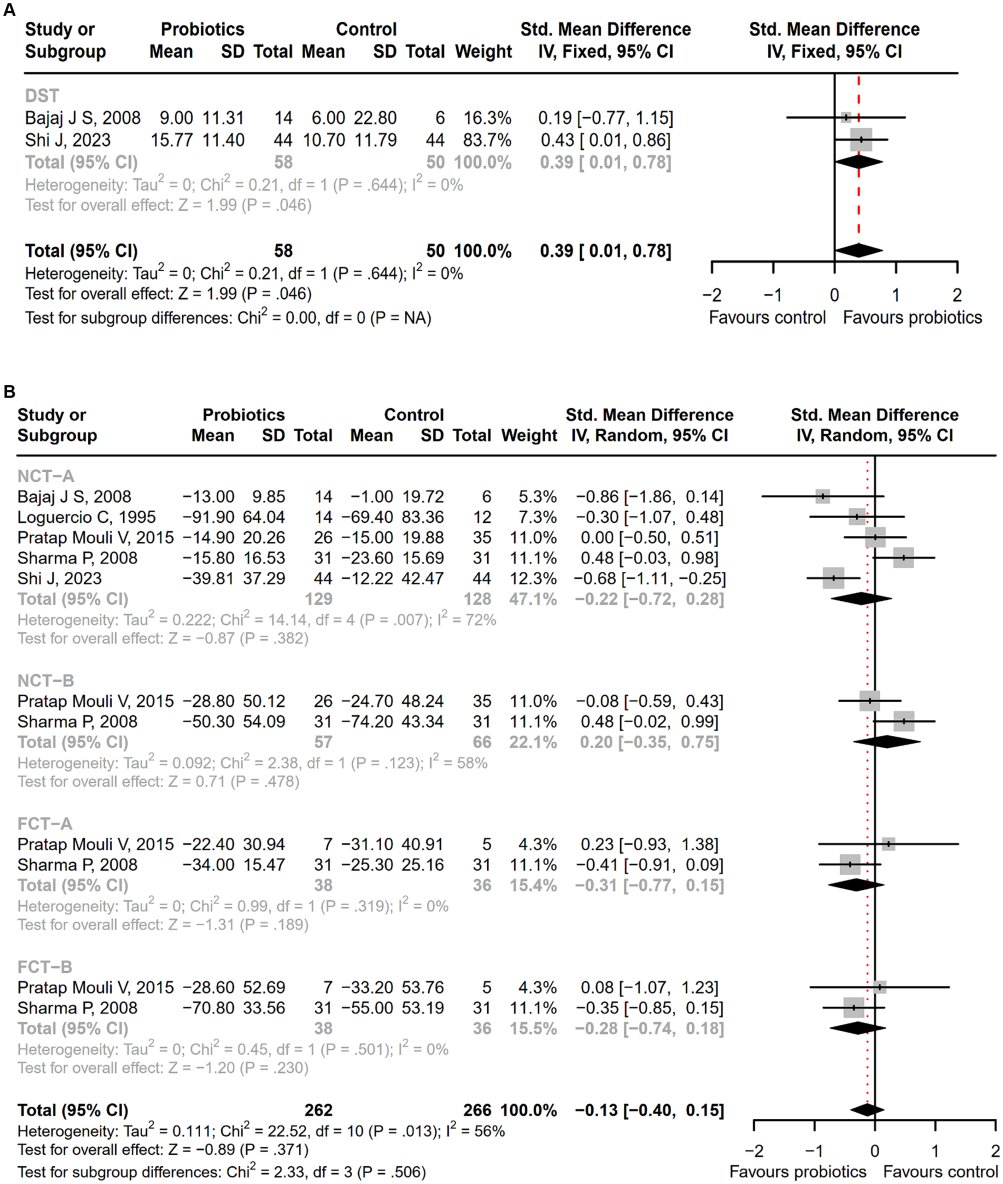
The forest plot of neuropsychometric assessments. **(A)** DST; **(B)** NCT-A, NCT-B, FCT-A, FCT-B. Notable higher DST scores were shown in the probiotic group.

**Figure 5:**
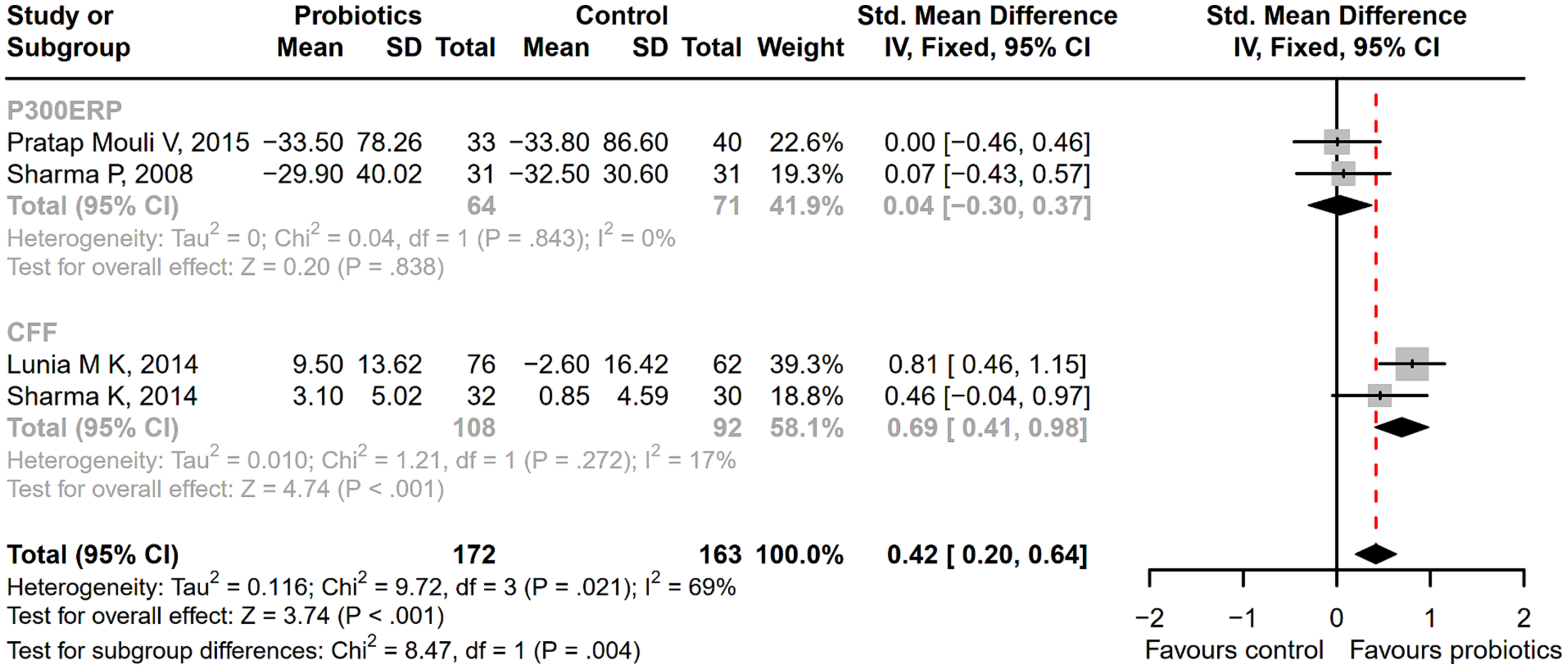
The forest plot of neurophysiological tests of P300 auditory event-related potential (P300ERP) and critical flicker frequency (CFF). A significant improvement in critical flicker frequency (CFF) was observed in the probiotic group.

Seventeen studies were conducted among cirrhotic patients with different stages of HE (26, 27, 37–40, 42, 44–48, 50–54). Parameters containing ammonia level and neuropsychometric or neurophysiological status were measured to evaluate the improvement of HE. Results demonstrated that probiotic intervention could reverse MHE (RR = 1.54; 95% CI, 1.03 to 2.32; *p* < 0.05) and improve HE (RR = 1.94; 95% CI, 1.24 to 3.06; *p* < 0.01) significantly (Figure 2). Based on the subgroup analysis of probiotic types, the results show that compared to other types of probiotics, the VSL#3 probiotic (containing *Streptococcus*, *Bifidobacterium*, and *Lactobacillus*) has a more significant improvement effect on HE (RR = 1.44; 95% CI, 1.00 to 2.07; *p* < 0.05; Figure S1). A notable reduction was detected in both venous (SMD = −0.36; 95% CI, −0.57 to −0.15; *p* < 0.001) and arterial ammonia levels (SMD = −0.22; 95% CI, −0.44 to −0.01; *p* < 0.05) within the probiotic group versus the control group (Figure 3). The results of the subgroup analysis of time of ammonia showed that, with the extension of follow-up time, the reduction level of ammonia was more significant at 3 months (SMD = −0.34; 95% CI, −0.56 to −0.11; *p* < 0.01; Figure S2). According to the neuropsychometric tests, significant higher digit symbol test (DST) scores were shown among cirrhotic patients with MHE in the probiotic group (SMD = 0.39; 95% CI, 0.01 to 0.78; *p* < 0.05; Figure 4A), while no significant change was detected in the number connection test (NCT) and figure connection test (FCT) (Figure 4B). A significant improvement in critical flicker frequency (CFF) was observed in the probiotic group based on neurophysiological test results (SMD = 0.69; 95% CI, 0.41 to 0.98; *p* < 0.001; Figure 5). Sensitivity analysis showed the overall effect size of venous ammonia would be impacted by removing a single effect size (Supplementary Figure S3).

### 3.4 Probiotics exhibit higher safety and tolerability

**Figure 6:**
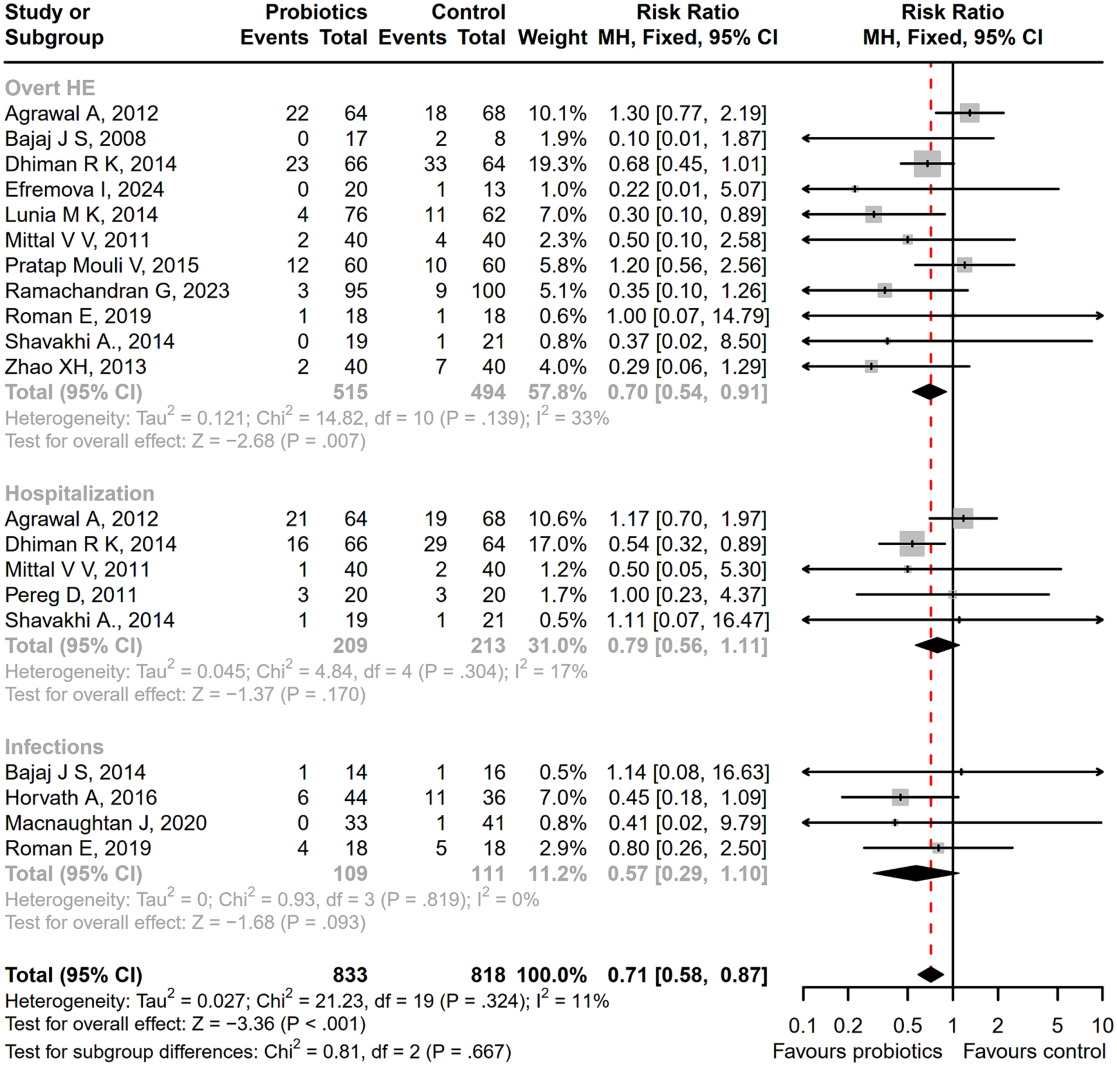
The forest plot of the serious adverse events incidence. There was a significant lower incidence of overt HE, hospitalization, and infections among patients receiving probiotic treatment, compared with the control treatment.

**Figure 7:**
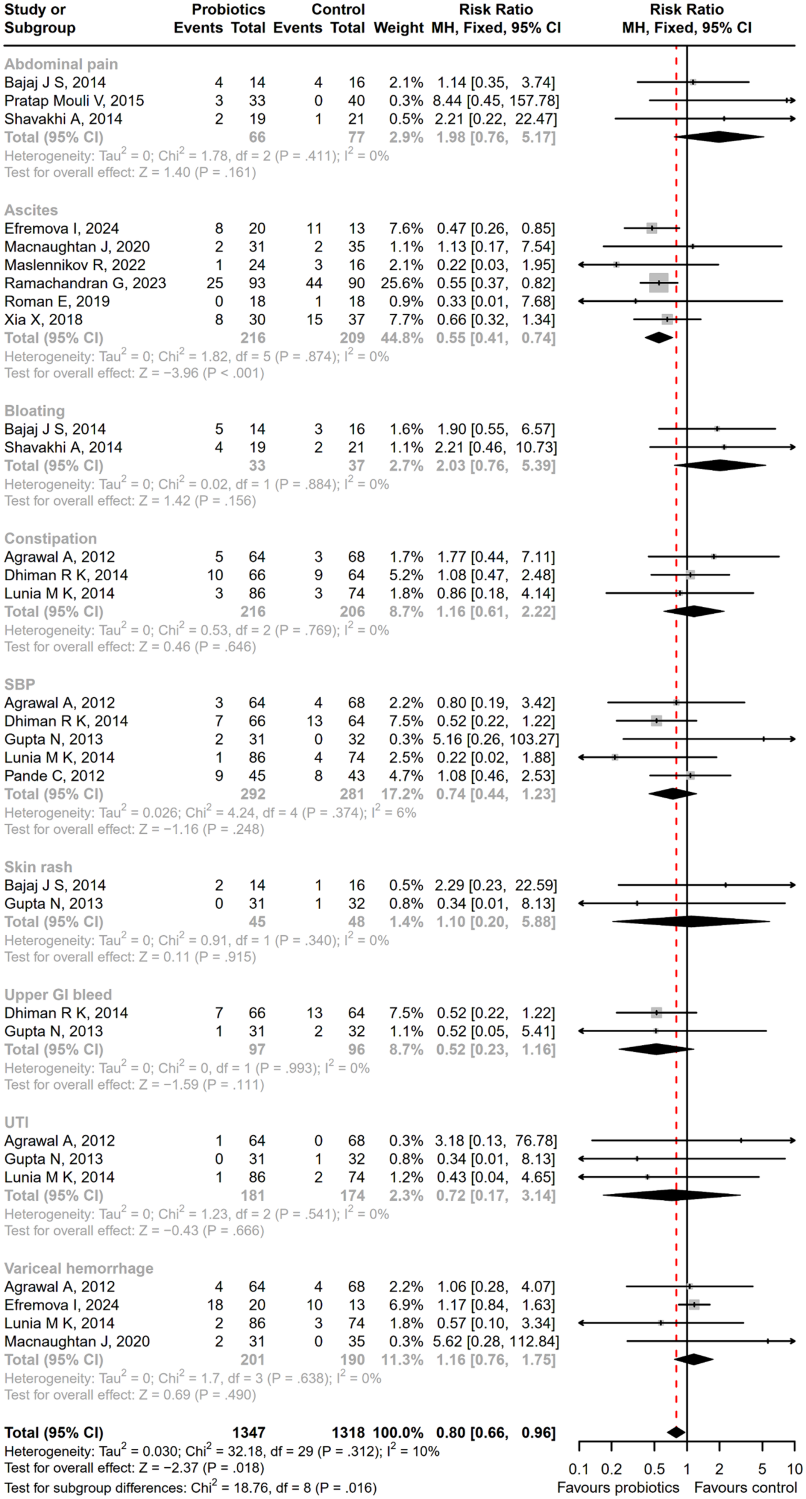
The forest plot of the adverse events incidence. There was a significant lower incidence of ascites in the probiotic group, but no difference in the other adverse events such as abdominal pain, bloating, and constipation between groups.

Compared with the control treatment including lactulose, rifaximin, placebo, and standard therapy, there was a significantly lower incidence of serious adverse events among patients receiving probiotic treatment (RR = 0.71; 95% CI, 0.58 to 0.87; *p* < 0.001; Figure 6). As the intervention time extended, patients with overt HE development (RR = 0.64; 95% CI, 0.48 to 0.85; *p* < 0.01; Supplementary Figure S4), hospitalization (RR = 0.58; 95% CI, 0.36 to 0.93; *p* < 0.05; Supplementary Figure S5), and infections (RR = 0.44; 95% CI, 0.29 to 0.66; *p* < 0.001; Supplementary Figure S6) decreased more significantly after a 6-month follow-up. A significant reduction in the incidence of ascites was reported in the probiotic group compared to the placebo or standard treatment (RR = 0.55; 95% CI, 0.41 to 0.74; *p* < 0.001), but there was no difference in abdominal pain, bloating, constipation, or other adverse events between groups (Figure 7).

According to the pooled result of adherence, the nonadherent rate was 6.75% in the probiotic group, whereas it was 6.4% in the control group, showing no significant difference between groups (Supplementary Figure S7).

### 3.5 Probiotics potentially improve liver function in patients with liver cirrhosis

**Figure 8:**
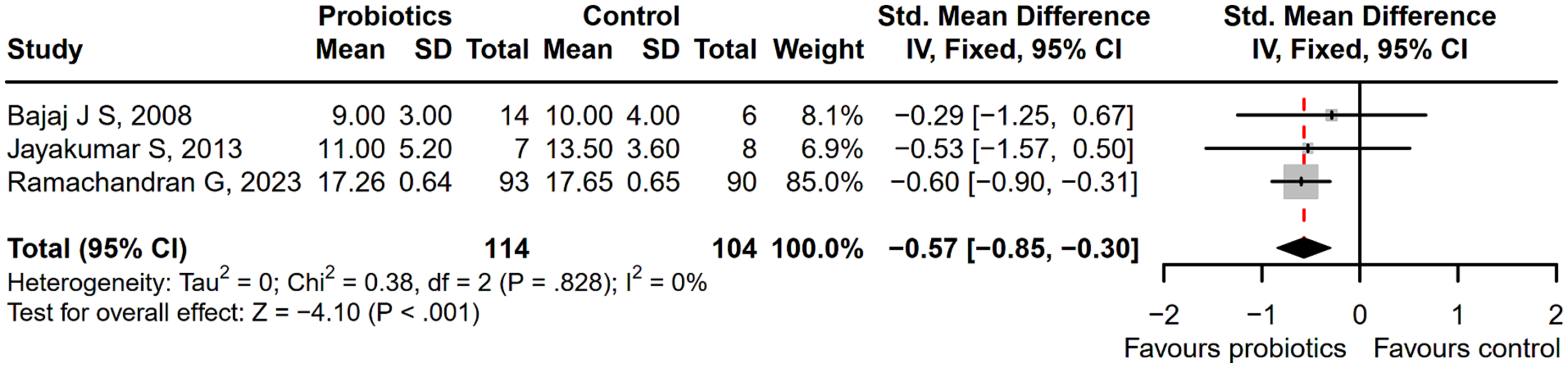
The effect of probiotics in MELD scores. The probiotics could significantly reduced the MELD scores, compared with control treatment.

The probiotic group demonstrated a statistically significant reduction in Model for End-Stage Liver Disease (MELD) scores compared with the control group (SMD = −0.57; 95% CI, −0.85 to −0.30; *p* < 0.001; Figure 8). The measurements of MELD containing international normalized ratio (INR), creatinine, and total bilirubin (TBIL) were shown in Supplementary Figure S8. However, the serum sodium levels of the probiotic group were still significantly lower than those of the control group (Supplementary Figure S9). And there was no difference in another liver function parameters in the blood (Supplementary Figure S10) and the Child-Turcotte-Pugh (CTP) classification (Supplementary Figure S11) between the probiotic group and the control group. The levels of liver function parameters were tested at different treatment time points, and the results showed that as the intervention time prolonged, the levels of parameters showed a continuous downward trend, but statistically, it was not significant (Supplementary Figure S12). Sensitivity analysis showed the overall effect size of INR would be impacted by removing a single effect size (Supplementary Figure S13).

### 3.6 Probiotics induce favorable changes on quality of life and gut flora

**Figure 9:**
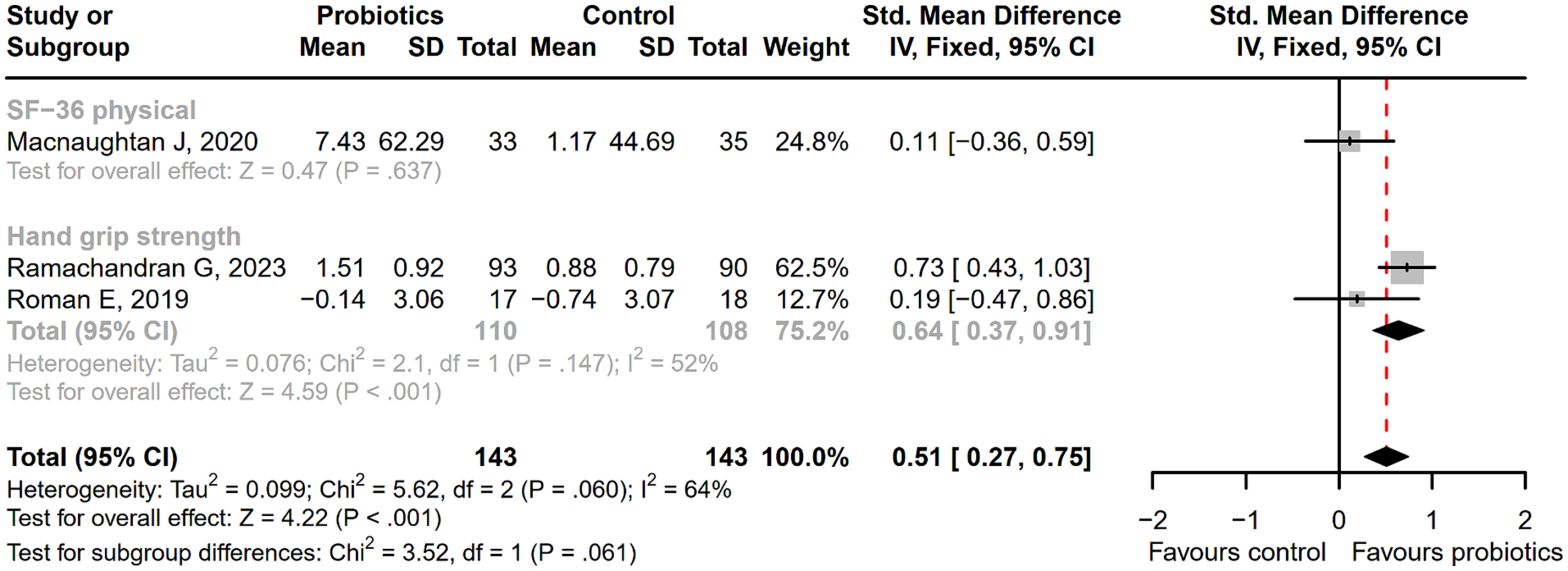
The forest plot of quality of life. The quality of life score of cirrhotic patients significantly improved in the probiotic group.

**Figure 10:**
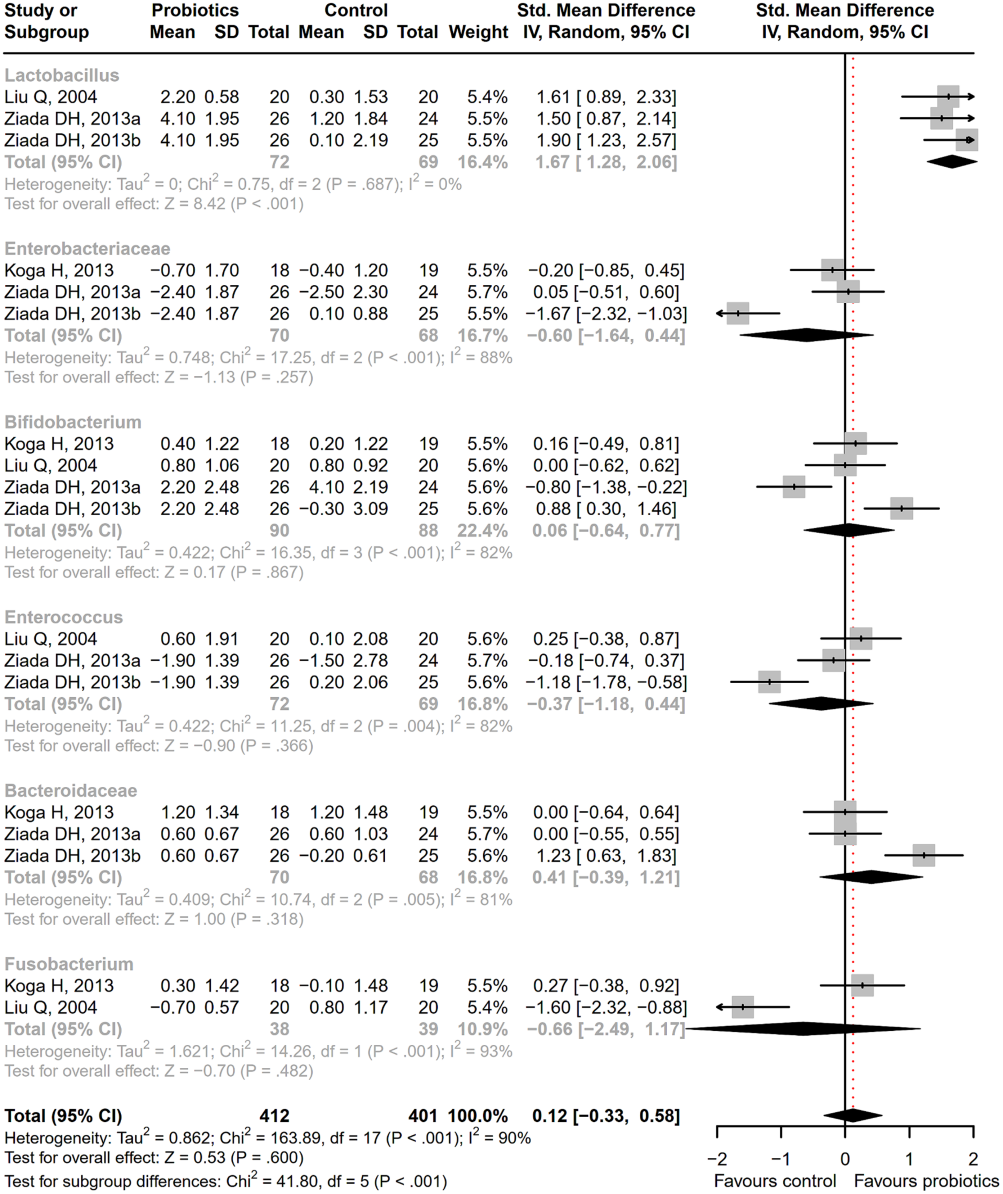
The effect of probiotics on gut flora. The numbers of *Lactobacillus* group were significantly increased.

After receiving probiotic treatment, the quality of life score of patients with cirrhosis significantly improved (SMD = 0.51; 95% CI, 0.27 to 0.75; *p* < 0.001; Figure 9). The numbers of the *Lactobacillus* group were significantly increased after probiotic treatment (SMD = 1.67; 95% CI, 1.28 to 2.06; *p* < 0.001), while the numbers of *Enterobacteriaceae*, *Bifidobacterium*, *Enterococcus*, *Bacteroidaceae*, and *Fusobacterium* did not differ significantly between the probiotic and control groups (Figure 10). Sensitivity analysis of quality of life (Supplementary Figure S14) showed that the overall effect size could be influenced by removing a single effect size, whereas the overall effect size remained unaffected by the removal of a single effect size in the results of gut flora (Supplementary Figure S15).

### 3.7 Probiotics have no significant effect on inflammatory cytokines expression and mortality

Among cirrhotic patients receiving probiotics, there was a numerical but not prominent decrease in serum inflammatory cytokine expression, including endotoxin, interleukin (IL)-6, and tumor necrosis factor (TNF)-α (Supplementary Figure S16). Meanwhile, there was a numerical but non-significant decline in mortality (Supplementary Figure S17). The overall effect size was not influenced by removing a single effect size, according to the sensitivity analysis of IL-6 (Supplementary Figure S18).

### 3.8 Quality assessment and publication bias

Two studies reported a low overall risk of bias. High risk of bias was most represented in the domains of blinding of participants and personnel, and blinding of outcome assessment (Supplementary Figure S19). The detailed support for the judgment of the risk of bias in each included study was shown in Supplementary Table S4. Egger’ s regression test or Peter’s test showed there was no publication bias in the results of the main findings containing HE reversal, safety and tolerability of probiotics, liver function, and gut microbial taxonomy (*p* > 0.05) (Supplementary Figure S20).

## 4 Discussions

In recent years, numerous clinical trials have employed probiotics as a treatment for various liver diseases, encompassing conditions such as liver cirrhosis (32), nonalcoholic fatty liver disease (NAFLD) (55), and HE (56). Probiotic therapy has been systematically analyzed for its effects on NAFLD and HE, and previous reviews have demonstrated its efficacy as a therapeutic strategy (57, 58). While there have been synthesis analyses examining the effects of probiotics in patients with liver cirrhosis, these analyses were either limited to cirrhotic patients with MHE (18) or were not conducted exclusively based on randomized clinical trials (20). The available evidence regarding the effects of probiotics on the course of cirrhosis is limited. This is a critical aspect of potentially reversing the onset of cirrhosis in its early stages and preventing further disease progression. This systematic review and meta-analysis comprehensively investigated the effectiveness of probiotic interventions in cirrhosis through the synthesized analysis of RCTs, representing the pinnacle of the evidence pyramid. The findings indicated that probiotics may mitigate the negative effects of cirrhosis by reversing cirrhotic HE, potentially improving liver function, and fostering favorable changes in quality of life and gut microbial taxa. Moreover, probiotic interventions appeared to exhibit a higher level of safety and tolerability.

HE is often a complication of advanced liver dysfunction, especially cirrhosis, causing mental confusion due to the buildup of toxins in the brain. One of the toxins affecting the brain is ammonia. Elevated ammonia levels are believed to be the culprit in the pathogenesis of HE (59). This systematic review and meta-analysis revealed that, in comparison to treatment measures in the control group such as lactulose and placebo, probiotic intervention had a notably beneficial effect on reducing ammonia levels in the blood. A neuropsychometric test is an important tool to diagnose different grades of HE, including DST, block design test (BDT), NCT-A, NCT-B, line tracing test (LTT), and serial dotting test (SDT) (60). This study demonstrated an enhancement in the neuropsychometric status of cirrhotic patients with HE after receiving probiotics, as evidenced by lower DST scores. And the significant improvements in CFF prove enhancements in the neurophysiological status among cirrhotic patients after receiving probiotics. These results suggest that probiotics have the potential to ameliorate the condition of patients with HE. This is further supported by our meta-analysis, which revealed a significant reversal of MHE and improvement of HE among patients in the probiotic group. Additionally, VSL#3, as a commonly used probiotic for repairing the intestinal barrier, has been proven to play a positive role in the treatment of multiple diseases (61, 62). Our research also found that VSL#3 could play an effective role in improving HE, suggesting it might be considered as a priority choice for probiotic treatment to reverse HE in the future.

Probiotics are among the most commonly used dietary supplements. It showed good tolerability, a low attrition rate, and no serious adverse reactions in many clinical trials for a variety of diseases (63, 64). Although many probiotics are considered safe, with increasing usage among cirrhotic patients in clinics, there may be a greater need to assess their safety and tolerability. Our study revealed a lower incidence of adverse events or serious adverse events among patients receiving probiotics compared to those receiving lactulose, placebo, or standard treatment. This suggests good tolerability and a high likelihood of safety of probiotics in cirrhosis. There was no significant difference in nonadherence rates between the groups, indicating that patients were able to tolerate and remain compliant with probiotic therapy. This finding is conducive to the further promotion and expansion of the use of probiotics.

Liver cirrhosis is characterized by liver scarring, impaired liver function, and other side effect (65). The MELD score and CTP classification are extensively employed for the evaluation of liver function, where a higher score indicates more pronounced functional impairment. They have been widely used for the assessment of prognosis in liver cirrhosis (66). Our study revealed no discernible difference in CTP classification between the intervention and control groups. This finding may not offer robust evidence regarding the effect of probiotic intervention, considering that the parameters of the CTP classification incorporate subjective indicators such as ascites and encephalopathy. In contrast, MELD scores are calculated based on objective indicators, including INR, TBIL, and creatinine, demonstrating enhanced predictive capabilities for liver function and providing a reliable assessment of cirrhosis severity (67). In this systematic review and meta-analysis, a reduction in the MELD score was observed after probiotic intervention. However, the statistical significance of the changes in MELD scores may not have reached clinical significance. Additionally, some liver function parameters, such as ALT and AST, did not exhibit statistically significant changes. This suggests that probiotics might have the potential to improve liver function among cirrhosis patients, but the clinical improvement effects still require further confirmation. The insufficient intervention time might also be an important reason for the poor clinical improvement of the indicators.

This study also investigated the expression levels of serum sodium, an important indicator reflecting the liver function status among cirrhotic patients. In patients with cirrhosis, the abnormal activity of the antidiuretic hormone system may lead to a disruption in sodium metabolism, which could result in the occurrence of hyponatremia (68). This study found that hyponatremia did not improve after probiotic treatment. This phenomenon may be attributed to the insufficient adoption of an evidence base for this meta-analysis, potentially impacting the pooled outcomes adversely. Moreover, in this study, the intervention duration of the existing studies for analyzing the effects of probiotics on serum sodium was less than 3 months, consistent with the assessment time of MELD scores. An important finding observed from this study showed that at least 3 months of probiotic intervention were required for yielding favorable outcomes on indicators including ammonia levels and the incidence of adverse events. With the extension of the follow-up time, the impact of probiotics became more significant. This suggests that a certain duration is necessary for probiotics to exert their beneficial effects. And as the intervention and follow-up time prolong, the efficacy of probiotics becomes increasingly significant. Therefore, due to the limitations of the existing evidence base, we failed to identify significant clinical improvement effects in liver function among cirrhotic patients after probiotic intervention. However, the statistically significant changes in MELD scores still suggest that probiotics have the potential to enhance liver function. Future probiotic intervention studies could consider concentrating on these indicators to furnish more evidence regarding liver function changes among cirrhotic patients.

The heightened secretion of endotoxin, a bacterial product, induced by an imbalance in the gut microbiota among patients with cirrhosis, results in liver damage (69). Endotoxin can exacerbate liver damage by amplifying the release of inflammatory factors (70). Therefore, regulating the gut microbial profile is crucial for preventing cirrhosis progression. The results of this study revealed favorable alterations in the stool microbial profile, characterized by an increase in beneficial bacteria *Lactobacillus*. The changes could contribute to the reduction of endotoxin levels and inflammatory factors such as TNF-α or interleukin (IL)-6, aligning with the findings of this systematic review and meta-analysis. However, the results of the study showed that some intestinal flora and inflammatory cytokine disturbances were not significantly restored, which might also be the reason why the liver function of cirrhotic patients did not improve clinically after probiotic intervention.

Quality of life in cirrhotic patients is significantly impaired by the disease manifestations and complication (71, 72). Our study found a notable enhancement in the quality of life in cirrhosis patients, which might be correlated with the decrease in the incidence of adverse events after receiving probiotic treatment. However, we failed to detect a significant reduction in mortality. This might be attributed to the fact that the effects of probiotics on this indicator require an extended follow-up time to be apparent. Most of the included studies had a follow-up duration of around 3 months, which might not be sufficient to observe significant improvement effects. Therefore, future studies could extend the follow-up period to observe more objective outcomes that support the beneficial effects of probiotics.

Despite the significance of this systematic review and meta-analysis, several limitations need to be acknowledged. Firstly, among the 30 studies included, only 2 exhibited a low risk of bias across all seven domains, while the remaining 28 showed a high or unclear risk of bias for at least one bias domain. Secondly, there is a scarcity of available evidence base on the intervention effect of various types of probiotics or different duations of treatment in cirrhosis, preventing a more comprehensive comparison of the impact among probiotics or treatment durations on cirrhosis. Lastly, although RCTs were incorporated in this study, and the results obtained possessed a high level of evidence, some results lacked a sufficient number of included studies for a comprehensive meta-analysis, limiting the depth of our analysis. Future studies should strive to address these gaps, maximizing the utilization of probiotics to promote cirrhosis reversal and prevention.

## 5 Conclusion

This systematic review and meta-analysis provides compelling evidence supporting the benefits of probiotics in cirrhosis. Probiotics contribute to the reduction of ammonia levels and the improvement of neuropsychometric or neurophysiological status, leading to the reversal of HE associated with cirrhosis. They exhibit higher safety and tolerability, as evidenced by a significant lower incidence of serious adverse events compared with the control treatment. Probiotics demonstrate the potential to enhance liver function by down-regulating the MELD score. Moreover, they induce favorable changes in gut flora and quality of life. Therefore, probiotics emerge as a promising intervention for reversing the onset of cirrhosis and preventing disease progression.

## Data availability statement

All data relevant to the study are included in the article or uploaded as Supplementary information.

## Author contributions

XY: Data curation, Investigation, Methodology, Software, Writing – original draft, Writing – review & editing. LLe: Data curation, Investigation, Methodology, Software, Writing – original draft. WS: Data curation, Methodology, Software, Validation, Writing – review & editing. XL: Funding acquisition, Methodology, Resources, Validation, Writing – review & editing. XH: Data curation, Methodology, Writing – original draft. LLa: Data curation, Methodology, Software, Writing – original draft. JL: Methodology, Software, Writing – original draft. QL: Data curation, Methodology, Writing – original draft. WL: Funding acquisition, Project administration, Supervision, Validation, Writing – review & editing. JY: Conceptualization, Funding acquisition, Investigation, Project administration, Resources, Supervision, Writing – review & editing.

## Footnotes

^1^ http://CRAN.R-project.org/package=meta

^2^ https://cran.r-project.org/package=robvis
