# Supplementary material for: Probiotics are beneficial for liver cirrhosis: a systematic review and meta-analysis of randomized control trials
Source: Front Med (Lausanne). 2024 Mar 28;11:1379333. doi: 10.3389/fmed.2024.1379333 (PMC11010643; doi:10.3389/fmed.2024.1379333)
Supplement: Supplementary file 1 [file Data_Sheet_1.PDF]

Study or Subgroup	Probiotics Events	Probiotics Total	Control Events	Control Total	Weight	Risk Ratio MH, Fixed, 95% CI
**VSL#3**						
Bajaj J S, 2008	12	17	0	8	1.8%	12.50 [0.83, 188.07]
Pratap Mouli V, 2015	23	33	25	40	61.4%	1.12 [0.80, 1.55]
**Total (95% CI)**		**50**		**48**	**63.2%**	**1.44 [1.00, 2.07]**
Heterogeneity: $Tau^2 = 3.665$; $Chi^2 = 4.78$, df = 1 (P = .029); $I^2 = 79\%$						
Test for overall effect: Z = 1.98 (P = .048)						
**Enterococcus SF68**						
Loguercio C, 1987	9	15	6	14	16.9%	1.40 [0.67, 2.91]
Loguercio C, 1995	14	21	7	19	20.0%	1.81 [0.93, 3.51]
**Total (95% CI)**		**36**		**33**	**36.8%**	**1.62 [0.99, 2.65]**
Heterogeneity: $Tau^2 = 0$; $Chi^2 = 0.26$, df = 1 (P = .610); $I^2 = 0\%$						
Test for overall effect: Z = 1.93 (P = .053)						
**Total (95% CI)**		**86**		**81**	**100.0%**	**1.51 [1.13, 2.02]**
Heterogeneity: $Tau^2 = 0.115$; $Chi^2 = 5.90$, df = 3 (P = .117); $I^2 = 49\%$						
Test for overall effect: Z = 2.76 (P = .006)						
Test for subgroup differences: $Chi^2 = 0.14$, df = 1 (P = .704)						

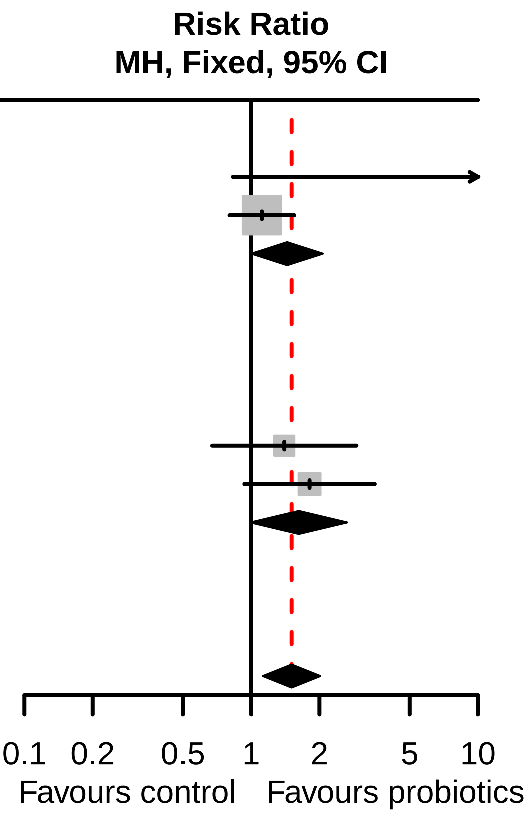

Figure S1. The forest plots of effects of different probiotic types on the HE improvement

Study or Subgroup	Probiotics Mean	Probiotics SD	Probiotics Total	Control Mean	Control SD	Control Total	Weight	Std. Mean Difference IV, Random, 95% CI
**less than 1 month**								
Liu Q, 2004	−21.90	4.86	20	−22.10	6.50	20	5.6%	0.03 [−0.59, 0.65]
Loguercio C, 1995	−29.00	35.78	18	−21.90	22.38	19	5.2%	−0.23 [−0.88, 0.41]
Pereg D, 2011	−7.30	38.08	18	0.90	34.67	18	5.1%	−0.22 [−0.88, 0.44]
Sharma P, 2008	−32.50	49.95	31	−33.00	71.35	31	7.8%	0.01 [−0.49, 0.51]
Shi J, 2023	−18.93	10.89	44	−8.68	10.24	44	9.1%	−0.96 [−1.40, −0.52]
Ziada DH, 2013	−18.63	29.30	26	−16.65	37.28	24	6.7%	−0.06 [−0.61, 0.50]
**Total (95% CI)**			**157**			**156**	**39.6%**	**−0.26 [−0.62, 0.09]**
Heterogeneity: $Tau^2 = 0.113$; $Chi^2 = 11.99$, df = 5 (P = .035); $I^2 = 58\%$								
Test for overall effect: Z = −1.45 (P = .148)								
**2 months**								
Bajaj J S, 2008	4.00	35.38	14	6.00	20.22	6	2.7%	−0.06 [−1.02, 0.90]
Loguercio C, 1995	−38.90	32.24	15	−26.10	28.84	16	4.5%	−0.41 [−1.12, 0.30]
**Total (95% CI)**			**29**			**22**	**7.2%**	**−0.28 [−0.86, 0.29]**
Heterogeneity: $Tau^2 = 0$; $Chi^2 = 0.33$, df = 1 (P = .567); $I^2 = 0\%$								
Test for overall effect: Z = −0.97 (P = .330)								
**3 months**								
Agrawal A, 2012a	−13.00	29.35	77	−10.23	22.97	80	13.5%	−0.10 [−0.42, 0.21]
Agrawal A, 2012b	−13.00	29.35	77	−4.60	25.00	78	13.4%	−0.31 [−0.62, 0.01]
Loguercio C, 1995	−40.70	30.93	14	−26.30	34.00	12	3.8%	−0.43 [−1.21, 0.35]
Lunia M K, 2014	−13.10	24.02	76	2.90	23.67	62	12.3%	−0.67 [−1.01, −0.32]
Pereg D, 2011	−7.50	40.44	18	−1.20	33.45	18	5.1%	−0.17 [−0.82, 0.49]
**Total (95% CI)**			**262**			**250**	**48.1%**	**−0.34 [−0.56, −0.11]**
Heterogeneity: $Tau^2 = 0.022$; $Chi^2 = 5.98$, df = 4 (P = .201); $I^2 = 33\%$								
Test for overall effect: Z = −2.90 (P = .004)								
**6 months**								
Pereg D, 2011	−6.40	37.42	18	2.10	32.34	18	5.1%	−0.24 [−0.89, 0.42]
Test for overall effect: Z = −0.71 (P = .478)								
**Total (95% CI)**			**466**			**446**	**100.0%**	**−0.31 [−0.47, −0.14]**
Heterogeneity: $Tau^2 = 0.027$; $Chi^2 = 18.40$, df = 13 (P = .143); $I^2 = 29\%$								
Test for overall effect: Z = −3.63 (P < .001)								
Test for subgroup differences: $Chi^2 = 0.18$, df = 3 (P = .981)								

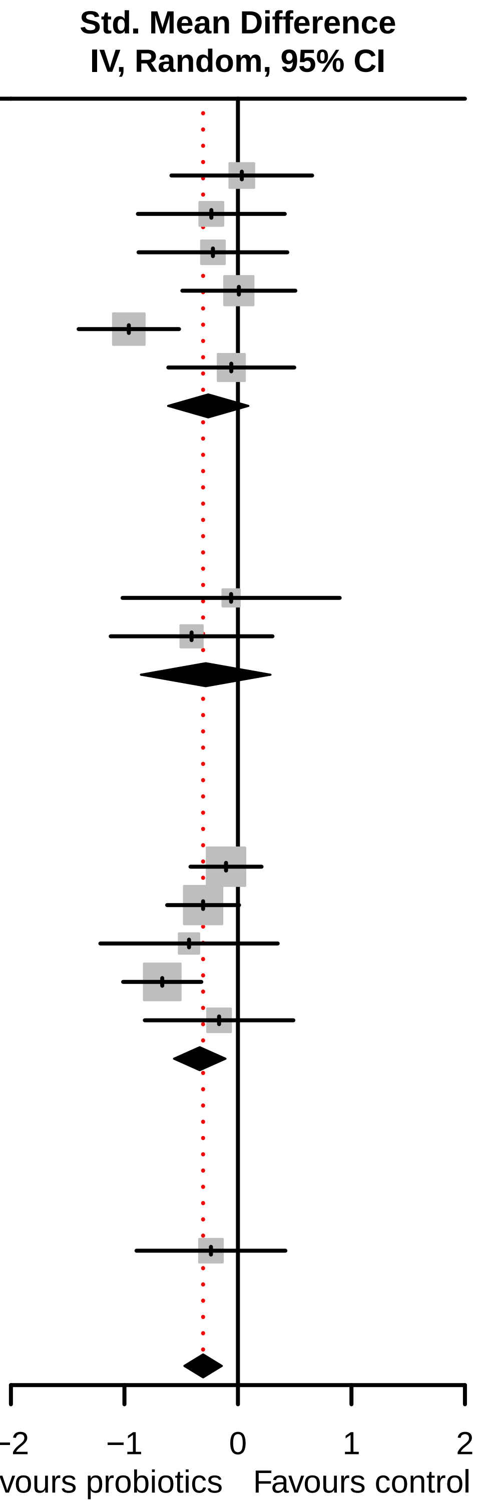

Figure S2. The forest plots of ammonia level tested in different follow-up time-point

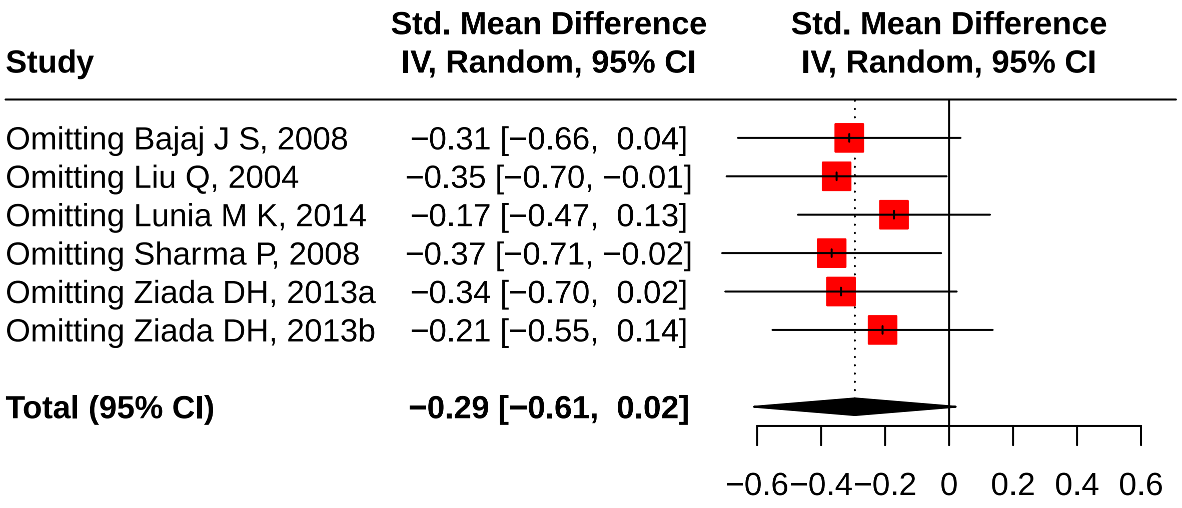

Figure S3. Sensitivity analysis of venous ammonia

Study or Subgroup	Probiotics Events	Probiotics Total	Control Events	Control Total	Weight	Risk Ratio MH, Fixed, 95% CI
**1 month**						
Bajaj J S, 2008	0	17	1	8	1.8%	0.17 [0.01, 3.70]
Zhao XH, 2013	2	40	7	40	6.4%	0.29 [0.06, 1.29]
**Total (95% CI)**		**57**		**48**	**8.2%**	**0.26 [0.07, 1.00]**
Heterogeneity: $Tau^2 = 0$; $Chi^2 = 0.09$, df = 1 (P = .759); $I^2 = 0\%$						
Test for overall effect: Z = −1.96 (P = .050)						
**2 months**						
Bajaj J S, 2008	0	17	2	8	3.0%	0.10 [0.01, 1.87]
Pratap Mouli V, 2015	12	60	10	60	9.1%	1.20 [0.56, 2.56]
**Total (95% CI)**		**77**		**68**	**12.2%**	**0.92 [0.46, 1.85]**
Heterogeneity: $Tau^2 = 1.987$; $Chi^2 = 2.67$, df = 1 (P = .102); $I^2 = 63\%$						
Test for overall effect: Z = −0.22 (P = .825)						
**3 months**						
Lunia M K, 2014	4	76	11	62	11.0%	0.30 [0.10, 0.89]
Mittal V V, 2011	2	40	4	40	3.6%	0.50 [0.10, 2.58]
Roman E, 2019	1	18	1	18	0.9%	1.00 [0.07, 14.79]
**Total (95% CI)**		**134**		**120**	**15.6%**	**0.39 [0.17, 0.90]**
Heterogeneity: $Tau^2 = 0$; $Chi^2 = 0.8$, df = 2 (P = .671); $I^2 = 0\%$						
Test for overall effect: Z = −2.21 (P = .027)						
**over 6 months**						
Agrawal A, 2012	22	64	37	65	33.5%	0.60 [0.41, 0.90]
Dhiman R K, 2014	23	66	33	64	30.6%	0.68 [0.45, 1.01]
**Total (95% CI)**		**130**		**129**	**64.0%**	**0.64 [0.48, 0.85]**
Heterogeneity: $Tau^2 = 0$; $Chi^2 = 0.15$, df = 1 (P = .698); $I^2 = 0\%$						
Test for overall effect: Z = −3.09 (P = .002)						
**Total (95% CI)**		**398**		**365**	**100.0%**	**0.60 [0.47, 0.77]**
Heterogeneity: $Tau^2 = 0.007$; $Chi^2 = 8.31$, df = 8 (P = .404); $I^2 = 4\%$						
Test for overall effect: Z = −3.97 (P < .001)						
Test for subgroup differences: $Chi^2 = 4.14$, df = 3 (P = .247)						

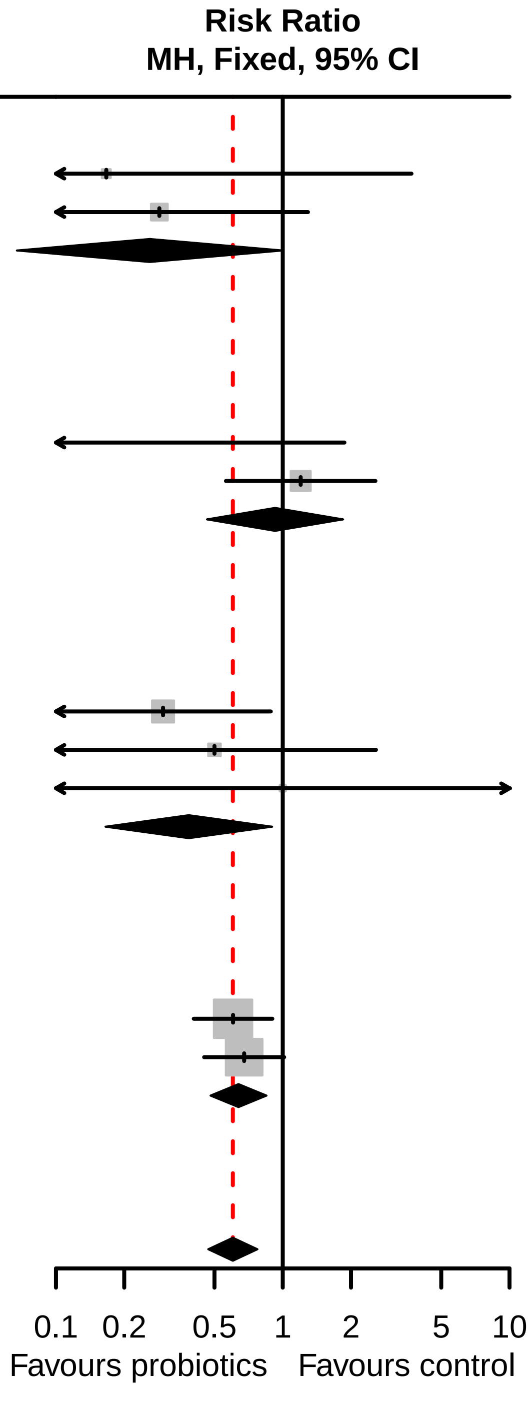

Figure S4. The forest plot of overt HE incidence tested in different follow-up time-point

Study or Subgroup	Probiotics Events	Probiotics Total	Control Events	Control Total	Weight	Risk Ratio MH, Fixed, 95% CI
**less than 3 months**						
Shavakhi A, 2014	1	19	1	21	2.7%	1.11 [0.07, 16.47]
Mittal V V, 2011	1	40	2	40	5.7%	0.50 [0.05, 5.30]
**Total (95% CI)**		**59**		**61**	**8.3%**	**0.69 [0.12, 3.98]**
Heterogeneity: $Tau^2 = 0$; $Chi^2 = 0.19$, df = 1 (P = .665); $I^2 = 0\%$						
Test for overall effect: Z = −0.41 (P = .683)						
**6 months**						
Dhiman R K, 2014	16	66	29	64	83.2%	0.54 [0.32, 0.89]
Pereg D, 2011	3	20	3	20	8.5%	1.00 [0.23, 4.37]
**Total (95% CI)**		**86**		**84**	**91.7%**	**0.58 [0.36, 0.93]**
Heterogeneity: $Tau^2 = 0$; $Chi^2 = 0.62$, df = 1 (P = .431); $I^2 = 0\%$						
Test for overall effect: Z = −2.26 (P = .024)						
**Total (95% CI)**		**145**		**145**	**100.0%**	**0.59 [0.37, 0.93]**
Heterogeneity: $Tau^2 = 0$; $Chi^2 = 0.86$, df = 3 (P = .835); $I^2 = 0\%$						
Test for overall effect: Z = −2.26 (P = .024)						
Test for subgroup differences: $Chi^2 = 0.04$, df = 1 (P = .842)						

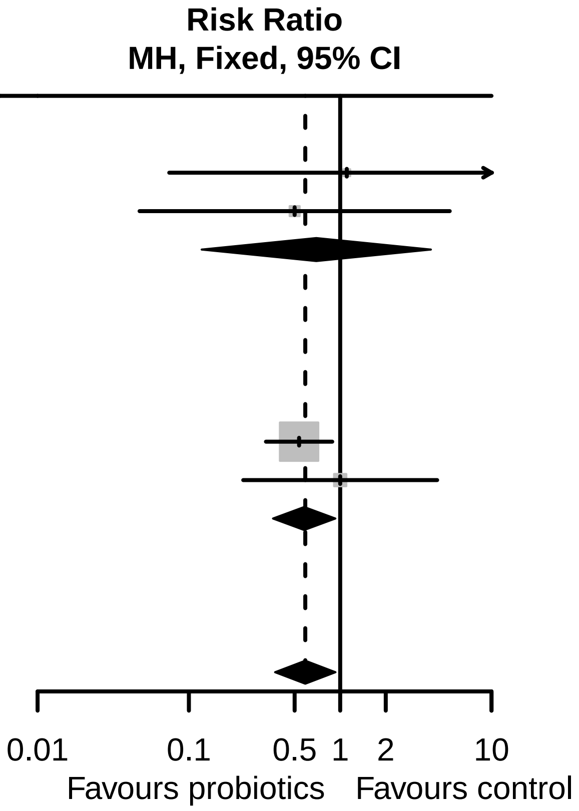

Figure S5. The forest plot of hospitalization incidence tested in different follow-up time-point

Study or Subgroup	Probiotics Events	Probiotics Total	Control Events	Control Total	Weight	Risk Ratio MH, Fixed, 95% CI
**less than 3 months**						
Macnaughtan J, 2020	0	33	1	41	2.7%	0.41 [0.02, 9.79]
Bajaj J S, 2014	1	14	1	16	1.9%	1.14 [0.08, 16.63]
Roman E, 2019	4	18	5	18	10.0%	0.80 [0.26, 2.50]
**Total (95% CI)**		**65**		**75**	**14.5%**	**0.77 [0.29, 2.09]**
Heterogeneity: $Tau^2 = 0$; $Chi^2 = 0.24$, df = 2 (P = .888); $I^2 = 0\%$						
Test for overall effect: Z = −0.51 (P = .612)						
**more than 6 months**						
Horvath A, 2016	15	44	28	36	61.4%	0.44 [0.28, 0.68]
Horvath A, 2016	6	44	11	36	24.1%	0.45 [0.18, 1.09]
**Total (95% CI)**		**88**		**72**	**85.5%**	**0.44 [0.29, 0.66]**
Heterogeneity: $Tau^2 = 0$; $Chi^2 = 0$, df = 1 (P = .971); $I^2 = 0\%$						
Test for overall effect: Z = −3.94 (P < .001)						
**Total (95% CI)**		**153**		**147**	**100.0%**	**0.49 [0.33, 0.71]**
Heterogeneity: $Tau^2 = 0$; $Chi^2 = 1.38$, df = 4 (P = .847); $I^2 = 0\%$						
Test for overall effect: Z = −3.72 (P < .001)						
Test for subgroup differences: $Chi^2 = 1.04$, df = 1 (P = .307)						

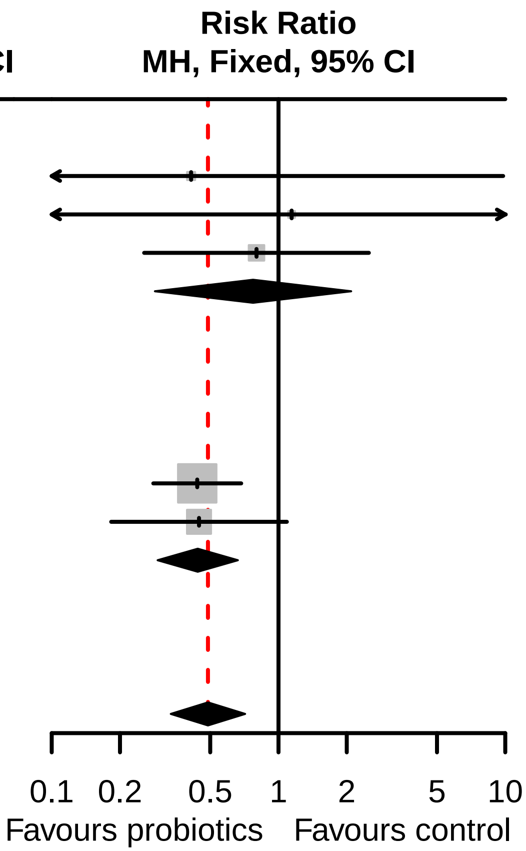

Figure S6. The forest plot of infections incidence tested in different follow-up time-point

Study	Probiotics Events	Probiotics Total	Control Events	Control Total	Weight	Risk Ratio MH, Fixed, 95% CI	Risk Ratio MH, Fixed, 95% CI
Bajaj J S, 2008	3	17	0	8	2.7%	3.50 [0.20, 60.70]	
Dhiman R K, 2014	3	66	3	64	12.3%	0.97 [0.20, 4.63]	
Mittal V V, 2011	3	40	3	40	12.2%	1.00 [0.21, 4.66]	
Pande C, 2012	10	55	12	55	48.6%	0.83 [0.39, 1.77]	
Pereg D, 2011	2	20	2	20	8.1%	1.00 [0.16, 6.42]	
Pratap Mouli V, 2015	2	60	2	60	8.1%	1.00 [0.15, 6.87]	
Ramachandran G, 2023	2	108	1	107	4.1%	1.98 [0.18, 21.53]	
Shavakhi A, 2014	1	19	1	21	3.9%	1.11 [0.07, 16.47]	
**Total (95% CI)**		**385**		**375**	**100.0%**	**1.03 [0.61, 1.73]**	

Heterogeneity: $Tau^2 = 0$; $Chi^2 = 1.31$, df = 7 (P = .988); $I^2 = 0\%$
Test for overall effect: Z = 0.10 (P = .921)

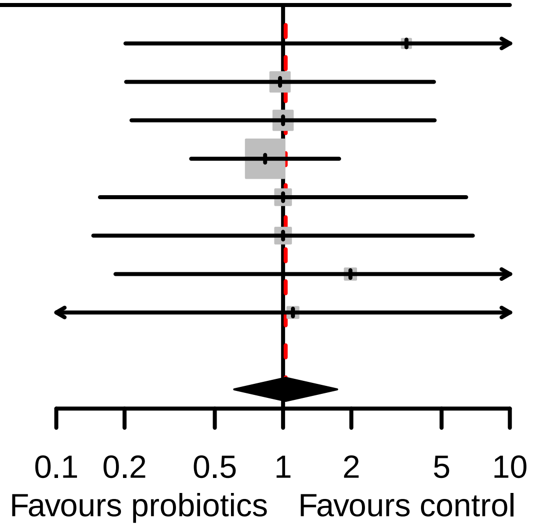

Figure S7. The forest plot of the nonadherence rate

Study or Subgroup	Probiotics Mean	Probiotics SD	Probiotics Total	Control Mean	Control SD	Control Total	Weight	Std. Mean Difference IV, Random, 95% CI
**INR**								
Pereg D, 2011	0.00	0.08	18	0.00	0.14	18	10.1%	0.00 [−0.65, 0.65]
Ramachandran G, 2023	0.04	0.09	93	0.06	0.11	90	12.9%	−0.20 [−0.49, 0.09]
Roman E, 2019	−0.05	0.08	17	0.01	0.06	18	9.8%	−0.83 [−1.53, −0.14]
**Total (95% CI)**			**128**			**126**	**32.8%**	**−0.29 [−0.68, 0.10]**
Heterogeneity: Tau^2 = 0.051; Chi^2 = 3.39, df = 2 (P = .184); I^2 = 41%								
Test for overall effect: Z = −1.47 (P = .142)								
**Creatinine**								
Pereg D, 2011	0.00	0.50	18	−0.10	0.36	18	10.1%	0.22 [−0.43, 0.88]
Ramachandran G, 2023	0.01	0.08	93	0.07	0.09	90	12.8%	−0.70 [−1.00, −0.40]
Roman E, 2019	1.00	8.48	17	−0.80	9.06	18	10.0%	0.20 [−0.46, 0.86]
**Total (95% CI)**			**128**			**126**	**32.9%**	**−0.14 [−0.85, 0.56]**
Heterogeneity: Tau^2 = 0.307; Chi^2 = 10.47, df = 2 (P = .005); I^2 = 81%								
Test for overall effect: Z = −0.40 (P = .689)								
**TBIL**								
Pereg D, 2011	−0.10	0.64	18	−0.20	0.85	18	10.1%	0.13 [−0.52, 0.78]
Ramachandran G, 2023	−0.17	0.36	93	−0.52	0.53	90	12.8%	0.77 [0.47, 1.07]
Sharma P, 2008	−0.30	1.42	31	−0.70	1.34	31	11.4%	0.29 [−0.21, 0.79]
**Total (95% CI)**			**142**			**139**	**34.3%**	**0.47 [0.05, 0.88]**
Heterogeneity: Tau^2 = 0.077; Chi^2 = 4.7, df = 2 (P = .095); I^2 = 57%								
Test for overall effect: Z = 2.20 (P = .028)								
**Total (95% CI)**			**398**			**391**	**100.0%**	**−0.01 [−0.41, 0.39]**
Heterogeneity: Tau^2 = 0.307; Chi^2 = 55.99, df = 8 (P < .001); I^2 = 86%								
Test for overall effect: Z = −0.05 (P = .962)								
Test for subgroup differences: Chi^2 = 7.09, df = 2 (P = .029)								

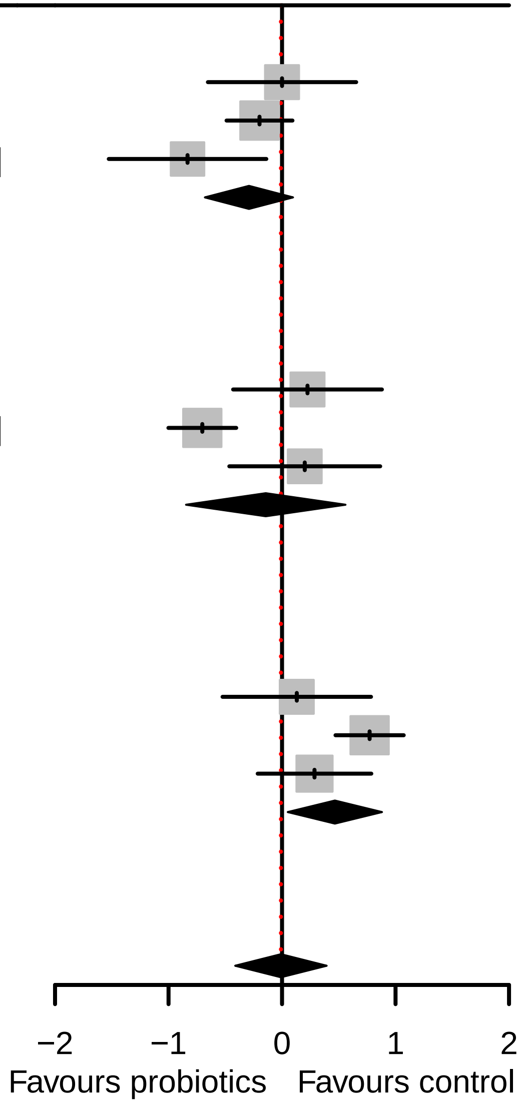

Figure S8. The forest plots of the effect of probiotics in the measures of MELD

Study or Subgroup	Probiotics Mean	Probiotics SD	Probiotics Total	Control Mean	Control SD	Control Total	Weight	Std. Mean Difference IV, Fixed, 95% CI
**Serum sodium**								
Ramachandran G, 2023	−0.17	0.70	93	0.41	0.69	90	82.9%	−0.83 [−1.13, −0.53]
Roman E, 2019	0.20	1.13	17	0.50	1.06	18	17.1%	−0.27 [−0.93, 0.40]
**Total (95% CI)**			**110**			**108**	**100.0%**	**−0.73 [−1.01, −0.46]**
Heterogeneity: Tau^2 = 0.089; Chi^2 = 2.28, df = 1 (P = .131); I^2 = 56%								
Test for overall effect: Z = −5.23 (P < .001)								
**Total (95% CI)**			**110**			**108**	**100.0%**	**−0.73 [−1.01, −0.46]**
Heterogeneity: Tau^2 = 0.089; Chi^2 = 2.28, df = 1 (P = .131); I^2 = 56%								
Test for overall effect: Z = −5.23 (P < .001)								
Test for subgroup differences: Chi^2 = 0.00, df = 0 (P = NA)								

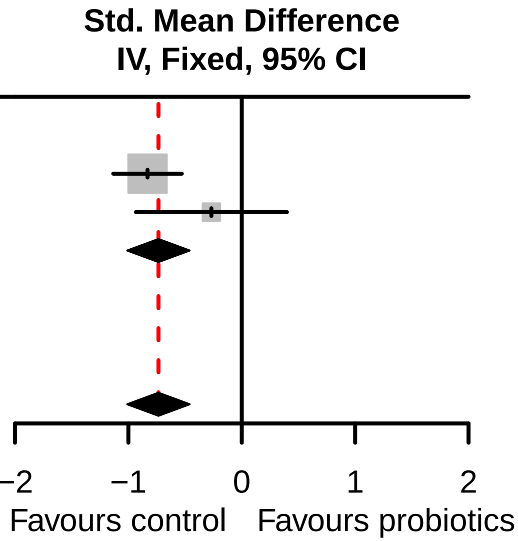

Figure S9. The forest plot of the serum sodium levels

Study or Subgroup	Probiotics Mean	Probiotics SD	Probiotics Total	Control Mean	Control SD	Control Total	Weight	Std. Mean Difference IV, Random, 95% CI
**ALT**								
Pereg D, 2011	−0.60	43.83	18	6.40	46.38	18	5.0%	−0.15 [−0.81, 0.50]
Ramachandran G, 2023	−1.64	2.30	93	−2.30	2.52	90	6.4%	0.27 [−0.02, 0.56]
Roman E, 2019	5.60	12.33	17	4.90	8.26	18	5.0%	0.07 [−0.60, 0.73]
Sharma P, 2008	−16.60	26.16	31	−8.60	23.00	31	5.7%	−0.32 [−0.82, 0.18]
Shi J, 2023	−39.27	13.80	44	−21.86	16.08	44	5.9%	−1.15 [−1.60, −0.70]
**Total (95% CI)**			**203**			**201**	**28.0%**	**−0.26 [−0.83, 0.31]**
Heterogeneity: $Tau^2 = 0.348$; $Chi^2 = 27.76$, df = 4 (P < .001); $I^2 = 86\%$								
Test for overall effect: Z = −0.90 (P = .370)								
**AST**								
Pereg D, 2011	−4.00	40.40	18	4.20	43.60	18	5.0%	−0.19 [−0.85, 0.46]
Ramachandran G, 2023	−4.88	6.20	93	−9.94	5.70	90	6.4%	0.85 [0.54, 1.15]
Roman E, 2019	−1.70	7.85	17	2.60	6.79	18	4.9%	−0.57 [−1.25, 0.10]
Sharma P, 2008	−24.70	45.12	31	−20.50	27.42	31	5.7%	−0.11 [−0.61, 0.39]
Shi J, 2023	−43.09	27.58	44	−24.73	19.66	44	5.9%	−0.76 [−1.19, −0.33]
**Total (95% CI)**			**203**			**201**	**27.9%**	**−0.14 [−0.85, 0.57]**
Heterogeneity: $Tau^2 = 0.581$; $Chi^2 = 43.62$, df = 4 (P < .001); $I^2 = 91\%$								
Test for overall effect: Z = −0.39 (P = .698)								
**GGT**								
Ramachandran G, 2023	−7.01	13.22	93	−15.93	10.49	90	6.4%	0.74 [0.44, 1.04]
Roman E, 2019	−13.20	18.58	17	17.30	27.42	18	4.7%	−1.27 [−2.00, −0.53]
**Total (95% CI)**			**110**			**108**	**11.1%**	**−0.23 [−2.20, 1.74]**
Heterogeneity: $Tau^2 = 1.935$; $Chi^2 = 24.68$, df = 1 (P < .001); $I^2 = 96\%$								
Test for overall effect: Z = −0.23 (P = .817)								
**ALB**								
Pereg D, 2011	0.10	0.78	18	−0.10	0.78	18	5.0%	0.25 [−0.41, 0.91]
Ramachandran G, 2023	0.14	0.87	93	0.03	0.13	90	6.4%	0.17 [−0.12, 0.47]
Roman E, 2019	0.20	1.06	17	0.50	1.91	18	5.0%	−0.19 [−0.85, 0.48]
Sharma P, 2008	0.10	0.71	31	0.20	0.71	31	5.7%	−0.14 [−0.64, 0.36]
Ziada DH, 2013a	0.05	0.59	26	0.03	50.00	24	5.4%	0.00 [−0.55, 0.56]
Ziada DH, 2013b	0.05	0.59	26	−0.04	0.43	25	5.5%	0.17 [−0.38, 0.72]
**Total (95% CI)**			**211**			**206**	**33.0%**	**0.08 [−0.11, 0.28]**
Heterogeneity: $Tau^2 = 0$; $Chi^2 = 2.22$, df = 5 (P = .818); $I^2 = 0\%$								
Test for overall effect: Z = 0.84 (P = .399)								
**Total (95% CI)**			**727**			**716**	**100.0%**	**−0.10 [−0.38, 0.18]**
Heterogeneity: $Tau^2 = 0.296$; $Chi^2 = 110.79$, df = 17 (P < .001); $I^2 = 85\%$								
Test for overall effect: Z = −0.72 (P = .470)								
Test for subgroup differences: $Chi^2 = 1.58$, df = 3 (P = .665)								

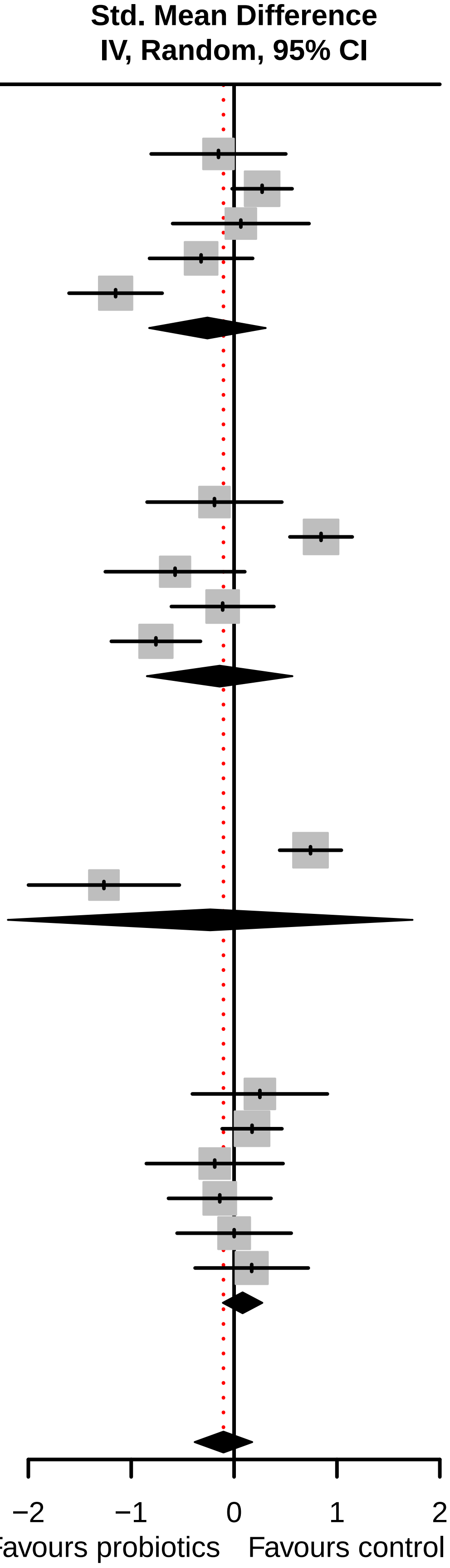

Figure S10. The forest plot of the liver function parameter

Study or Subgroup	Probiotics Events	Probiotics Total	Control Events	Control Total	Weight	Risk Ratio MH, Random, 95% CI
**Child–Pugh class A**						
Bajaj J S, 2008	14	14	5	6	27.1%	1.18 [0.85, 1.64]
Efremova I, 2024	8	20	0	13	2.1%	11.20 [0.70, 178.34]
Horvath A, 2016	24	44	33	36	28.2%	0.60 [0.45, 0.79]
Liu Q, 2004	6	17	4	17	10.2%	1.50 [0.51, 4.38]
Maslennikov R, 2022	8	24	1	16	3.8%	5.33 [0.74, 38.64]
Sharma P, 2008	17	31	18	31	23.9%	0.94 [0.61, 1.46]
**Total (95% CI)**		**150**		**119**	**95.3%**	**1.06 [0.68, 1.66]**
Heterogeneity: $Tau^2 = 0.159$; $Chi^2 = 17.7$, df = 5 (P = .003); $I^2 = 72\%$						
Test for overall effect: Z = 0.27 (P = .789)						
**Child–Pugh class B**						
Bajaj J S, 2008	0	14	1	6	1.7%	0.15 [0.01, 3.20]
Liu Q, 2004	2	17	1	13	3.0%	1.53 [0.15, 15.09]
**Total (95% CI)**		**31**		**19**	**4.7%**	**0.60 [0.06, 5.63]**
Heterogeneity: $Tau^2 = 0.801$; $Chi^2 = 1.42$, df = 1 (P = .233); $I^2 = 30\%$						
Test for overall effect: Z = −0.44 (P = .657)						
**Total (95% CI)**		**181**		**138**	**100.0%**	**1.02 [0.68, 1.54]**
Heterogeneity: $Tau^2 = 0.136$; $Chi^2 = 19.20$, df = 7 (P = .008); $I^2 = 64\%$						
Test for overall effect: Z = 0.10 (P = .922)						
Test for subgroup differences: $Chi^2 = 0.24$, df = 1 (P = .626)						

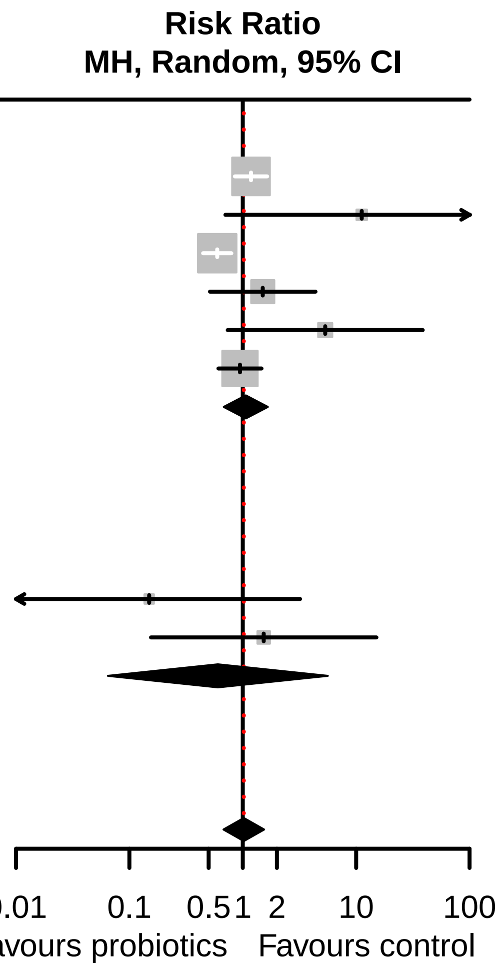

Figure S11. The forest plot of the Child-Turcotte-Pugh classification

Figure S12a. The forest plot of the level of ALT tested at different treatment time points

Study or Subgroup	Probiotics Mean	Probiotics SD	Probiotics Total	Control Mean	Control SD	Control Total	Weight	Std. Mean Difference IV, Random, 95% CI
**less than 2 months**								
Pereg D, 2011	−0.90	49.76	18	8.70	56.70	18	13.5%	−0.18 [−0.83, 0.48]
Ramachandran G, 2023	−1.64	2.30	93	−2.30	2.52	90	16.5%	0.27 [−0.02, 0.56]
Sharma P, 2008	−16.60	26.16	31	−8.60	23.00	31	14.9%	−0.32 [−0.82, 0.18]
Shi J, 2023	−39.27	13.80	44	−21.86	16.08	44	15.3%	−1.15 [−1.60, −0.70]
**Total (95% CI)**			**186**			**183**	**60.3%**	**−0.34 [−1.02, 0.34]**
Heterogeneity: $Tau^2 = 0.422$; $Chi^2 = 27.29$, df = 3 (P < .001); $I^2 = 89\%$								
Test for overall effect: Z = −0.97 (P = .331)								
**3 months**								
Pereg D, 2011	−3.00	45.68	18	45.85	0.36	18	12.7%	−1.48 [−2.22, −0.73]
Roman E, 2019	5.60	12.33	17	4.90	8.26	18	13.5%	0.07 [−0.60, 0.73]
**Total (95% CI)**			**35**			**36**	**26.1%**	**−0.70 [−2.21, 0.82]**
Heterogeneity: $Tau^2 = 1.063$; $Chi^2 = 9.19$, df = 1 (P = .002); $I^2 = 89\%$								
Test for overall effect: Z = −0.90 (P = .367)								
**6 months**								
Pereg D, 2011	−0.60	43.83	18	6.40	46.38	18	13.5%	−0.15 [−0.81, 0.50]
Test for overall effect: Z = −0.45 (P = .650)								
**Total (95% CI)**			**239**			**237**	**100.0%**	**−0.40 [−0.91, 0.10]**

Heterogeneity: $Tau^2 = 0.380$; $Chi^2 = 39.10$, df = 6 (P < .001); $I^2 = 85\%$

Test for overall effect: Z = −1.56 (P = .119)

Test for subgroup differences: $Chi^2 = 0.47$, df = 2 (P = .792)

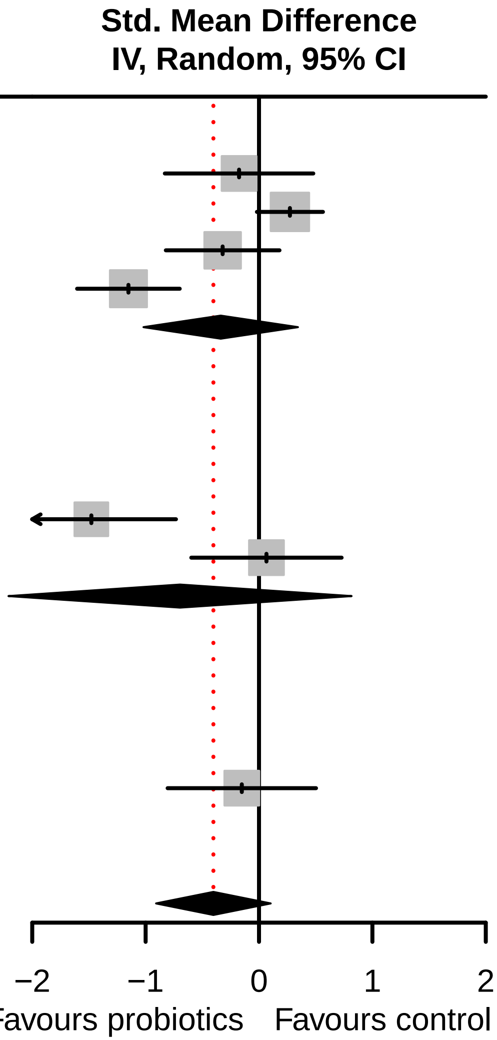

Figure S12b. The forest plot of the level of AST tested at different treatment time points

Study or Subgroup	Probiotics Mean	Probiotics SD	Probiotics Total	Control Mean	Control SD	Control Total	Weight	Std. Mean Difference IV, Random, 95% CI
**less than 2 months**								
Pereg D, 2011	−2.00	36.27	18	5.00	48.30	18	13.5%	−0.16 [−0.81, 0.49]
Ramachandran G, 2023	−4.88	6.20	93	−9.94	5.70	90	16.1%	0.85 [0.54, 1.15]
Sharma P, 2008	−24.70	45.12	31	−20.50	27.42	31	14.8%	−0.11 [−0.61, 0.39]
Shi J, 2023	−43.09	27.58	44	−24.73	19.66	44	15.3%	−0.76 [−1.19, −0.33]
**Total (95% CI)**			**186**			**183**	**59.6%**	**−0.03 [−0.84, 0.77]**
Heterogeneity: $Tau^2 = 0.619$; $Chi^2 = 38.97$, df = 3 (P < .001); $I^2 = 92\%$								
Test for overall effect: Z = −0.08 (P = .933)								
**3 months**								
Pereg D, 2011	−4.10	36.91	18	8.20	50.50	18	13.5%	−0.27 [−0.93, 0.38]
Roman E, 2019	−1.70	7.85	17	2.60	6.79	18	13.3%	−0.57 [−1.25, 0.10]
**Total (95% CI)**			**35**			**36**	**26.8%**	**−0.42 [−0.89, 0.05]**
Heterogeneity: $Tau^2 = 0$; $Chi^2 = 0.39$, df = 1 (P = .531); $I^2 = 0\%$								
Test for overall effect: Z = −1.74 (P = .082)								
**6 months**								
Pereg D, 2011	−4.00	40.40	18	4.20	43.60	18	13.5%	−0.19 [−0.85, 0.46]
Test for overall effect: Z = −0.57 (P = .568)								
**Total (95% CI)**			**239**			**237**	**100.0%**	**−0.16 [−0.69, 0.38]**
Heterogeneity: $Tau^2 = 0.443$; $Chi^2 = 45.40$, df = 6 (P < .001); $I^2 = 87\%$								
Test for overall effect: Z = −0.57 (P = .566)								
Test for subgroup differences: $Chi^2 = 0.75$, df = 2 (P = .686)								

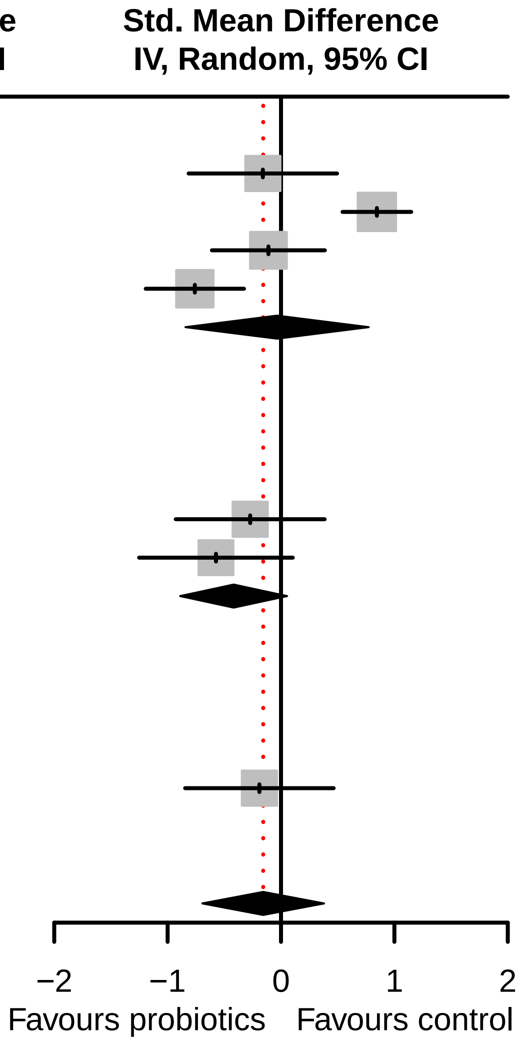

Figure S12c. The forest plot of the level of ALB tested at different treatment time points

Study or Subgroup	Probiotics Mean	Probiotics SD	Probiotics Total	Control Mean	Control SD	Control Total	Weight	Std. Mean Difference IV, Fixed, 95% CI
**less than 2 months**								
Pereg D, 2011	0.15	0.78	18	0.10	0.85	18	7.4%	0.06 [−0.59, 0.71]
Ramachandran G, 2023	0.14	0.87	93	0.03	0.13	90	37.4%	0.17 [−0.12, 0.47]
Sharma P, 2008	0.10	0.71	31	0.20	0.71	31	12.7%	−0.14 [−0.64, 0.36]
Ziada DH, 2013a	0.05	0.59	26	0.03	0.50	24	10.2%	0.04 [−0.52, 0.59]
Ziada DH, 2013b	0.05	0.59	26	−0.04	0.43	25	10.4%	0.17 [−0.38, 0.72]
**Total (95% CI)**			**194**			**188**	**78.1%**	**0.09 [−0.11, 0.30]**
Heterogeneity: $Tau^2 = 0$; $Chi^2 = 1.26$, df = 4 (P = .867); $I^2 = 0\%$								
Test for overall effect: Z = 0.92 (P = .358)								
**3 months**								
Pereg D, 2011	0.10	0.78	18	0.10	0.85	18	7.4%	0.00 [−0.65, 0.65]
Roman E, 2019	0.20	1.06	17	0.50	1.91	18	7.1%	−0.19 [−0.85, 0.48]
**Total (95% CI)**			**35**			**36**	**14.5%**	**−0.09 [−0.56, 0.37]**
Heterogeneity: $Tau^2 = 0$; $Chi^2 = 0.16$, df = 1 (P = .692); $I^2 = 0\%$								
Test for overall effect: Z = −0.39 (P = .697)								
**6 months**								
Pereg D, 2011	0.10	0.78	18	−0.10	0.78	18	7.3%	0.25 [−0.41, 0.91]
Test for overall effect: Z = 0.75 (P = .454)								
**Total (95% CI)**			**247**			**242**	**100.0%**	**0.08 [−0.10, 0.26]**
Heterogeneity: $Tau^2 = 0$; $Chi^2 = 2.23$, df = 7 (P = .946); $I^2 = 0\%$								
Test for overall effect: Z = 0.87 (P = .386)								
Test for subgroup differences: $Chi^2 = 0.81$, df = 2 (P = .668)								

Std. Mean Difference IV, Fixed, 95% CI

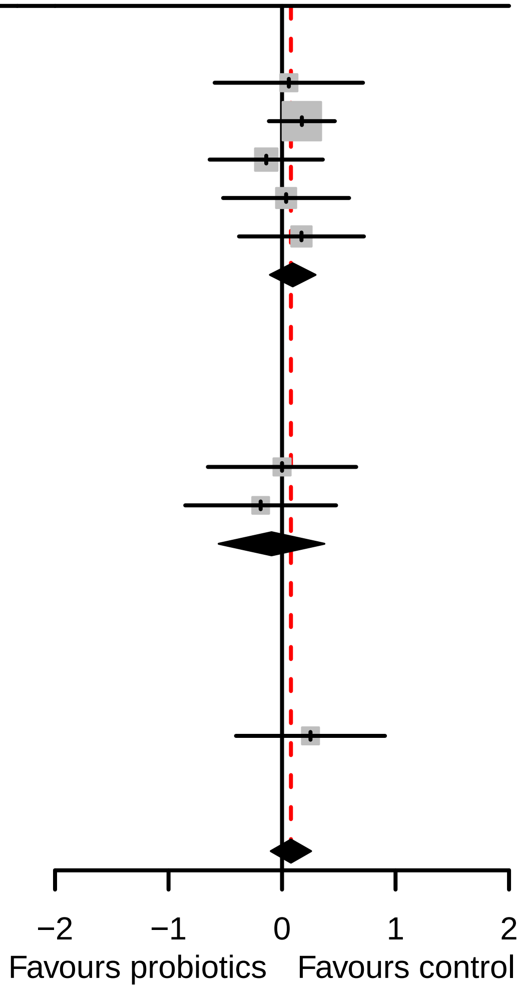

Figure S12d. The forest plot of the level of INR tested at different treatment time points

Study or Subgroup	Probiotics Mean	Probiotics SD	Probiotics Total	Control Mean	Control SD	Control Total	Weight	Std. Mean Difference IV, Fixed, 95% CI
**less than 2 months**								
Pereg D, 2011	0.00	0.13	18	0.00	0.17	18	11.2%	0.00 [−0.65, 0.65]
Ramachandran G, 2023	0.04	0.09	93	0.06	0.11	90	56.5%	−0.20 [−0.49, 0.09]
**Total (95% CI)**			**111**			**108**	**67.7%**	**−0.17 [−0.43, 0.10]**
Heterogeneity: $Tau^2 = 0$; $Chi^2 = 0.3$, df = 1 (P = .586); $I^2 = 0\%$								
Test for overall effect: Z = −1.22 (P = .221)								
**3 months**								
Pereg D, 2011	0.00	0.12	18	0.00	0.16	18	11.2%	0.00 [−0.65, 0.65]
Roman E, 2019	−0.05	0.08	17	0.01	0.06	18	9.9%	−0.83 [−1.53, −0.14]
**Total (95% CI)**			**35**			**36**	**21.1%**	**−0.39 [−0.87, 0.08]**
Heterogeneity: $Tau^2 = 0.228$; $Chi^2 = 2.93$, df = 1 (P = .087); $I^2 = 66\%$								
Test for overall effect: Z = −1.61 (P = .107)								
**6 months**								
Pereg D, 2011	0.00	0.08	18	0.00	0.14	18	11.2%	0.00 [−0.65, 0.65]
Test for overall effect: Z = 0.00 (P = 1.000)								
**Total (95% CI)**			**164**			**162**	**100.0%**	**−0.19 [−0.41, 0.02]**

Heterogeneity: $Tau^2 = 0.005$; $Chi^2 = 4.27$, df = 4 (P = .371); $I^2 = 6\%$
Test for overall effect: Z = −1.75 (P = .081)
Test for subgroup differences: $Chi^2 = 1.04$, df = 2 (P = .594)

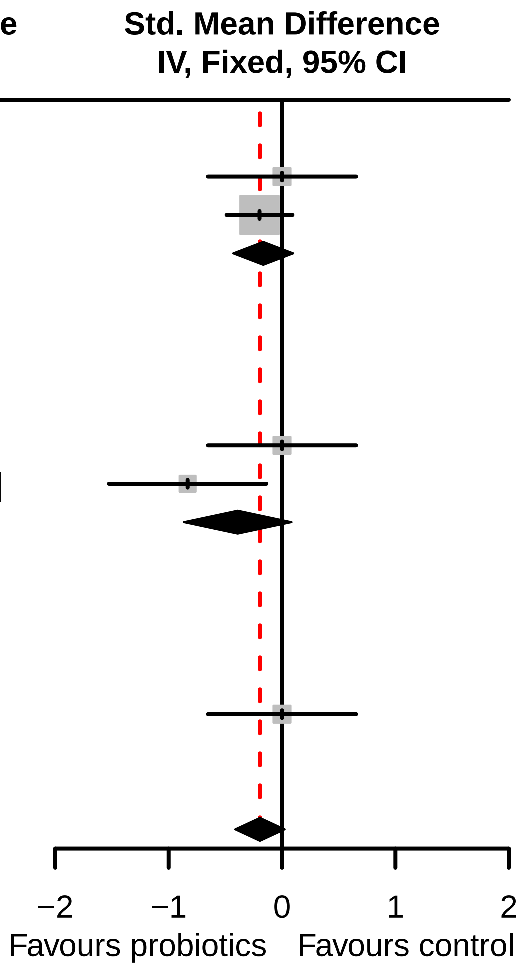

Figure S12e. The forest plot of the level of creatinine tested at different treatment time points

Study or Subgroup	Probiotics Mean	Probiotics SD	Probiotics Total	Control Mean	Control SD	Control Total	Weight	Std. Mean Difference IV, Random, 95% CI
**less than 2 months**								
Pereg D, 2011	0.00	0.57	18	0.00	0.50	18	18.7%	0.00 [−0.65, 0.65]
Ramachandran G, 2023	0.01	0.08	93	0.07	0.09	90	25.7%	−0.70 [−1.00, −0.40]
**Total (95% CI)**			**111**			**108**	**44.4%**	**−0.41 [−1.09, 0.26]**
Heterogeneity: $Tau^2 = 0.180$; $Chi^2 = 3.67$, df = 1 (P = .055); $I^2 = 73\%$								
Test for overall effect: Z = −1.20 (P = .231)								
**3 months**								
Pereg D, 2011	0.00	0.57	18	−0.10	0.36	18	18.6%	0.21 [−0.45, 0.86]
Roman E, 2019	1.00	8.48	17	−0.80	9.06	18	18.4%	0.20 [−0.46, 0.86]
**Total (95% CI)**			**35**			**36**	**37.0%**	**0.20 [−0.26, 0.67]**
Heterogeneity: $Tau^2 = 0$; $Chi^2 = 0$, df = 1 (P = .992); $I^2 = 0\%$								
Test for overall effect: Z = 0.85 (P = .395)								
**6 months**								
Pereg D, 2011	0.00	0.50	18	−0.10	0.36	18	18.6%	0.22 [−0.43, 0.88]
Test for overall effect: Z = 0.67 (P = .502)								
**Total (95% CI)**			**164**			**162**	**100.0%**	**−0.06 [−0.54, 0.42]**
Heterogeneity: $Tau^2 = 0.209$; $Chi^2 = 14.68$, df = 4 (P = .005); $I^2 = 73\%$								
Test for overall effect: Z = −0.26 (P = .794)								
Test for subgroup differences: $Chi^2 = 2.48$, df = 2 (P = .289)								

Std. Mean Difference
IV, Random, 95% CI

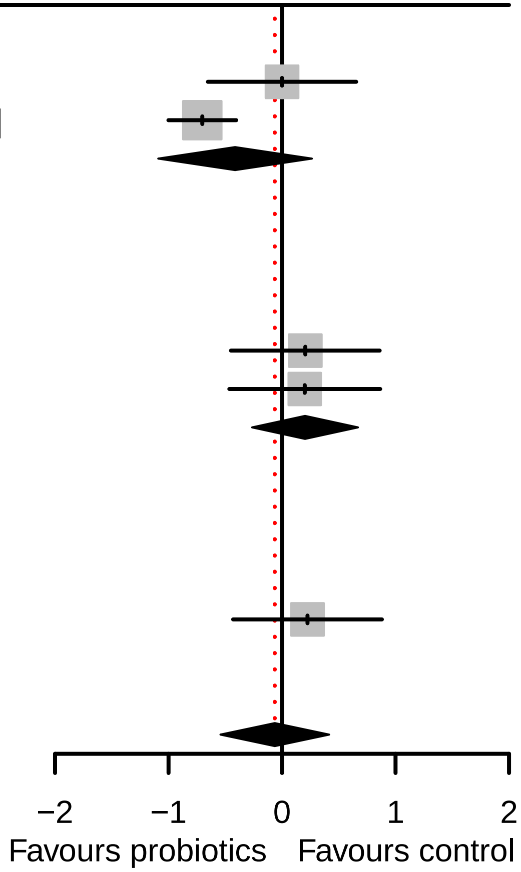

Figure S12f. The forest plot of the level of TBIL tested at different treatment time points

Study or Subgroup	Probiotics Mean	Probiotics SD	Probiotics Total	Control Mean	Control SD	Control Total	Weight	Std. Mean Difference IV, Random, 95% CI
**less than 2 months**								
Pereg D, 2011	0.00	0.78	18	0.10	1.00	18	16.6%	−0.11 [−0.76, 0.54]
Ramachandran G, 2023	−0.17	0.36	93	−0.52	0.53	90	28.9%	0.77 [0.47, 1.07]
Sharma P, 2008	−0.30	1.42	31	−0.70	1.34	31	21.4%	0.29 [−0.21, 0.79]
**Total (95% CI)**			**142**			**139**	**66.8%**	**0.38 [−0.13, 0.89]**
Heterogeneity: Tau^2 = 0.144; Chi^2 = 7.06, df = 2 (P = .029); I^2 = 72%								
Test for overall effect: Z = 1.45 (P = .148)								
**3 months**								
Roman E, 2019	−0.10	0.71	18	−0.20	0.85	18	16.6%	0.12 [−0.53, 0.78]
Test for overall effect: Z = 0.37 (P = .708)								
**6 months**								
Pereg D, 2011	−0.10	0.64	18	−0.20	0.85	18	16.6%	0.13 [−0.52, 0.78]
Test for overall effect: Z = 0.39 (P = .697)								
**Total (95% CI)**			**178**			**175**	**100.0%**	**0.31 [−0.05, 0.67]**
Heterogeneity: Tau^2 = 0.095; Chi^2 = 9.49, df = 4 (P = .050); I^2 = 58%								
Test for overall effect: Z = 1.67 (P = .096)								
Test for subgroup differences: Chi^2 = 0.51, df = 2 (P = .776)								

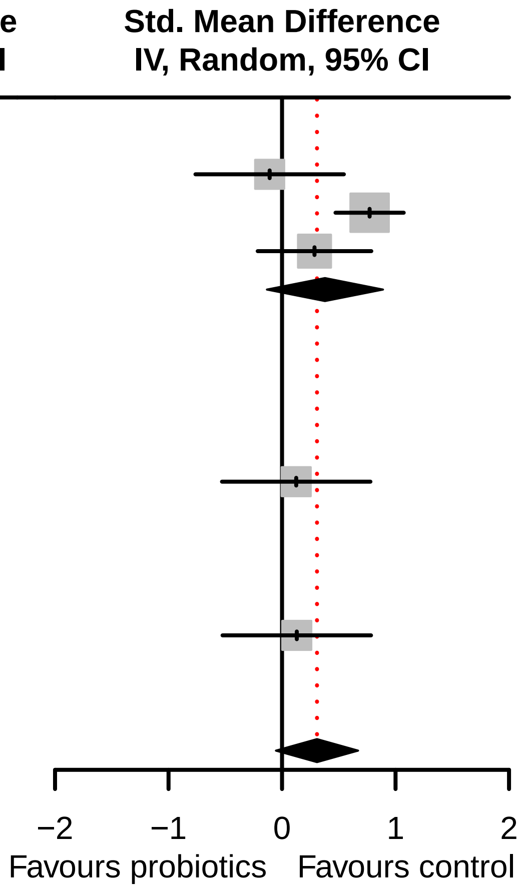

Figure S12. The forest plot of the level of liver function parameters tested at different treatment time points

Figure S13a. Sensitivity analysis of creatinine

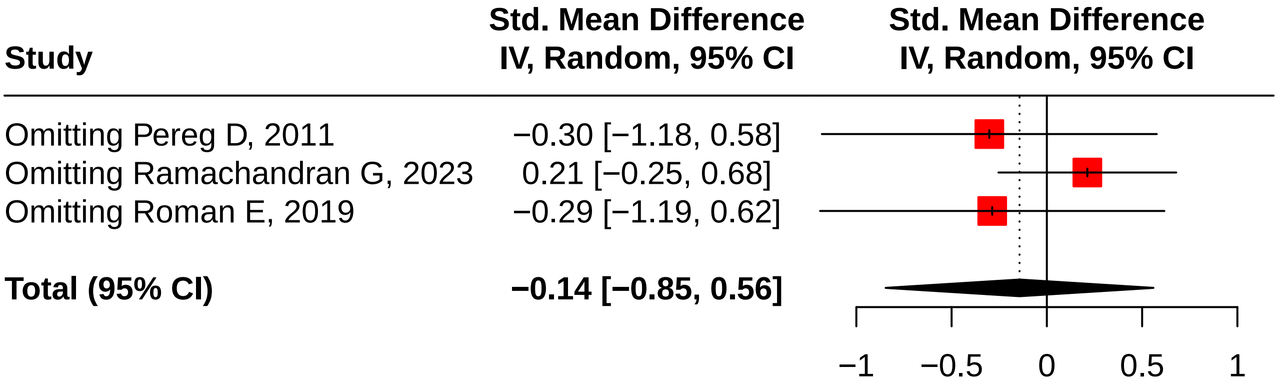

Figure S13b. Sensitivity analysis of INR

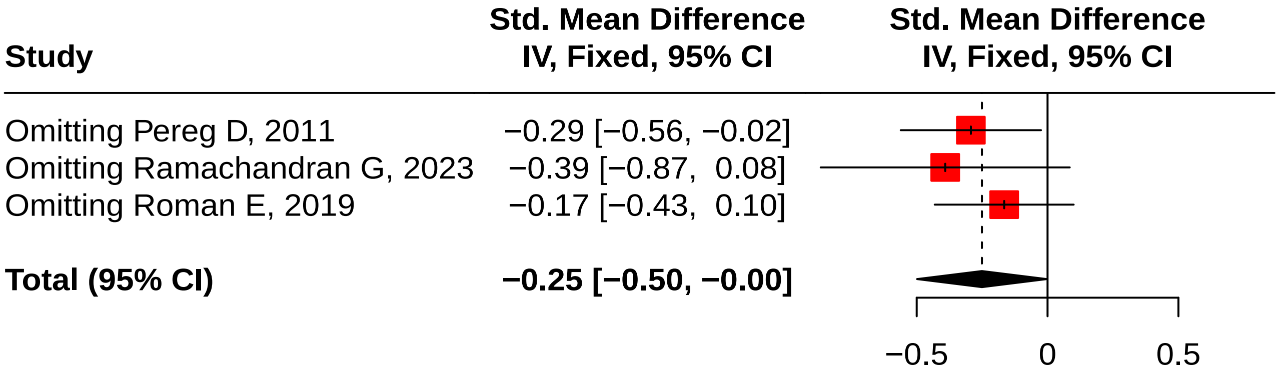

Figure S13. Sensitivity analysis of liver function parameters

Study	Std. Mean Difference IV, Random, 95% CI	Std. Mean Difference IV, Random, 95% CI
Omitting Macnaughtan J, 2020	0.55 [0.04, 1.05]	
Omitting Ramachandran G, 2023	0.14 [−0.25, 0.53]	
Omitting Roman E, 2019	0.45 [−0.15, 1.05]	
**Total (95% CI)**	**0.40 [−0.05, 0.84]**	

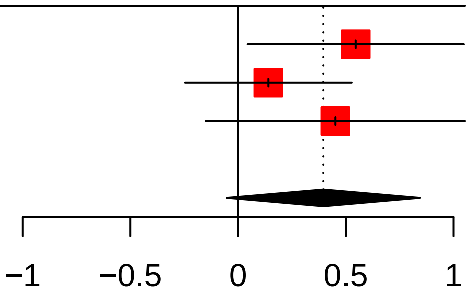

Figure S14. Sensitivity analysis of quality of life

Figure S15a. Sensitivity analysis of *Bacteroidaceae*

Study	Std. Mean Difference IV, Random, 95% CI
Omitting Koga H, 2013	0.61 [−0.60, 1.81]
Omitting Ziada DH, 2013a	0.62 [−0.58, 1.82]
Omitting Ziada DH, 2013b	0.00 [−0.42, 0.42]
**Total (95% CI)**	**0.41 [−0.39, 1.21]**

Std. Mean Difference IV, Random, 95% CI

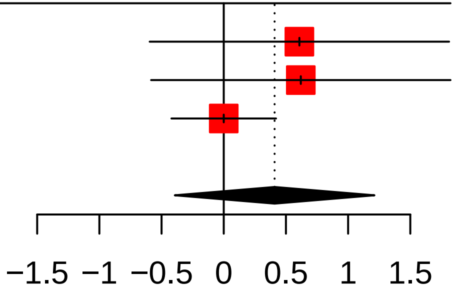

Figure S15b. Sensitivity analysis of *Enterococcus*

Study	Std. Mean Difference IV, Random, 95% CI
Omitting Liu Q, 2004	−0.67 [−1.65, 0.30]
Omitting Ziada DH, 2013a	−0.47 [−1.87, 0.93]
Omitting Ziada DH, 2013b	0.01 [−0.41, 0.42]
**Total (95% CI)**	**−0.37 [−1.18, 0.44]**

Std. Mean Difference IV, Random, 95% CI

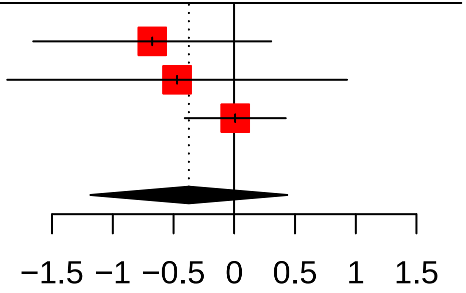

Figure S15. Sensitivity analysis of gut microbiota

Study or Subgroup	Probiotics Mean	Probiotics SD	Probiotics Total	Control Mean	Control SD	Control Total	Weight	Std. Mean Difference IV, Random, 95% CI
**Endotoxin**								
Bajaj J S, 2014	−0.30	0.36	14	−0.40	0.54	16	12.4%	0.21 [−0.51, 0.93]
Liu Q, 2004	−26.70	19.60	20	−43.60	16.41	20	12.9%	0.92 [0.26, 1.57]
Shi J, 2023	−17.68	11.89	44	−10.89	11.37	44	14.6%	−0.58 [−1.01, −0.15]
**Total (95% CI)**			**78**			**80**	**39.9%**	**0.15 [−0.72, 1.03]**
Heterogeneity: $Tau^2 = 0.500$; $Chi^2 = 14.75$, df = 2 (P < .001); $I^2 = 86\%$								
Test for overall effect: Z = 0.34 (P = .731)								
**IL6**								
Bajaj J S, 2008	1.00	7.81	14	−1.00	5.00	6	10.5%	0.27 [−0.69, 1.23]
Bajaj J S, 2014	0.20	0.78	14	−0.40	3.58	16	12.4%	0.22 [−0.50, 0.94]
Shi J, 2023	−32.99	12.71	44	−14.78	12.83	44	14.3%	−1.41 [−1.88, −0.94]
**Total (95% CI)**			**72**			**66**	**37.2%**	**−0.36 [−1.49, 0.77]**
Heterogeneity: $Tau^2 = 0.854$; $Chi^2 = 18.95$, df = 2 (P < .001); $I^2 = 89\%$								
Test for overall effect: Z = −0.62 (P = .532)								
**TNF a**								
Bajaj J S, 2008	0.00	2.83	14	1.00	3.61	6	10.5%	−0.31 [−1.27, 0.65]
Bajaj J S, 2014	−1.80	6.88	14	−1.00	11.24	16	12.4%	−0.08 [−0.80, 0.64]
**Total (95% CI)**			**28**			**22**	**22.9%**	**−0.16 [−0.74, 0.41]**
Heterogeneity: $Tau^2 = 0$; $Chi^2 = 0.14$, df = 1 (P = .707); $I^2 = 0\%$								
Test for overall effect: Z = −0.56 (P = .575)								
**Total (95% CI)**			**178**			**168**	**100.0%**	**−0.13 [−0.65, 0.39]**
Heterogeneity: $Tau^2 = 0.439$; $Chi^2 = 42.04$, df = 7 (P < .001); $I^2 = 83\%$								
Test for overall effect: Z = −0.49 (P = .625)								
Test for subgroup differences: $Chi^2 = 0.57$, df = 2 (P = .752)								

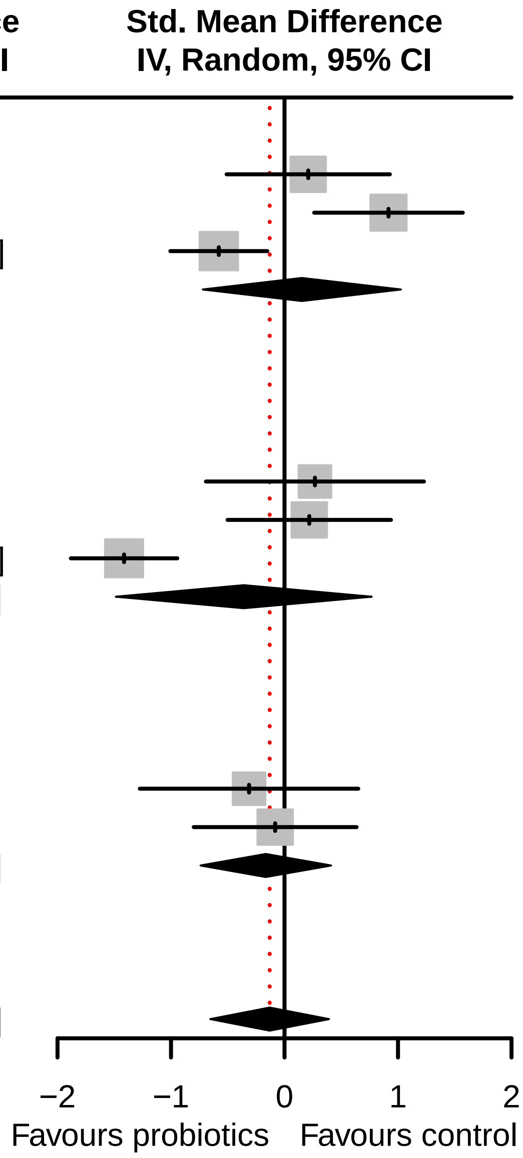

Figure S16. The forest plot of inflammatory cytokine expression

Study or Subgroup	Probiotics Events	Probiotics Total	Control Events	Control Total	Weight	Risk Ratio MH, Fixed, 95% CI
**2 months**						
Pratap Mouli V, 2015	2	60	0	60	0.8%	5.00 [0.25, 102.00]
Sharma K, 2014	1	32	2	30	3.4%	0.47 [0.04, 4.91]
**Total (95% CI)**		**92**		**90**	**4.3%**	**1.35 [0.28, 6.61]**
Heterogeneity: $Tau^2 = 0.959$; $Chi^2 = 1.5$, df = 1 (P = .220); $I^2 = 34\%$						
Test for overall effect: Z = 0.37 (P = .709)						
**3 months**						
Lunia M K, 2014	6	80	7	69	12.5%	0.74 [0.26, 2.10]
Roman E, 2019	1	18	1	18	1.7%	1.00 [0.07, 14.79]
**Total (95% CI)**		**98**		**87**	**14.1%**	**0.77 [0.29, 2.03]**
Heterogeneity: $Tau^2 = 0$; $Chi^2 = 0.04$, df = 1 (P = .838); $I^2 = 0\%$						
Test for overall effect: Z = −0.53 (P = .597)						
**6−12 months**						
Agrawal A, 2012	11	64	13	68	20.9%	0.90 [0.43, 1.86]
Dhiman R K, 2014	14	66	16	64	26.9%	0.85 [0.45, 1.59]
Pande C, 2012	13	45	14	43	23.7%	0.89 [0.47, 1.66]
**Total (95% CI)**		**175**		**175**	**71.6%**	**0.88 [0.60, 1.28]**
Heterogeneity: $Tau^2 = 0$; $Chi^2 = 0.02$, df = 2 (P = .992); $I^2 = 0\%$						
Test for overall effect: Z = −0.68 (P = .495)						
**Over 12 months**						
Efremova I, 2024	4	20	5	13	10.0%	0.52 [0.17, 1.58]
Test for overall effect: Z = −1.15 (P = .250)						
**Total (95% CI)**		**385**		**365**	**100.0%**	**0.85 [0.61, 1.18]**
Heterogeneity: $Tau^2 = 0$; $Chi^2 = 2.44$, df = 7 (P = .932); $I^2 = 0\%$						
Test for overall effect: Z = −1.00 (P = .319)						
Test for subgroup differences: $Chi^2 = 1.14$, df = 3 (P = .768)						

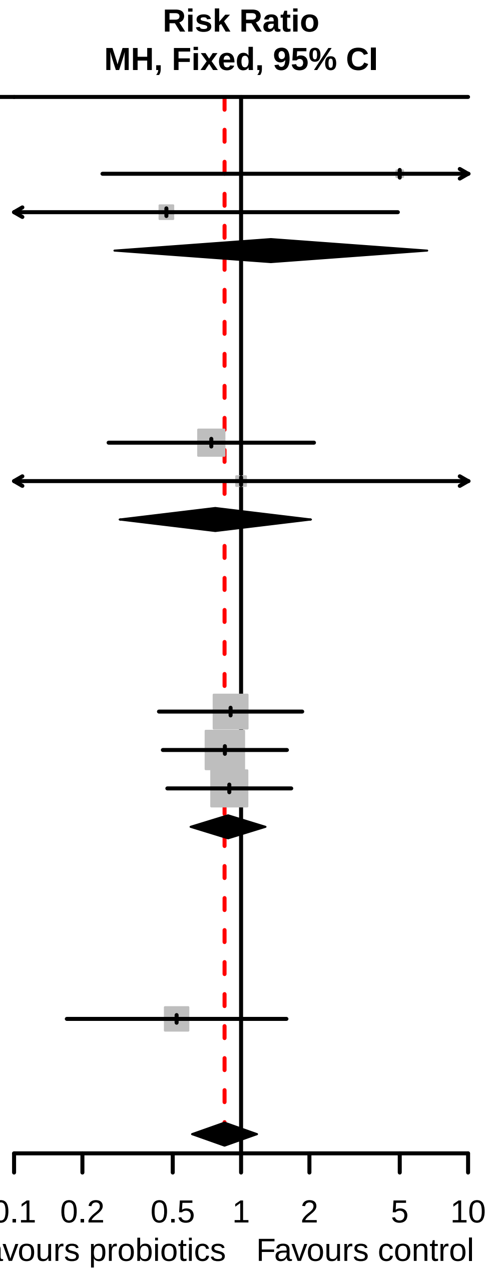

Figure S17. The forest plot of the effect of probiotics on mortality

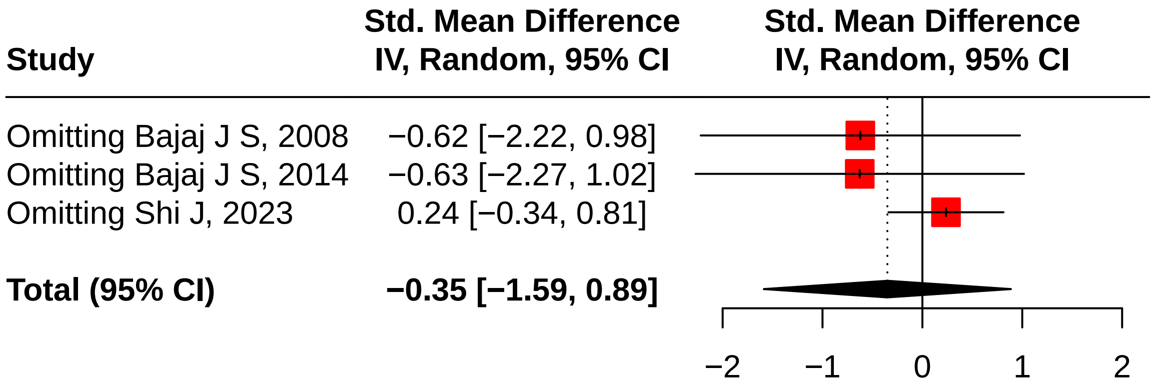

Figure S18. Sensitivity analysis of IL-6

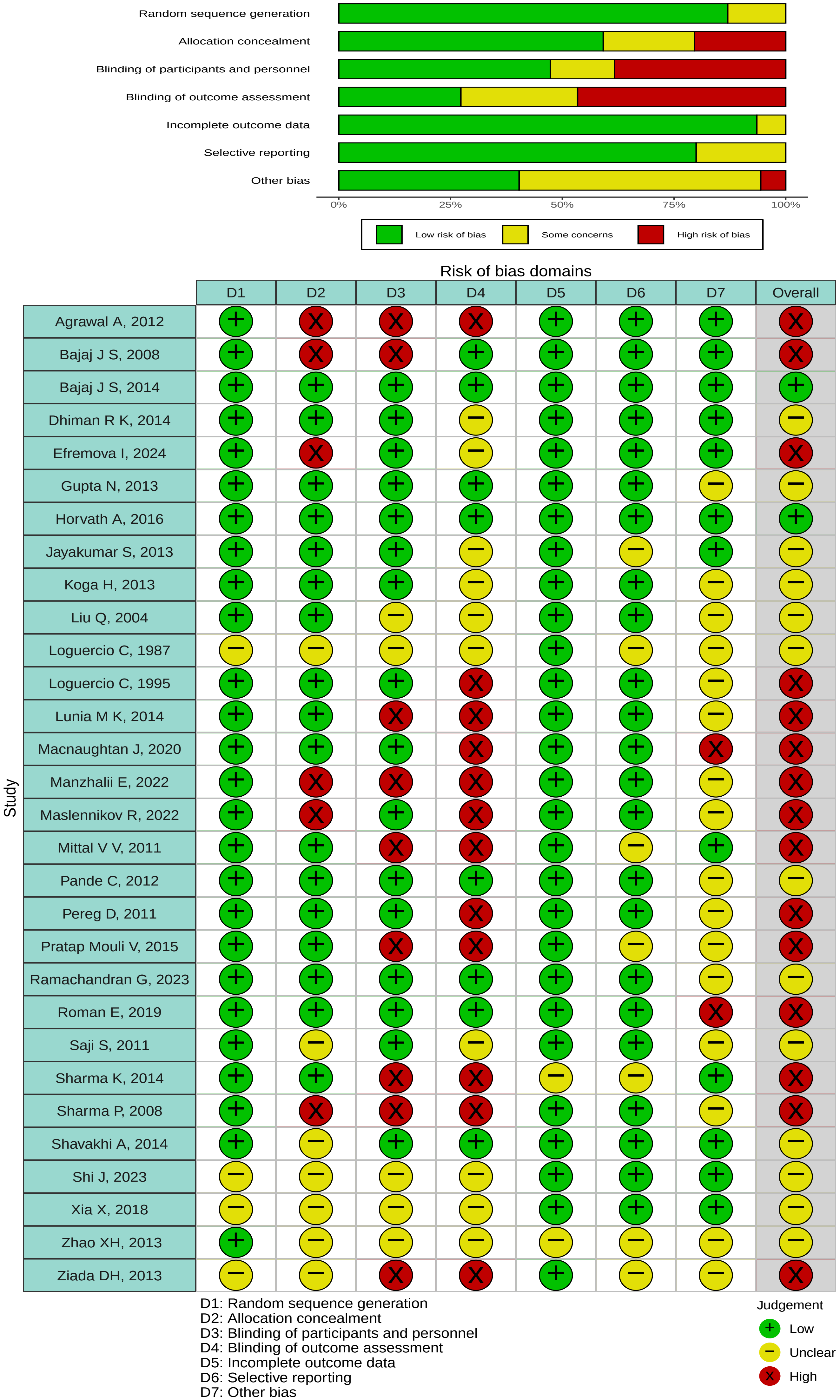

Figure S19. Quality assessment of risk of bias for the included studies

Figure S20a. Funnel plot of HE reversal incidence (*P* >0.05)

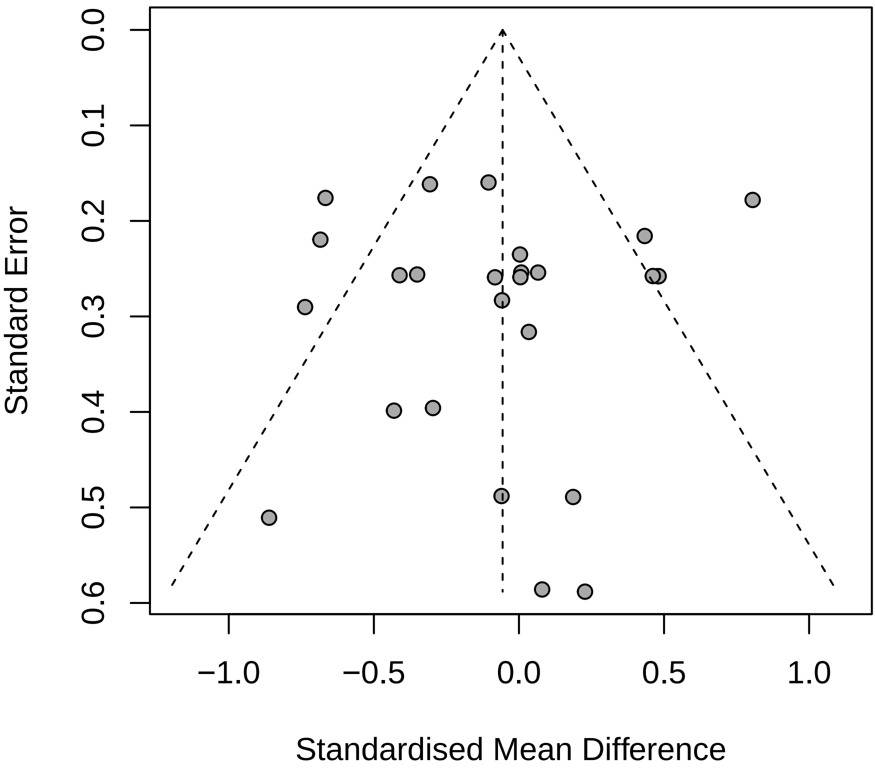

Figure S20b. Funnel plot of safety and tolerability of probitics (*P* >0.05)

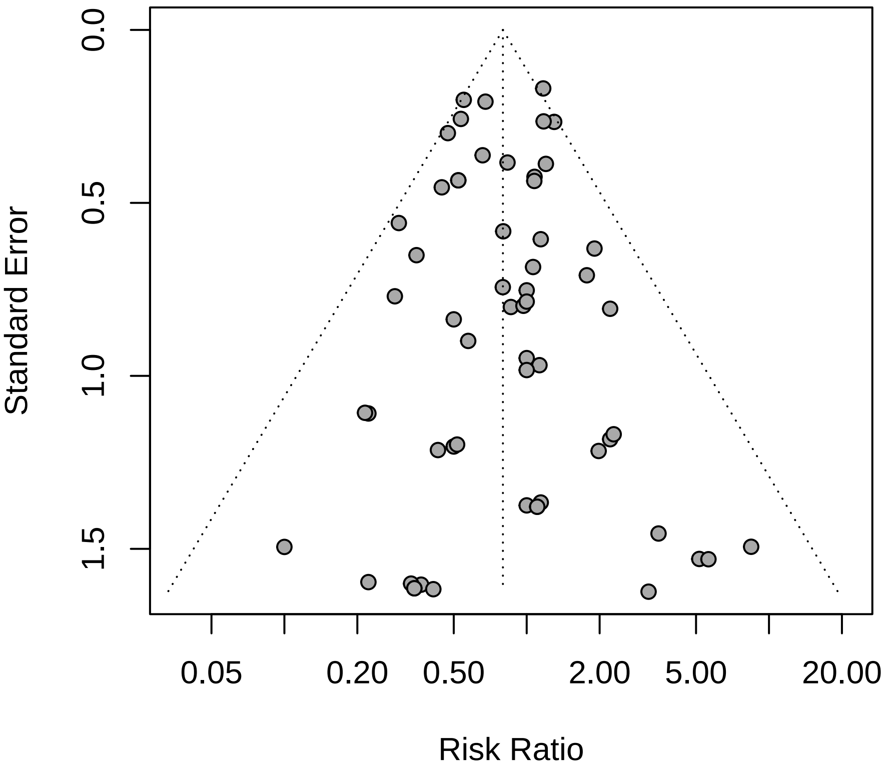

Figure S20c. Funnel plot of liver function (*P* >0.05)

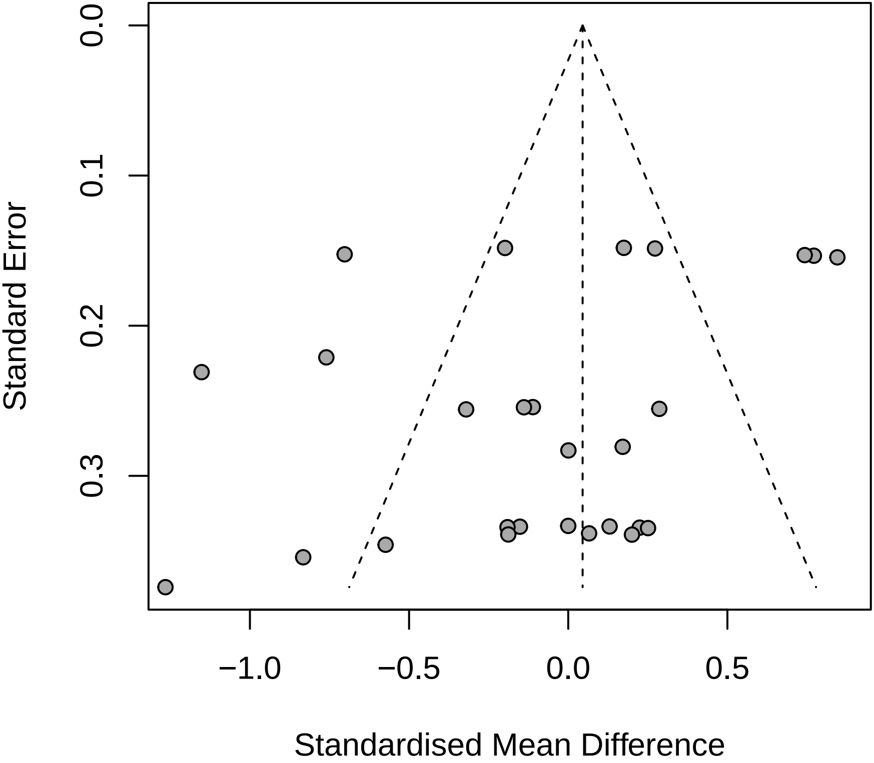

Figure S20d. Funnel plot of gut flora (*P* >0.05)

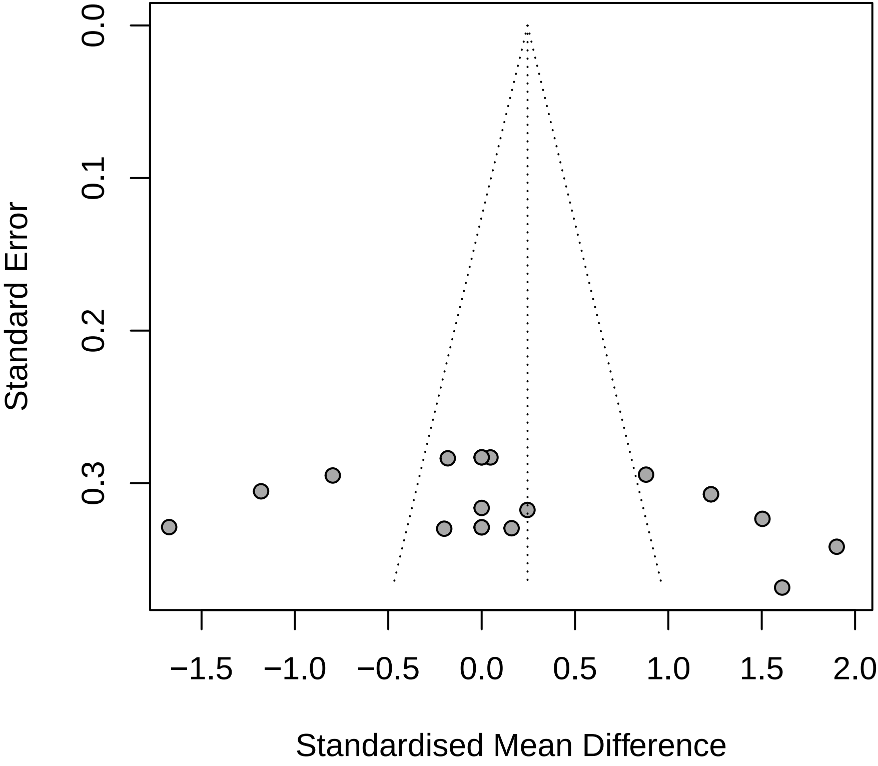

Figure S20. Publication bias of the main outcomes

Table S1. Search Strategy

PubMed		
**No.**	**Terms**	**Search strategy**
1	"cirrhosis"[MeSH] OR "liver cirrhosis"[tw] OR "cirrhosis of liver"[tw] OR "hepatic cirrhosis" [tw] OR "cirrhotic" [tw]	((#1 AND #2 AND #3) NOT #4)
2	"probiotic"[MeSH] OR "synbiotic"[MeSH] OR "probiotics"[tw] OR "probiotic bacteria"[tw] OR "probiotic supplement" [tw] OR "probiotic yogurt" [tw] OR "probiotic therapy" [tw] OR "synbiotics"[tw] OR "symbiotic"[tw] OR "symbiosis"[tw] OR "symbiotic supplement"[tw]	
3	("randomized controlled trial"[Mesh] OR "controlled clinical trial"[Mesh] OR "randomized"[tiab] OR "control"[tiab] OR "randomly"[tiab] OR "trial"[tiab]) NOT ("animals"[Mesh] OR "in vitro"[Mesh]) NOT ("nonrandom"[tiab] OR "non random"[tiab] OR "non-random"[tiab] OR "quasi-random"[tiab] OR "quasirandom"[tiab] OR "cohort study"[tiab] OR "cohort analysis"[tiab] OR "longitudinal studies"[tiab] OR "longitudinal study"[tiab] OR "prospective studies"[tiab] OR "prospective study"[tiab] OR "retrospective studies"[tiab] OR "retrospective study"[tiab] OR "observational studies"[tiab] OR "observational study"[tiab] OR "quasi experimental method"[tiab] OR "quasi experimental study"[tiab])	
4	"review"[pt] OR "letter"[pt] OR "meta-analysis"[pt] OR "case report"[pt] OR "editorial"[pt] OR "comment"[pt] OR "poster abstract"[pt] OR "correction"[pt]	
**Embase**		
**No.**	**Terms**	**Search strategy**
1	'cirrhosis'/exp OR 'liver cirrhosis' OR 'cirrhosis of liver' OR 'hepatic cirrhosis' OR 'cirrhotic'	
2	'probiotic'/exp OR 'synbiotic'/exp OR 'probiotics' OR 'probiotic bacteria' OR 'probiotic supplement' OR 'probiotic yogurt' OR 'probiotic therapy' OR 'synbiotics' OR 'symbiotic' OR 'symbiosis' OR 'symbiotic supplement'	

3	('randomized controlled trial'/exp OR 'controlled clinical trial'/exp OR 'random' OR 'control' OR 'clinical trials'/exp OR 'trial') NOT ('nonrandom' OR 'non random' OR 'non-random' OR 'quasi-random' OR 'quasirandom' OR 'cohort study' OR 'cohort analysis' OR 'longitudinal studies' OR 'longitudinal study' OR 'prospective studies' OR 'prospective study' OR 'retrospective studies' OR 'retrospective study' OR 'observational studies' OR 'observational study' OR 'quasi experimental methods' OR 'quasi experimental study')	((#1 AND #2 AND #3) NOT #4)
4	'review':it OR 'letter':it OR 'meta-analysis':it OR 'case report':it OR 'editoria':it OR 'comment':it OR 'poster abstract':it OR 'correction':it	
	**Web of Science**	
**No.**	**Terms**	**Search strategy**
1	cirrhosis (Topic) OR liver cirrhosis (Topic) OR cirrhosis of liver (Topic) OR hepatic cirrhosis (Topic) OR cirrhotic (Topic)	(#1 AND #2)
2	probiotic (Topic) OR synbiotic (Topic) OR probiotics (Topic) OR probiotic bacteria (Topic) OR probiotic supplement (Topic) OR probiotic yogurt (Topic) OR probiotic therapy (Topic) OR synbiotics (Topic) OR symbiotic (Topic) OR symbiosis (Topic) OR symbiotic supplement (Topic)	
	**Cochrane Library**	
**No.**	**Terms**	**Search strategy**
1	cirrhosis OR liver cirrhosis OR cirrhosis of liver OR hepatic cirrhosis OR cirrhotic	(#1 AND #2)
2	probiotic OR synbiotic OR probiotics OR probiotic bacteria OR probiotic supplement OR probiotic yogurt OR probiotic therapy OR synbiotics OR symbiotic OR symbiosis OR symbiotic supplement	

Table S2. Detailed characteristics of the included studies

Author, Year [Ref]	Database	Study setting	Study period	Target population	Inclusion criteria	Exclusion criteria	Interventions of treatment group (sample size)	Doses	Treatment Duration	Study Duration	Interventions of control group (sample size)
Agrawal A, 2012	Web of Science	India	2008. 10–2009. 12	Consecutive cirrhotic patients recovered from HE	(1)participants were 18–70 years of age; (2) had no overt HE but a previous history of HE.	(1) a history of taking lactulose in the past 6 weeks; (2) alcohol intake during the past 6 weeks; (3) receiving secondary prophylaxis for spontaneous bacterial peritonitis (SBP); (4) previous transjugular intrahepatic portosystemic shunt or shunt surgery; (5) significant comorbid illness, such as heart, respiratory, or renal failure, and any neurological disease, such as Alzheimer's disease, Parkinson's disease, and nonhepatic metabolic encephalopathies; (6) hepatocellular carcinoma; (7) receiving psychoactive drugs, such as antidepressants or sedatives; and (8) resumption of alcohol consumption during follow-up.	Probiotics: Lactobacillus (L. casei, L. plantarum, L. acidophilus, and L. delbrueckii subsp.bulgaricus), Bifidobacterium (B. longum, B. breve, and B. infantis), and Streptococcus salivarius subsp. thermophilus (n=77)	Three capsules of probiotics per day containing 1.125×10^{11} viable lyophilized bacteria per capsule.	3 months	12 months	(1) Lactulose (n=80); (2) No treatment (n=78)

(continued from previous page)

Author, Year [Ref]	Database	Study setting	Study period	Target population	Inclusion criteria	Exclusion criteria	Interventions of treatment group (sample size)	Doses	Treatment Duration	Study Duration	Interventions of control group (sample size)
Bajaj J S, 2008	PubMed	USA	2005. 10–2007. 1	Nonalcoholic MHE cirrhotics	(1) no alcohol intake within 3 months of the study; (2) a nonalcoholic etiology of cirrhosis.	(1) alcohol use within 3 months; (2) history of overt hepatic encephalopathy; (3) alcoholic etiology of cirrhosis; (4) antibiotic use within 6 week of the study; (5) current psychoactive medication use, diabetes mellitus, on current therapy for prevention or treatment of overt HE; (6) lack of English fluency.	Probiotic yogurt: S. thermophilus, L. bulgaricus, L. acidophilus, Bidobacteria, and L. casei (n=17)	Probiotic yogurt: Streptococcus thermophilus (log 9 CFU/g on Day 0) for 60 days, Lactobacillus bulgaricus (log 8.7 CFU/g on Day 0) for 60 days, Lactobacillus acidophilus and Lactobacillus casei (log 5.9 CFU/g on Day 0) for 60 days, Bifidobacteria (log 5.2 CFU/g on Day 0) for 60 days, 12 ounces, once daily.	2 months	2 months	No treatment (n=8)

(continued from previous page)

Author, Year [Ref]	Database	Study setting	Study period	Target population	Inclusion criteria	Exclusion criteria	Interventions of treatment group (sample size)	Doses	Treatment Duration	Study Duration	Interventions of control group (sample size)
Bajaj J S, 2014	Cochrane Library	USA	NA	Cirrhosis with MHE	(1) cirrhosis had been stable for 6 months without specific treatment changes; (2) age range 18–65 years.	(1) an unclear diagnosis of cirrhosis; (2) consumed alcohol within 6 months; (3) an upper gastrointestinal bleeding episode or need to be on systemic antibiotics within 6 weeks; (3) on current or past specific treatment for HE; (4) with hepatocellular cancer; (5) with yogurt/probiotic consumption within 2 weeks; (6) with inflammatory bowel disease, history of pancreatitis; (7) psychoactive medication use (apart from chronic anti-depressants); (8) with a recent absolute neutrophil count <500/mm; (9) liver transplant.	Probiotic: Lactobacillus GG (LGG) (n=14)	LGG batch had $>50\times10^9$ CFUs/g, 98 g/day or 87% of recommended.	2 months	2 months	Placebo (n=16)

(continued from previous page)

Author, Year [Ref]	Database	Study setting	Study period	Target population	Inclusion criteria	Exclusion criteria	Interventions of treatment group (sample size)	Doses	Treatment Duration	Study Duration	Interventions of control group (sample size)
Dhiman R K, 2014	Cochrane Library	India	2010.1−2012. 9	Cirrhosis recovered from HE	inpatients or outpatients diagnosed as having cirrhosis who had completely recovered from an episode of overt HE during the previous month	(1) a history of alcohol intake during the past 6 weeks; (2) active infection at the time of enrollment in the study; (3) any electrolyte imbalance; (4) hepatocellular carcinoma; (5) significant comorbid illness, such as cardiac, respiratory, or renal failure; (6) previous transjugular intrahepatic portosystemic shunt or shunt surgery; (7) any neurologic diseases, such as Alzheimer's disease, Parkinson's disease, and nonhepatic metabolic encephalopathies; (8) use of psychoactive drugs, such as antidepressants or sedatives.	Probiotic: VSL#3, containing Streptococcus thermophilus, Bifidobacterium breve, Bifidobacterium longum, Bifidobacterium infantis, L. acidophilus, Lactobacillus plantarum, Lactobacillus paracasei, L. bulgaricus (n=66)	One sachet of VSL#3, at a dose of 3, 9 $\times 10^{11}$ CFUs per sachet daily.	1 month	6 months	Placebo (n=64)

(continued from previous page)

Author, Year [Ref]	Database	Study setting	Study period	Target population	Inclusion criteria	Exclusion criteria	Interventions of treatment group (sample size)	Doses	Treatment Duration	Study Duration	Interventions of control group (sample size)
Efremova I, 2024	Web of Science	Russia	NA	Cirrhosis	(1) Cirrhosis diagnosed on the basis of histopathological or clinical, biochemical and ultrasonographic data; (2) Child–Pugh class B or C cirrhosis; (3) age from 18 to 70 years; (4) signed informed consent; and presence of small intestinal bacterial overgrowth (SIBO).	(1) intake of prebiotics, probiotics, antibiotics, or metformin within 6 weeks prior the inclusion; (2) alcohol consumption within 6 weeks prior to the inclusion; (3) inflammatory bowel disease, cancer, or any other serious illness; (4) patients who prematurely discontinued the consumption of tested probiotic/placebo, or started taking antibiotics, other probiotics, or prebiotics during the follow-up period, or refused to participate during the follow-up period.	Probiotic: S. boulardii CNCM I-745 (n=20)	A dose of 250 mg twice daily.	3 months	2 years	Placebo (n=13)

(continued from previous page)

Author, Year [Ref]	Database	Study setting	Study period	Target population	Inclusion criteria	Exclusion criteria	Interventions of treatment group (sample size)	Doses	Treatment Duration	Study Duration	Interventions of control group (sample size)
Gupta N, 2013	Cochrane Library	India	NA	Cirrhotic patients having large oesophageal varices	(1) diagnosis of cirrhosis; (2) no past history of upper GI bleeding; (3) endoscopically documented large oesophageal varices.	(1) history of gastrointestinal bleeding; (2) treatment with propranolol for portal hypertension in the past 6 weeks; (3) overt hepatic encephalopathy; (4) antibiotic treatment; (5) spontaneous bacterial peritonitis; (6) alcohol intake in the last 6 weeks or illicit drug abuse; (7) alcoholic hepatitis; (8) treatment with antibiotics or probiotics in the preceding 2 weeks; (9) hepatocellular carcinoma; (10) portal vein thrombosis; (11) serum creatinine >1.5 mg/dL; (12) treatment with vasoactive drugs in the past 6 weeks; (13) history of arterial hypertension, congestive heart failure, or arterial occlusive disease.	Probiotic: VSL#3 (n=31)	900 billion CFUs daily.	2 months	2 months	Placebo (n=32)

(continued from previous page)

Author, Year [Ref]	Database	Study setting	Study period	Target population	Inclusion criteria	Exclusion criteria	Interventions of treatment group (sample size)	Doses	Treatment Duration	Study Duration	Interventions of control group (sample size)
Horvath A, 2016	Cochrane Library	Austria	2012.7-2013.9	Cirrhosis	(1) age of 18 and 80 years; (2) willing to give informed consent.	(1) child–Pugh score 12 or higher; (2) alcohol abuse within 2 weeks prior to inclusion; (3) active infection at screening; (4) antibiotic therapy except for permanent prophylaxis; (5) simultaneous intake of pro-/pre-/symbiotic; (6) gastrointestinal haemorrhage within 2 weeks prior to inclusion; (7) immunomodulation drugs; (8) hepatic encephalopathy stage two or higher, renal failure (creatinine over 1.7 mg/dL), pancreatitis, other severe diseases unrelated to cirrhosis; (9) malignancy; (10) suspected noncompliance; (11) pregnancy.	Probiotic: Bifidobacterium bifidum W23, Bidobacterium lactis W52, Lactobacillus acidophilus W37, Lactobacillus brevis W63, Lactobacillus casei W56, Lactobacillus salivarius W24, Lactococcus lactis W19 and Lactococcus lactis W58 (n=44)	A daily dose of a multispecies probiotic (6 g, 2.5×10^9 CFUs/g).	6 months	12 months	Placebo (n=36)

(continued from previous page)

Author, Year [Ref]	Database	Study setting	Study period	Target population	Inclusion criteria	Exclusion criteria	Interventions of treatment group (sample size)	Doses	Treatment Duration	Study Duration	Interventions of control group (sample size)
Jayakumar S, 2013	PubMed (From reference lists of reviews)	Canada	2009. 10−2011. 5	Decompensated cirrhosis	(1) age ≥18 years; (2) cirrhosis diagnosed using either radiologica or histologica criteria; (3)Child–Pugh class B or C and clinically significant portal hypertension (HVPG ≥10 mm Hg).	(1) hepatocellular carcinoma beyond Milan criteria; (2) transjugular intrahepatic portosystemic shunt or surgical portosystemic shunt; (3) portal vein thrombosis, ongoing bacterial infection; (4) use of antibiotics within the 2 weeks prior to enrolment; (5) Grade III–IV hepatic encephalopathy; (6) gastrointestinal bleeding within the previous 2 weeks; (6) a history of myocardial infarction; (7) cerebrovascular accident; (8) arrhythmia, active drug or alcohol use; and (9) failure to consent.	Probiotic: VSL#3 (n=7)	Each sachet of VSL#3 contains 900 billion viable bacteria, totalling 3600 billion CFUs daily.	2 months	2 months	Placebo (n=8)

(continued from previous page)

Author, Year [Ref]	Database	Study setting	Study period	Target population	Inclusion criteria	Exclusion criteria	Interventions of treatment group (sample size)	Doses	Treatment Duration	Study Duration	Interventions of control group (sample size)
Koga H, 2013	PubMed	Japan	2005. 10–2006.10	Alcoholic cirrhosis	(1)age ≥ 20 years; (2)considered to have alcoholism, (3) with CTP grade A, but not grade B or C.	(1) Patients with elevated total bilirubin (C 3 mg/dL); (2) All patients who had taken probiotics, prebiotics, antibiotics, bile-acid drugs (i.e., ursodeoxycholic acid), and/or nutritional supplements within the month before enrolment.	Probiotic: beverage Yakult 400 (Y400) (n=18)	Y400, containing 40 billion CFUs of LcS (Lactobacillus casei Shirota YIT 9029) per serving, twice a day.	2 weeks	1 month	Placebo (n=19)

(continued from previous page)

Author, Year [Ref]	Database	Study setting	Study period	Target population	Inclusion criteria	Exclusion criteria	Interventions of treatment group (sample size)	Doses	Treatment Duration	Study Duration	Interventions of control group (sample size)
Liu Q, 2004	PubMed	China	NA	Cirrhosis with MHE	(1) Cirrhotic MHE patients without overt HE; (2) had been abstinent from alcohol for at least 2 months; (3) willing to give informed consent.	(1) histological features of alcoholic hepatitis; (2) the serum gamma-glutamyl transpeptidase level fell during a 2-month period of observation prior to study entry; (3) a history within the previous 6 weeks of factors that may have influenced gut flora and circulating endotoxin and ammonia levels, including infection, treatment with antibiotics, lactulose or immunomodulatory drugs, and gastrointestinal hemorrhage; (4) patients with other possible causes of reversible hepatic functional decompensation, such as drug-related hepatotoxicity and choledocholithiasis.	Synbiotic preparation (n=20)	Pediacoccus pentoseceus 5-33:3, Leuconostoc mesenteroides 32-77:1, Lactobacillus paracasei subspecies paracasei 19, and Lactobacillus plantarum 2592, each at a dose of 10^{10} CFUs per sachet, along with 10 g of bioactive, fermentable fiber (beta glucan, 2.5 g; inulin, 2.5 g; pectin, 2.5 g; resis-tant starch, 2.5 g).	1 month	1 month	Fermentable fiber (10 g of bioactive, fermentable fiber including beta glucan, 2.5 g; inulin, 2.5 g; pectin, 2.5 g; resis-tant starch, 2.5 g) (n=20)

(continued from previous page)

**Author, Year [Ref]**	**Database**	**Study setting**	**Study period**	**Target population**	**Inclusion criteria**	**Exclusion criteria**	**Interventions of treatment group (sample size)**	**Doses**	**Treatment Duration**	**Study Duration**	**Interventions of control group (sample size)**
Loguercio C, 1987	PubMed (From reference lists of reviews)	Italy	NA	Cirrhosis	NA	NA	Probiotic: Enterococcus SF68 (n=20)	Two capsules three times daily, one capsule containing at least 75×10^6 cells.	10 days	20 days	Lactulose (n=20)

(continued from previous page)

**Author, Year [Ref]**	**Database**	**Study setting**	**Study period**	**Target population**	**Inclusion criteria**	**Exclusion criteria**	**Interventions of treatment group (sample size)**	**Doses**	**Treatment Duration**	**Study Duration**	**Interventions of control group (sample size)**
Loguercio C, 1995	PubMed (From reference lists of reviews)	Italy	NA	Cirrhotic patients with HE	Patients with cirrhosis, with low grade l-2 hepatic encephalopathy of the chronic recurrent type, and ammonia plasma levels above 59 µM (normal values: 544 PM).	(1)grade 3-4 hepatic encephalopathy; (2)ascites that needed treatment with furosemide; (3)alcohol abuse or recent abstinence (<6 months); (4) liver tumour, and hepatorenal syndrome; (5) patients with severe sight disorders, colour blindness, alterations of the eye fundus and disorders of the anterior segment.	Probiotic: Enterococcus SF68 (n=21)	Two capsules containing a total amount of 150 million, three times daily for 4 weeks.	3 months	3 months	Lactulose (n=19)

(continued from previous page)

Author, Year [Ref]	Database	Study setting	Study period	Target population	Inclusion criteria	Exclusion criteria	Interventions of treatment group (sample size)	Doses	Treatment Duration	Study Duration	Interventions of control group (sample size)
Lunia M K, 2014	PubMed	India	2012.1−2013.3	Cirrhosis	Patients between ages 18 and 75 years with cirrhosis and no previous history of HE.	(1) patients on lactulose therapy; (2) patients with a history of recent alcohol intake (in the past 4 weeks); (3) recent infection or antibiotic use (in the past 6 weeks); (4) secondary prophylaxis for spontaneous bacterial peritonitis; (5) recent gastrointestinal bleeding, hepatocellular carcinoma, previous transjugular intrahepatic portosystemic shunting or shunt surgeries; (6) use of psychotropic drugs, neurologic diseases such as Alzheimer disease, Parkinson disease, and nonhepatic metabolic encephalopathies	Probiotic: VSL#3 (n=86)	1.1×10^{11} CFUs, one capsule, three times daily	3 months	3 months	Standard treatment (n=74)

(continued from previous page)

**Author, Year [Ref]**	**Database**	**Study setting**	**Study period**	**Target population**	**Inclusion criteria**	**Exclusion criteria**	**Interventions of treatment group (sample size)**	**Doses**	**Treatment Duration**	**Study Duration**	**Interventions of control group (sample size)**
Macnaughtan J, 2020	Web of Science	UK	NA	Cirrhosis	(1) Patients were aged between 18 and 78 years; (2) Patients were abstinent from alcohol for at least two weeks prior to the time of screening.	(1) Child–Pugh score greater than 10; (2) active infection; (3) any antibiotic treatment within 7 days prior to enrollment, gastrointestinal haemorrhage within 2 weeks, use of immunomodulating agents within 1 month; (4) use of proton pump inhibitors for the preceding 2 weeks; (5) concomitant use of supplements (pre-, pro- or synbiotics); (6) creatinine >150 mmol/L; (7) hepatic encephalopathy II to IV; (8) pancreatitis; (9) other organ failure; (10) hepatic or extrahepatic malignancy; (11) pregnancy.	Probiotic: Lactobacillus casei Shirota (LcS) (n=44)	A 65 mL bottle of LcS (6.5×10^9 CFUs/bottle), 3 times daily.	6 months	6 months	Placebo (n=43)

(continued from previous page)

Author, Year [Ref]	Database	Study setting	Study period	Target population	Inclusion criteria	Exclusion criteria	Interventions of treatment group (sample size)	Doses	Treatment Duration	Study Duration	Interventions of control group (sample size)
Manzhalii E, 2022	PubMed	Ukraine	2017. 1−2020. 3	Cirrhosis with MHE	(1) adult patients (age: 18-65 years); (2) the presence of minimal (Grade 1-2) HE; (3) two or more documented episodes of HE in the last 6 month, in addition to at least one episode in the last 3 mo; (4) a signed informed consent form	received L-ornithine-L-aspartate, zinc, metronidazole, neomycin, antibiotics, probiotics and yogurt consumption in the previous six weeks or had a history of allergy or intolerance to lactulose and/or rifaximin.	Probiotic: Escherichia coli Nissle 1917 strain (n=15)	2.5-25×10^9 CFUs/g, one capsule, twice daily.	1 month	1 month	(1) Lactulose (n=15); (2) Rifaximin (n=15)

(continued from previous page)

Author, Year [Ref]	Database	Study setting	Study period	Target population	Inclusion criteria	Exclusion criteria	Interventions of treatment group (sample size)	Doses	Treatment Duration	Study Duration	Interventions of control group (sample size)
Maslennikov R, 2022	Cochrane Library	Russia	NA	Consecutive cirrhosis	(1) Diagnosis of cirrhosis based on histopathological, or clinical, biochemical, and ultrasonographic findings; (2) Child-Pugh class B or C cirrhosis; (3) Age between 18 years and 70 years; (4) Signed informed consent.	(1) Administration of lactulose, lactitol, or other prebiotics, probiotics, antibiotics, or metformin during the 6 weeks preceding study commencement; (2) Alcohol consumption 6 weeks preceding study commencement; (3) Diagnosis of inflammatory bowel disease, cancer, or any other serious disease; (4) Patients who prematurely discontinued ingestion of the experimental probiotic/placebo; (5)were administered antibacterial drugs, other probiotics, or prebiotics during the follow-up period.	Probiotics:Saccharomyces boulardii (n=24)	A dose of 250 mg, twice daily.	3 months	3 months	Placebo (n=16)

(continued from previous page)

Author, Year [Ref]	Database	Study setting	Study period	Target population	Inclusion criteria	Exclusion criteria	Interventions of treatment group (sample size)	Doses	Treatment Duration	Study Duration	Interventions of control group (sample size)
Mittal V V, 2011	Cochrane Library	India	2007. 10–2009. 10	Cirrhosis with MHE	(1) Cirrhotic patients with MHE; (2) Cirrhosis was diagnosed on clinical basis involving laboratory tests, endoscopic evidence, sonographic findings, and liver histology.	(1) history of overt HE in the past 6 weeks; (2) recent history (< 6 weeks) of gastrointestinal bleeding; (3) active ongoing infection; (4) renal impairment with serum creatinine more than 1.5 mg%; (5) electrolyte impairment (serum sodium < 130 or > 150 meq/dl, serum potassium < 3.0 or > 5.5 meq/dl); (6) recent alcohol use (< 6 weeks) as reported by the patient; (7) recent use of antibiotic, lactulose, or LOLA (< 6 weeks); (8) use of psychotropic drugs in last 6 weeks; (9) transjugular intrahepatic portosystemic shunt, shunt surgery; (10) hepatocellular carcinoma; (11) severe comorbidity such as congestive heart failure, pulmonary disease, or poor vision precluding neuropsychiatric test.	Probiotics (n=40)	2.2×10^{11} CFUs, twice daily.	3 months	3 months	Standard treatment (n=40)

(continued from previous page)

Author, Year [Ref]	Database	Study setting	Study period	Target population	Inclusion criteria	Exclusion criteria	Interventions of treatment group (sample size)	Doses	Treatment Duration	Study Duration	Interventions of control group (sample size)
Pande C, 2012	Cochrane Library	India	2005. 4−2007. 8	Cirrhotic patients with ascites	(1) ascites with ascitic fluid protein concentration r1 g/dl; (2) serum bilirubin Z2.5 mg/dl; or (3) any past documented or ongoing episode of SBP.	(1)hepatic encephalopathy of grade III or IV at the time ofenrollment; (2) serum creatinine >2.0 mg/dl; (3) sepsis; (4) hepatocellular carcinoma or any other malignancy; (5) refusal to provide consent to participate in the trial.	Probiotics: E. faecalis JPC, C. butyricum, B. mesentericus JPC, Bacillus coagulans (n=55)	Probiotics capsules (containing E. faecalis JPC 30 million, C. butyricum 2 million, B. mesentericus JPC 1 million, Bacillus coagulans 50 million spores) at a dose of 2 capsules, three times daily.	6 months	6 months	Placebo (n=55)

(continued from previous page)

**Author, Year [Ref]**	**Database**	**Study setting**	**Study period**	**Target population**	**Inclusion criteria**	**Exclusion criteria**	**Interventions of treatment group (sample size)**	**Doses**	**Treatment Duration**	**Study Duration**	**Interventions of control group (sample size)**
Pereg D, 2011	Cochrane Library	Israel	NA	Cirrhosis	(1) One or more major complication of cirrhosis in the past; (2) Clinical evidence of portal hypertension; (3) Decreased hepatic synthetic function.	(1) presented with any sign of decompensation from any precipitant including gastrointestinal bleeding, infections, acute renal failure, electrolyte impairment, or hepatocellular carcinoma; (2) chronically treated with antibiotics or lactulose; (3) patients with alcoholic cirrhosis, for whom alcohol abstinence for at least 2 months prior to enrollment could not be confirmed.	Probiotic: Lactobacillus acidophilus, Lactobacillus bulgaricus, Bifidobacterium lactis, and Streptococcus thermophiles (n=20)	Probiotic capsules containing four freeze-dried bacteria, each at a daily dose of 2×10^{10} CFUs.	6 months	6 months	Placebo (n=20)

(continued from previous page)

Author, Year [Ref]	Database	Study setting	Study period	Target population	Inclusion criteria	Exclusion criteria	Interventions of treatment group (sample size)	Doses	Treatment Duration	Study Duration	Interventions of control group (sample size)
Pratap Mouli V, 2015	PubMed	India	2009.10–2012. 6	Cirrhosis with MHE	MHE patients with cirrhosis aged between 15 and 80 years.	(1)history of overt HE in the past 6 weeks; (2)history of intake of lactulose or probiotics or antibiotics or alcohol within the past 6 weeks; (3)presence of any other neurological or psychiatric diseases; (4)history of undergoing shunt surgery or transjugular intrahepatic portosystemic shunt for portal hypertension; (5) currently on medications which were likely to interfere with psychometric performance; (6)history of gastrointestinal bleeding or spontaneous bacterial peritonitis in the past 6 weeks; (7)presence of hepatocellular carcinoma, renal failure or portal vein thrombosis; (8)presence of significant comorbidities such as diabetes, congestive heart failure, or malignancy; (9) visual impairment.	Probiotic: VSL#3 (n=33)	Two capsule, each capsule contained 112.5 billion viable lyophilized bacteria, two times daily, a total of 4.5×10^{11} CFU.	2 months	2 months	Lactulose (n=40)

(continued from previous page)

Author, Year [Ref]	Databas e	Study setting	Study period	Target population	Inclusion criteria	Exclusion criteria	Interventions of treatment group (sample size)	Doses	Treatment Duration	Study Duration	Interventions of control group (sample size)
Ramachandran G, 2023	Embase	India	2021. 7–2022. 10	Cirrhosis	Patients between 18 years to 80 years, with liver cirrhosis referred for liver transplantation	(1) malignancy except hepatocellular carcinoma; (2)human immunodeficiency virus infection; (3) patients with cholestatic liver diseases like primary sclerosing cholangitis and cholangiocarcinoma; (4) chronic gastrointestinal disorder including inflammatory bowel disease and celiac disease; (5) chronic immunosuppressive medication usage; (6) consumption of probiotics in any form within one month prior to participation; (7) patients on antibiotics due to acute infection; (8) liver transplantation scheduled within 6 weeks from the day of enrolment.	Probiotics: VSL#3 (n=108)	Each VSL#3 capsule was equivalent to more than 1.125×10^{11} CFUs, two capsules daily.	6 weeks	6 weeks	Placebo (n=107)

(continued from previous page)

Author, Year [Ref]	Database	Study setting	Study period	Target population	Inclusion criteria	Exclusion criteria	Interventions of treatment group (sample size)	Doses	Treatment Duration	Study Duration	Interventions of control group (sample size)
Roman E, 2019	Embase	Spain	2013. 2–2016. 3	Consecutive outpatients with cirrhosis	Had cognitive dysfunction (a Psychometric Hepatic Encephalopathy Score of less than −4) and/or falls during the previous year.	(1) hospitalization during the previous month; (2) hepatocellular carcinoma or any other neoplasia; (3) acute or chronic overt hepatic encephalopathy, neurologic disease; (4) active alcohol intake (in the previous 3 months); (5) clinically significant cognitive impairment, inability to perform psychometric tests; (6) severe comorbidities, life expectancy of less than 6 months; (7) any treatment with nonabsorbable disaccharides, laxatives, antibiotics and/or antivirals in the previous 3 months; (8) refusal to participate in the study.	Probiotic: Streptococcus thermophilus DSM 24731, Bifidobacterium breve DSM 24732, B. longum DSM 24736, B. infantis DSM 24737, Lactobacillus paracasei DSM 24733, L. acidophilus DSM 24735, L. delbrueckii subsp bulgaricus DSM 24734, and L. plantarum DSM 24730 (n=18)	A sachet containing 4.5×10^{11} bacteria, twice daily.	3 months	5 months	Placebo (n=18)

(continued from previous page)

Author, Year [Ref]	Database	Study setting	Study period	Target population	Inclusion criteria	Exclusion criteria	Interventions of treatment group (sample size)	Doses	Treatment Duration	Study Duration	Interventions of control group (sample size)
Saji S, 2011	PubMed (From reference lists of reviews)	India	NA	Cirrhosis with MHE	(1) Stable cirrhotics in Child's grade A and B (diagnosed clinically, by ultrasonography or biopsy); (2) having minimal hepatic encephalopathy as per the NCT-A and evoked responses (auditory and visual).	(1) clinically evident hepatic encephalopathy, neurological disease; (2) alcohol free period of less than 2 months; (3) coexistent gastrointestinal hemorrhage, renal impairment, electrolyte disturbances; (4) those with severe visual or auditory abnormalities.	Probiotic: Lactobacillus acidophilus, Lactobacillus rhamnosus, Bifidobacterium longum, and Sacharomyces boulardi (n=21)	A dose of 1-gram sachet containing not less than 1.25 billion spores of Lactobacillus acidophilus, Lactobacillus rhamnosus, Bidobacterium longum, and Saccharomyces boulardii, 3 times daily after meals	1 month	1 month	Placebo (n=22)

(continued from previous page)

**Author, Year [Ref]**	**Database**	**Study setting**	**Study period**	**Target population**	**Inclusion criteria**	**Exclusion criteria**	**Interventions of treatment group (sample size)**	**Doses**	**Treatment Duration**	**Study Duration**	**Interventions of control group (sample size)**
Sharma K, 2014	Cochrane Library	India	2009. 8-2010.8	Cirrhosis with MHE	(1) a minimum of 2 years of education in school, (2) age over 18 years, (3) normal vision or corrected vision with the aid of specs/lens, (4) no alcohol abuse,(5) were not using any psychotrop-ic drug past 6 months.	(1) overt HE in the past 6 weeks; (2) alcohol intake past 6 weeks; (3) history of antibiotic or lactulose or probiotics use past 3 weeks; (4) gastrointestinal bleed past 6 weeks; (5) history of recent use of drugs (<6 weeks) effecting psychometric performance; (6) spontaneous bacterial peritonitis, or infection past 7 days; (7) renal insufficiency with creatinine >1.5 mg/Ll; (8) electrolyte imbalance; (9) comorbid illness; (10) previous transjuglar intrahepatic portosystemic shun; (12) restarted alcohol consumption during follow up; (13) inability to do psychometric tests; (14) not having knowledge of numbers and been to school for at least 2 years; (15) pregnant.	Probiotics (n = 32)	Two capsules daily, 5×10^9 CFUs: Lactobacillus acidophilus 0.7 billion; Lactobacillusm rhamnosus,Lactobacillus plantarum, Lactobacillus casei, Bifidobacterium longum, Bifidobacterium infantis, Bifidobacterium breve, and Streptococcus thermophilus 0.6 billion; Sacchromyces boulardi 0.1 billion.	2 months	2 months	Placebo (n =30)

(continued from previous page)

Author, Year [Ref]	Database	Study setting	Study period	Target population	Inclusion criteria	Exclusion criteria	Interventions of treatment group (sample size)	Doses	Treatment Duration	Study Duration	Interventions of control group (sample size)
Sharma P, 2008	PubMed	India	2005. 2 −2006.8	Cirrhosis with MHE	Consecutive cirrhotic patients without overt encephalopathy	(1) the presence of overt HE or history of hepatic encephalopathy (HE), history of taking lactulose; (2) any antibiotics, alcohol intake, gastrointestinal hemorrhage or spontaneous bacterial peritonitis during the past 6 weeks; (3) earlier transjugular intrahepatic portosystemic shunt or shunt surgery; (4) significant comorbid illness such as heart failure, respiratory failure, or renal failure; (5) any neurologic diseases such as Alzheimer's disease, Parkinson's disease and nonhepatic metabolic encephalo-pathies; (6) Patients with color blindness and mature cataract, diabetic retinopathy; (7) patients on psychoactive drugs, such as antidepressants or sedatives.	Probiotics: Streptococcus faecalis, Clostridium butyricum, Bacillus mesentricus, lactic acid bacillus (n=35)	One capsule three times per day, each capsule contained Streptococcus faecalis 60 million, Clostridium butyricum 4 million, Bacillus mesentricus 2 million, lactic acid bacillus 100 million.	1 month	1 month	Lactulose (n=35)

(continued from previous page)

Author, Year [Ref]	Database	Study setting	Study period	Target population	Inclusion criteria	Exclusion criteria	Interventions of treatment group (sample size)	Doses	Treatment Duration	Study Duration	Interventions of control group (sample size)
Shavakhi A, 2014	PubMed	Iran	2012.6 -2012. 10	Cirrhosis with MHE	Adult patients with MHE	Patients with overt HE, known brain lesions, active gastrointestinal bleeding, active ongoing infection, renal impairment (serum creatinine >2 mg/dL), electrolyte abnormalities (serum sodium <130 or >150 meq/dL, serum potassium <3.0 or >5.5 meq/dL), and those who received HE treatments such as lactulose and antibiotics or consumed benzodiazepines, narcotics, opioids, or alcohol in the preceding 8 weeks were not included into the trial.	Synbiotics: Probiotic (Lactobacillus, Bifidobacterium, and Streptococcus thermophiles) and Lactulose (n=19)	Probiotic: One capsule containing 1×10^8 CFU of seven bacteria species, twice daily. Lactulose: 30−60 mL/day in divided doses.	2 weeks	10 weeks	Lactulose (30–60 mL/day in divided doses) + Placebo (n=21)

(continued from previous page)

Author, Year [Ref]	Database	Study setting	Study period	Target population	Inclusion criteria	Exclusion criteria	Interventions of treatment group (sample size)	Doses	Treatment Duration	Study Duration	Interventions of control group (sample size)
Shi J, 2023	Cochrane Library	China	2020. 8 −2021. 8	Cirrhosis with MHE	(1) patients with cirrhotic MHE; (2) willing to give informed consent.	(1) patients with a history of hepatic encephalopathy; (2) incomplete NCT-A and DST because of culture degree; (3) mental illness and other reasons; (4) alcoholic cirrhosis and do not give up drinking; (5) pregnancy and lactation; (6) merge with other drop ammonia drugs; (7) serious heart, lung, kidney, brain diseases, serious diabetes complications; (8) gastrointestinal bleeding 2 weeks before the start of the study and electrolyte disturbances.	Synbiotics: Probiotic (Bacillus subtilis and Enterococcus faecium) and Lactulose (n=44)	Probiotic: One capsule contains 500 million live probiotics, including 5.0×10^{7} Bacillus subtilis and 4.5×10^{8} Enterococcus faecium, two capsules a time, three times daily. Lactulose: initial dose was 30 ml daily, the maintenance dose was 15 ml, three times daily.	2 weeks	2 weeks	Lactulose (initial dose was 30 ml daily, the maintenance dose was 15 ml, three times daily) (n=44)

(continued from previous page)

**Author, Year [Ref]**	**Database**	**Study setting**	**Study period**	**Target population**	**Inclusion criteria**	**Exclusion criteria**	**Interventions of treatment group (sample size)**	**Doses**	**Treatment Duration**	**Study Duration**	**Interventions of control group (sample size)**
Xia X, 2018	Cochrane Library	China	NA	Cirrhosis with MHE	Consecutive patients with HBV-induced cirrhosis without overt hepatic encephalopathy.	(1) a history of overt HE in the past 2 months; (2) the use of probiotics, prebiotics, synbiotics, or antibiotics during the previous 4 weeks; (3) the presence of any other neurological or psychiatric diseases; (4) a history of spontaneous bacterial peritonitis or gastrointestinal bleeding in the past 2 months; (5) known active microbial infections; (6) a high-protein diet.	Probiotics: Clostridium butyricum and Bifidobacterium infantis (n=30)	One capsule containing more than 1×10^7 CFU/g viable C.butyricum, and more than 1×10^6 CFU/g viable B.infantis, 1500mg, three times daily.	3 months	3 months	Standard treatment (n=37)

(continued from previous page)

Author, Year [Ref]	Database	Study setting	Study period	Target population	Inclusion criteria	Exclusion criteria	Interventions of treatment group (sample size)	Doses	Treatment Duration	Study Duration	Interventions of control group (sample size)
Zhao XH, 2013	Cochrane Library (From reference lists of reviews)	China	NA	Cirrhosis with MHE	(1)Compliance with chronic hepatitis B prevention and treatment guidelines; (2) Diagnosed with MHE based on psychometric testing.	(1) Clinical symptoms of HE; (2) Clinical symptoms of HE in last 6 weeks; (3) History of upper GI bleeding in the last 6 weeks; (4) active infection; (5) Renal impairment and creatinine greater than 133 μM/L; (6) Electrolyte abnormality (Na+ < 130 mM/L or > 150 mM/L; K+ < 3 mM/L or > 5.5mM/L); (7) People with alcoholic cirrhosis; (8) Recently taking antibiotics, probiotics, or aspartate/ornithine; (9) Took psychotropic drugs in the last 6 weeks; (10) TIPS shunt; (11) Surgery; (12) Liver tumours; (13) Serious systemic disease such as heart failure, pulmonary disease, neurological and psychiatric illness; (14) Visual impairment; (15) Impairment on intelligence tests.	Probiotic: subtype not available (n=40)	110 million CFU, twice daily.	1 month	1 month	Placebo (n=40)

(continued from previous page)

Author, Year [Ref]	Database	Study setting	Study period	Target population	Inclusion criteria	Exclusion criteria	Interventions of treatment group (sample size)	Doses	Treatment Duration	Study Duration	Interventions of control group (sample size)
Ziada DH, 2013	PubMed	Egypt	2010. 3–2012. 1	Cirrhosis with MHE	(1) Patients with cirrhosis had MHE; (2) willing to give informed consent.	(1) the presence of overt HE, alcohol intake, gastrointestinal haemorrhage or spontaneous bacterial peritonitis during the past 6 weeks; (2) previous shunt surgery and associated heart, respiratory or renal failure as well as history of any neurologic or metabolic encephalopathies; (3) Patients on psychoactive drugs, such as antidepressants or sedatives.	Probiotic: L. acidobacillus acidophilus (n=30)	One capsule containing 1×10^7 Lactobacillus acidophilus, three times daily.	1 month	1 month	Lactulose (n=30)

Table S3. Data and outcome measurements of the included studies

Author, Year [Ref]	HE reversal	Safety and tolerability	Liver function measurements	Quality of life	Effect on gut flora	Serum inflammatory cytokines change	Mortality
Agrawal A, 2012	**Baseline:**<br>**Arterial ammonia:** Probiotics: 88.2±20.6 (n=77), Lactulose: 93.2±19.0 (n=80), No treatment: 89.8±18.6 (n=78)<br><br>**3 months:**<br>**Arterial ammonia:** Probiotics: 75.2±20.9 (n=77), Lactulose: 82.97±12.9 (n=80), No treatment: 85.2±16.7 (n=78)	**12 months:**<br>**Adverse events:**<br>**UTI:** Probiotics: 1/64, Lactulose: 0/68, No treatment: 2/65<br>**Constipation:** Probiotics: 5/64, Lactulose: 3/68, No treatment: 8/65<br>**Variceal bleed:** Probiotics: 4/64, Lactulose: 4/68, No treatment: 5/65<br>**SBP:** Probiotics: 3/64, Lactulose: 4/68, No treatment: 7/65<br><br>**Serious adverse events:**<br>**Hospitalization:** Probiotics: 21/64, Lactulose: 19/68, No treatment: 28/65<br>**Overt HE:** Probiotics: 22/64, Lactulose: 18/68, No treatment: 37/65					**12 months:**<br>Probiotics: 11/64, Lactulose: 13/68, No treatment: 16/65

(continued from previous page)

Author, Year [Ref]	HE reversal	Safety and tolerability	Liver function measurements	Quality of life	Effect on gut flora	Serum inflammatory cytokines change	Mortality
Bajaj J S, 2008	**Baseline:**<br>**Venous ammonia:** Probiotics: 46±24 (n=17), No treatment: 34±20 (n=8)<br>**NCT-A:** Probiotics: 43±9 (n=14), No treatment: 48±17 (n=6)<br>**DST:** Probiotics: 60±8 (n=14), No treatment: 48±14 (n=6)<br>**BDT:** Probiotics: 36±3 (n=14), No treatment: 25±8 (n=6)<br><br>**2 months:**<br>**MHE reversal:** Probiotics: 12/17, No treatment: 0/8<br>**Venous ammonia:** Probiotics: 50±26 (n=14), No treatment: 40±3 (n=6)<br><br>**Neuropsychometric test:**<br>**NCT-A:** Probiotics: 30±4 (n=14), No treatment: 47±10 (n=6)<br>**DST:** Probiotics: 69±8 (n=14), No treatment: 54±18 (n=6)<br>**BDT:** Probiotics: 41±5 (n=14), No treatment: 28±10 (n=6)	**Serious adverse events:**<br>**1 month:**<br>**Overt HE:** Probiotics: 0/17, No treatment: 1/8<br>**2 months:**<br>**Overt HE:** Probiotics: 0/17, No treatment: 2/8<br><br>**Nonadherence:**<br>**1 month:** Probiotics: 2/17, No treatment: 0/8<br>**2 months:** Probiotics: 3/17, Control: 0/8	**2 months:**<br>**MELD scores:** Probiotics: 9±3 (n=14), No treatment: 10±4 (n=6)<br>**Child–Pugh class A:** Probiotics: 14/14, No treatment: 5/6<br>**Child–Pugh class B:** Probiotics: 0/14, No treatment:1/6	**2 months:**<br>**SF-36 (short-form 36 questionnaire) physical:** Probiotics: 39±5 (n=14), No treatment: 39±6 (n=6)<br>**SF-36 mental:** Probiotics: 46±3 (n=14), No treatment: 42±7 (n=6)		**Baseline:**<br>**IL-6:** Probiotics: 6±6 (n=17), No treatment: 4±4 (n=8)<br>**TNF-α:** Probiotics: 3±2 (n=17), No treatment: 3±2 (n=8)<br><br>**2 months:**<br>**IL-6:** Probiotics: 7±5 (n=14), No treatment: 3±3 (n=6)<br>**TNF-α:** Probiotics: 3±2 (n=14), No treatment: 4±3 (n=6)	

(continued from previous page)

Author, Year [Ref]	HE reversal	Safety and tolerability	Liver function measurements	Quality of life	Effect on gut flora	Serum inflammatory cytokines change	Mortality
Bajaj J S, 2014		**2 months:**<br>**Adverse events:**<br>**Abdominal pain:** Probiotic: 4/14, Placebo: 4/16<br>**Bloating:** Probiotic: 5/14, Placebo: 3/16<br>**Skin rash:** Probiotic: 2/14, Placebo: 1/16<br><br>**Serious adverse events:**<br>**Infections:** Probiotic: 1/14, Placebo: 1/16				**Baseline:**<br>**IL-6:** Probiotics: 1.5±4.8 (n=14), Placebo: 1.1±2.9 (n=16)<br>**TNF-α:** Probiotics: 10.8±5.6 (n=14), Placebo: 12.2±8.1 (n=16)<br>**Endotoxin:** Probiotics: 0.4±0.5 (n=14), Placebo: 0.2±0.2 (n=16)<br><br>**2 months:**<br>**IL-6:** Probiotic: 1.7±5.2 (n=14), Placebo: 0.7±2.1 (n=16)<br>**TNF-α:** Probiotic: 9.0±4.0 (n=14), Placebo: 11.2±7.8 (n=16)<br>**Endotoxin change:** Probiotic: 0.1±0.1 (n=14), Placebo: 0.3±0.5 (n=16)	

(continued from previous page)

Author, Year [Ref]	HE reversal	Safety and tolerability	Liver function measurements	Quality of life	Effect on gut flora	Serum inflammatory cytokines change	Mortality
Dhiman R K, 2014		**6 months:**<br>**Adverse events:**<br>**Constipation:** Probiotic: 10/66, Placebo: 9/64<br>**SBP:** Probiotic: 7/66, Placebo: 13/64<br>**Upper gastrointestinal (GI) bleeding:** Probiotic: 4/66, Placebo: 7/64<br><br>**Serious adverse events:**<br>**Overt HE:** Probiotic: 23/66, Placebo: 33/64<br>**Hospitalization:** Probiotics: 16/66, Placebo: 29/64<br><br>**Nonadherence:**<br>Probiotics: 3/66, Placebo: 3/64					**6 months:**<br>Probiotic: 14/66, Placebo: 16/64

(continued from previous page)

Author, Year [Ref]	HE reversal	Safety and tolerability	Liver function measurements	Quality of life	Effect on gut flora	Serum inflammatory cytokines change	Mortality
Efremova I, 2024		**3 months:**<br>**Adverse events:**<br>**Variceal bleed:** Probiotics: 18/20, Placebo: 10/13<br>**Ascites:** Probiotics: 8/20, Placebo: 11/13<br><br>**Serious adverse events:**<br>**Overt HE:** Probiotics: 0/20, Placebo: 1/13					**1 year:**<br>Probiotic: 2/20, Placebo: 5/13<br><br>**2 year:**<br>Probiotic: 4/20, Placebo: 5/13
Gupta N, 2013		**2 months:**<br>**Adverse events:**<br>**Adverse events:** Probiotics: 5/31, Placebo: 5/32<br>**UTI:** Probiotics: 0/31, Placebo: 1/32<br>**SBP:** Probiotics: 2/31, Placebo: 0/32<br>**Upper GI bleed:** Probiotics: 1/31, Placebo: 2/32<br>**Skin rash:** Probiotics: 0/31, Placebo: 1/32					

(continued from previous page)

Author, Year [Ref]	HE reversal	Safety and tolerability	Liver function measurements	Quality of life	Effect on gut flora	Serum inflammatory cytokines change	Mortality
Horvath A, 2016		**Serious adverse events:**<br>**6 months:**<br>**Infections:** Probiotic: 15/44, Placebo: 28/36<br>**12 months:**<br>**Infections:** Probiotic: 6/44, Placebo: 11/36	**3 months:**<br>**Child–Pugh class A:**<br>Probiotics: 31/44, Placebo: 33/36<br>**6 months:**<br>**Child–Pugh class A:**<br>Probiotics: 29/44, Placebo: 33/36<br>**12 months:**<br>**Child–Pugh class A:**<br>Probiotics: 24/44, Placebo: 33/36				
Jayakumar S, 2013			**2 months:**<br>**MELD scores:** Probiotics: 11±5.2 (n=7), Control: 13.5±3.6 (n=8)			**2 months:**<br>**IL-6:** Probiotic: -0.9±140 (n=5), Placebo:3.5±48 (n=8)	

(continued from previous page)

Author, Year [Ref]	HE reversal	Safety and tolerability	Liver function measurements	Quality of life	Effect on gut flora	Serum inflammatory cytokines change	Mortality
Koga H, 2013					**Baseline:**<br>**Bifidobacterium:** Probiotic: 8.3±1.0 (n=18), Placebo: 8.5±1.0 (n=19)**Enterobacteriaceae:** Probiotic: 8.4±1.2 (n=18), Placebo: 8.8±0.8 (n=19)<br>**Bacteroidaceae:** Probiotic: 7.8±1.2 (n=18), Placebo: 8.0±1.3 (n=19)<br>**Fusobacterium:** Probiotic: 7.3±1.1 (n=18), Placebo: 7.4±1.1 (n=19)<br><br>**2 weeks:**<br>**Bifidobacterium:** Probiotic: 8.6±0.6 (n=18), Placebo: 8.6±1.0 (n=19)<br>**Enterobacteriaceae:** Probiotic: 7.7±1.3 (n=18), Placebo: 8.3±1.0 (n=19)<br>**Bacteroidaceae:** Probiotic: 8.8±0.8 (n=18), Placebo: 8.9±0.6 (n=19)<br>**Fusobacterium:** Probiotic: 7.3±1.0 (n=18), Placebo: 7.0±0.9 (n=19)<br><br>**1 month:**<br>**Bifidobacterium:** Probiotic: 8.7±0.7 (n=18), Placebo: 8.7±0.7 (n=19)<br>**Enterobacteriaceae:** Probiotic: 7.7±1.2 (n=18), Placebo: 8.4±0.9 (n=19)<br>**Bacteroidaceae:** Probiotic: 9.0±0.6 (n=18), Placebo: 9.2±0.7 (n=19)<br>**Fusobacterium:** Probiotic: 7.6±0.9 (n=18), Placebo: 7.3±1.0 (n=19)<br>**(log10 CFU/g faecal sample)**		

(continued from previous page)

Author, Year [Ref]	HE reversal	Safety and tolerability	Liver function measurements	Quality of life	Effect on gut flora	Serum inflammatory cytokines change	Mortality
Liu Q, 2004	**Baseline:**<br>**Venous ammonia:**<br>Sybiotics: 60.5±2.9 (n=20), Fermentable fiber: 63.6±3.9 (n=20) (P=0.008)<br><br>**1 month:**<br>**MHE reversal:**<br>Synbiotics: 10/20, Fermentable fiber: 10/20<br>**Venous ammonia:**<br>Sybiotics: 38.6±3.9 (n=20), Fermentable fiber: 41.5±5.2 (n=20)		**1 month:**<br>**Child–Pugh class A:**<br>Synbiotics: 6/17, Fermentable fiber: 4/17<br>**Child–Pugh class B:**<br>Synbiotics: 2/17, Fermentable fiber: 1/13		**Baseline:**<br>**Bifidobacterium:** Synbiotics: 6.6±0.7 (n=20), Fermentable fiber: 6.9±0.7 (n=20)<br>**Lactobacillus:** Synbiotics: 7.4±0.5 (n=20), Fermentable fiber: 7.2±1.3 (n=20)<br>**Enterococcus:** Synbiotics: 5.9±1.3 (n=20), Fermentable fiber: 6.1±1.7 (n=20)<br>**Fusobacterium:** Synbiotics: 7.5±0.4 (n=20), Fermentable fiber: 7.2±0.6 (n=20)<br><br>**1 month:**<br>**Bifidobacterium:** Synbiotics: 7.1±0.7 (n=20), Fermentable fiber: 9.2±0.3 (n=20)<br>**Lactobacillus:** Synbiotics: 9.8±0.6 (n=20), Fermentable fiber: 7.4±0.9 (n=20)<br>**Enterococcus:** Synbiotics: 6.4±1.4 (n=20), Fermentable fiber: 6.2±1.0 (n=20)<br>**Fusobacterium:** Synbiotics: 6.2±0.4 (n=20), Fermentable fiber: 4.3±1.5 (n=20)<br><br>**1.5 months:**<br>**Bifidobacterium:** Synbiotics: 7.4±0.8 (n=20), Fermentable fiber: 7.7±0.6 (n=20)<br>**Lactobacillus:** Synbiotics: 9.6±0.3 (n=20), Fermentable fiber: 7.5±0.8 (n=20)<br>**Enterococcus:** Synbiotics: 6.5±1.4 (n=20), Fermentable fiber: 6.2±1.2 (n=20)<br>**Fusobacterium:** Synbiotics: 6.8±0.4 (n=20); Fermentable fiber: 8.0±1.0 (n=20)	**Baseline:**<br>**Endotoxin:**<br>Synbiotics: 110.0±14.3 (n=20), Fermentable fiber: 112.1±14.1 (n=20) (P=0.65)<br><br>**1 month:**<br>**Endotoxin:**<br>Synbiotics: 83.3±13.4 (n=20), Fermentable fiber: 68.5±8.4 (n=20)	

(continued from previous page)

Author, Year [Ref]	HE reversal	Safety and tolerability	Liver function measurements	Quality of life	Effect on gut flora	Serum inflammatory cytokines change	Mortality
Loguercio C, 1987	**20 days:**<br>**Improvement of HE:** Probiotics: 9/15, Lactulose: 6/14						
Loguercio C, 1995	**Baseline:**<br>**Arterial ammonia: Sybiotics:** 86.3±29.8 (n=21), Lactulose: 84.6±14.2 (n=19)<br>**NCT-A:** Sybiotics: 142.8±63.4 (n=21), Lactulose: 144.4±79.1 (n=19)<br><br>**1 month:**<br>**Improvement of HE:** Probiotics: 14/21, Lactulose: 7/19<br><br>**Arterial ammonia:**<br>**1 month:** Probiotics: 57.3±19.8 (n=18), Lactulose: 62.7±17.3 (n=19)<br>**2 months:**Probiotics: 47.4±12.3 (n=15), Lactulose: 58.5±25.1 (n=16)<br>**3 months:**Probiotics: 45.6±8.3 (n=14), Lactulose: 58.3±30.9 (n=12)<br><br>**NCT-A:**<br>**1 month:** Probiotics: 63.5±13.3 (n=18), Lactulose: 87.9±30.4 (n=19)<br>**2 months:** Probiotics: 62.7±17.6 (n=15), Lactulose: 74.4±15.9 (n=16)<br>**3 months:** Probiotics: 50.9±9 (n=14), Lactulose: 75±26.3 (n=12)						

(continued from previous page)

Author, Year [Ref]	HE reversal	Safety and tolerability	Liver function measurements	Quality of life	Effect on gut flora	Serum inflammatory cytokines change	Mortality
Lunia M K, 2014	**Baseline:**<br>**Arterial ammonia:** Probiotics: 74.3±18.6 (n=86), Control: 78.4±15.6 (n=74)<br>**CFF:** Probiotics: 40.4±8.8 (n=86), Control: 41.8±9.9 (n=74)<br><br>**3 months:**<br>**Venous ammonia:** Probiotics: 61.2±15.2 (n=76), Control: 81.3±17.8 (n=62)<br>**Neurophysiological test:**<br>**CFF:** Probiotics: 49.9±10.4 (n=76), Control: 39.2±13.1 (n=62)	**3 months:**<br>**Adverse events:**<br>**UTI:** Probiotics: 1/86, Control: 2/74<br>**Constipation:** Probiotics: 3/86, Control: 3/74<br>**Variceal bleed:** Probiotics: 2/86, Control: 3/74<br>**SBP:** Probiotic: 1/86, Control: 4/74<br><br>**Serious adverse events:**<br>**Overt HE:** Probiotic: 4/76, Placebo: 11/62					**3 months:**<br>Probiotics: 6/80, Control: 7/69

(continued from previous page)

Author, Year [Ref]	HE reversal	Safety and tolerability	Liver function measurements	Quality of life	Effect on gut flora	Serum inflammatory cytokines change	Mortality
Macnaughtan J, 2020		**1 month:**<br>**Adverse events:**<br>**Ascites:** Probiotic: 2/33, Placebo: 2/41<br>**6 months:**<br>**Ascites:** Probiotic: 2/31, Placebo: 2/35<br>**Variceal Hemorrhage:** Probiotic: 2/31, Placebo: 0/35<br><br>**Serious adverse events:**<br>**1 month:**<br>**Infections:** Probiotic: 0/33, Placebo: 1/41		**Baseline:**<br>**SF-36 physical:** Probiotics: 58.43±32.35 (n=33), Placebo: 67.66±32.95 (n=35)<br>**1 month:**<br>**SF-36 physical:** Probiotics: 63±33.57 (n=33), Placebo: 75.17±27.14 (n=35)<br>**6 months:**<br>**SF-36 physical:** Probiotics: 65.86±53.23 (n=33), Placebo: 68.83±30.19 (n=35)			

(continued from previous page)

Author, Year [Ref]	HE reversal	Safety and tolerability	Liver function measurements	Quality of life	Effect on gut flora	Serum inflammatory cytokines change	Mortality
Manzhalii E, 2022	**Neurophychometric test:** **Baseline:** **Stroop's test:** Probiotic: 196.43±6.25 (n=14), Lactulose: 203.71±5.33 (n=14), Rifaximin: 198.93±4.43 (n=14) **1 month:** **Stroop's test:** Probiotic: 140.71±6.07 (n=14), Lactulose: 166.07±5.39 (n=14), Rifaximin: 146.86 ± 7.09 (n=14)						

(continued from previous page)

Author, Year [Ref]	HE reversal	Safety and tolerability	Liver function measurements	Quality of life	Effect on gut flora	Serum inflammatory cytokines change	Mortality
Maslennikov R, 2022	**3 months:**<br>**Improvement of HE:**<br>Probiotics: 8/24, Placebo: 1/16	**3 months:**<br>**Adverse events:**<br>**Ascites:** Probiotics: 1/24, Placebo: 3/16	**3 months:**<br>**Child–Pugh class A:** Probiotics: 8/24, Placebo: 1/16				
Mittal V V, 2011	**3 months:**<br>**MHE Reversal:**<br>Probiotics: 14/40, Standard treatment: 4/40	**3 months:**<br>**Serious adverse events:**<br>**Hospitalization:** Probiotics: 1/40, Standard treatment: 2/40<br>**Overt HE:** Probiotic: 2/40, Standard treatment: 4/40<br><br>**Nonadherence:**<br>Probiotics: 3/40, Standard treatment: 3/40					
Pande C, 2012		**6 months:**<br>**Adverse events:**<br>**SBP:** Probiotic: 9/45, Placebo: 8/43<br><br>**Nonadherence:**<br>Probiotics: 10/55, Placebo: 12/55					**6 months:**<br>**Mortality:**<br>Probiotic: 13/45, Placebo: 14/43

(continued from previous page)

Author, Year [Ref]	HE reversal	Safety and tolerability	Liver function measurements	Quality of life	Effect on gut flora	Serum inflammatory cytokines change	Mortality
Pereg D, 2011	**Ammonia:**<br>**Baseline:** Probiotic: 49.6±30.2 (n=18), Placebo: 46.4±23.7 (n=18) (P=0.72)<br>**1 month:** Probiotic: 42.3±23.2 (n=18), Placebo: 47.3±25.3 (n=18)<br>**3 months:** Probiotic: 42.1±26.9 (n=18), Placebo: 45.2±23.6 (n=18)<br>**6 months:** Probiotic: 43.2±22.1 (n=18), Placebo: 48.5±22 (n=18)	**6 months:**<br>**Serious adverse events:**<br>**Hospitalization:** Probiotics: 3/20, Placebo: 3/20<br><br>**Nonadherence:** Probiotics: 2/20, Placebo: 2/20	**Baseline:**<br>**TBIL:** Probiotic: 1.2±0.5 (n=18), Placebo: 1.3±0.6 (n=18)<br>**INR:** Probiotic: 1.1±0.08 (n=18), Placebo: 1.1±0.1 (n=18)<br>**Creatinine:** Probiotic: 1.1±0.4 (n=18), Placebo: 1±0.3 (n=18)<br>**ALT:** Probiotic: 50.2±32.6 (n=18), Placebo: 55±34.5 (n=18)<br>**AST:** Probiotic: 58.4±25.9 (n=18), Placebo: 62.2±32.2 (n=18)<br>**ALB:** Probiotic: 3.6±0.5 (n=18), Placebo: 3.7±0.6 (n=18)<br><br>**1 month:**<br>**TBIL:** Probiotic: 1.2±0.6 (n=18), Placebo: 1.4±0.8 (n=18)<br>**INR:** Probiotic: 1.1±0.1 (n=18), Placebo: 1.1±0.14 (n=18)<br>**Creatinine:** Probiotic: 1.1±0.4 (n=18), Placebo: 1±0.4 (n=18)<br>**ALT:** Probiotic: 49.3±37.6 (n=18), Placebo: 63.7±45 (n=18)<br>**AST:** Probiotic: 56.4±25.4 (n=18), Placebo: 67.2±36 (n=18)<br>**ALB:** Probiotic: 3.75±0.6 (n=18), Placebo: 3.8±0.6 (n=18)<br><br>**3 months:**<br>**TBIL:** Probiotic: 1.1±0.5 (n=18), Placebo: 1.1±0.6 (n=18)<br>**INR:** Probiotic: 1.1±0.09 (n=18), Placebo: 1.1±0.13 (n=18)<br>**Creatinine:** Probiotic: 1.1±0.4 (n=18), Placebo: 0.9±0.2 (n=18)<br>**ALT:** Probiotic: 47.2±32 (n=18), Placebo: 64±30.2 (n=18)<br>**AST:** Probiotic: 54.3±26.3 (n=18), Placebo: 70.4±38.9 (n=18)<br>**ALB:** Probiotic: 3.7±0.6 (n=18), Placebo: 3.8±0.6 (n=18)				

(continued from previous page)

Author, Year [Ref]	HE reversal	Safety and tolerability	Liver function measurements	Quality of life	Effect on gut flora	Serum inflammatory cytokines change	Mortality
Pereg D, 2011			**6 months:**<br>**TBIL:** Probiotic: 1.1±0.4 (n=18), Placebo: 1.1±0.6 (n=18)<br>**INR:** Probiotic: 1.1±0.008 (n=18), Placebo: 1.1±0.1 (n=18)<br>**Creatinine:** Probiotic: 1.1±0.3 (n=18), Placebo: 0.9±0.2 (n=18)<br>**ALT:** Probiotic: 49.6±29.3 (n=18), Placebo: 61.4±31 (n=18)<br>**AST:** Probiotic: 54.4±31 (n=18), Placebo: 66.4±29.4 (n=18)<br>**ALB:** Probiotic: 3.7±0.6 (n=18), Placebo: 3.6±0.5 (n=18)				

(continued from previous page)

Author, Year [Ref]	HE reversal	Safety and tolerability	Liver function measurements	Quality of life	Effect on gut flora	Serum inflammatory cytokines change	Mortality
Pratap Mouli V, 2015	**Baseline:**<br>**NCT-A:** Probiotic: 67.1±15.2 (n=48), Lactulose: 70.8±13.6 (n=51)<br>**NCT-B:** Probiotic: 143.5±38.9 (n=48), Lactulose: 149.6±36.1 (n=51)<br>**FCT-A:** Probiotic: 156.7±20.8 (n=12), Lactulose: 154.3±37.9 (n=9)<br>**FCT-B:** Probiotic: 262.5±35.1 (n=12), Lactulose: 250.2±47.8<br>**P-300 ERP:** Probiotic: 355.6±53.3 (n=60), Lactulose: 349.1±64.4 (n=60)<br><br>**2 months:**<br>**MHE reversal:** Probiotic: 23/33, Lactulose: 25/40<br>**Neuropsychometric test:**<br>**NCT-A:** Probiotic: 52.2±13.4 (n=26), Lactulose: 55.8±14.5 (n=35)<br>**NCT-B:** Probiotic: 114.7±31.6 (n=26), Lactulose: 124.9±32 (n=35)<br>**FCT-A:** Probiotic: 134.3±22.9 (n=7), Lactulose: 123.2±15.4 (n=5)<br>**FCT-B:** Probiotic: 233.9±39.3 (n=7), Lactulose: 217±24.6 (n=5)<br>**Neurophysiological test:**<br>**P-300 ERP:** Probiotic: 322.1±57.3 (n=33), Lactulose: 315.3±57.9 (n=40)	**2 months:**<br>**Adverse events:**<br>**Adverse events:** Probiotics: 3/33, Lactulose: 7/40<br>**Abdominal pain:** Probiotic: 3/33, Placebo: 0/40<br><br>**Serious adverse events:**<br>**Overt HE:** Probiotic: 12/60, Lactulose: 10/60<br><br>**Nonadherence:**<br>Probiotics: 2/60, Lactulose: 2/60					**2 months:**<br>Probiotic: 2/60,<br>Lactulose: 0/60

(continued from previous page)

Author, Year [Ref]	HE reversal	Safety and tolerability	Liver function measurements	Quality of life	Effect on gut flora	Serum inflammatory cytokines change	Mortality
Ramachandran G, 2023		**6 weeks:**<br>**Adverse events:**<br>**Ascites:** Probiotics: 25/93, Placebo: 44/90<br><br>**Serious adverse events:**<br>**Overt HE:** Probiotic: 3/95, Placebo: 9/100<br><br>**Nonadherence:**<br>Probiotics: 2/108, Placebo: 1/107	**Baseline:**<br>**TBIL:** Probiotics: 3.07±0.243 (n=80), Placebo: 3.38±0.394 (n=107)<br>**INR:** Probiotics: 1.40±0.05 (n=80), Placebo: 1.49±0.055 (n=107)<br>**Creatinine:** Probiotics: 1.04±0.048 (n=80), Placebo: 1.04±0.057 (n=107)<br>**ALT:** Probiotics: 32.60±1.67 (n=80), Placebo: 33.85±1.75 (n=107)<br>**AST:** Probiotics: 63.39±4.94 (n=80), Placebo: 66.69±4.23 (n=107)<br>**GGT:** Probiotics: 87.70±9.53 (n=80), Placebo: 68.58±8.65 (n=107)<br>**ALB:** Probiotics: 3.07±0.075 (n=80), Placebo: 3.04±0.084 (n=107)<br>**Serum sodium:** Probiotics: 133.82±0.449 (n=80), Placebo: 132.93±0.443 (n=107)	**Baseline:**<br>**Hand Grip Strength:**<br>Probiotics: 20.66±0.583 (n=108), Placebo: 17.76±0.541 (n=107)<br><br>**6 weeks:**<br>**Hand Grip Strength:**<br>Probiotics: 22.17±0.717 (n=93), Placebo: 18.64±0.569 (n=90)			**6 weeks:**<br>Probiotic: 23/108, Placebo: 33/107

(continued from previous page)

Author, Year [Ref]	HE reversal	Safety and tolerability	Liver function measurements	Quality of life	Effect on gut flora	Serum inflammatory cytokines change	Mortality
Ramachandran G, 2023			**6 weeks:**<br>**MELD score change:** Probiotics: 17.26±0.64 (n=93), Placebo: 17.65±0.649 (n=90)<br>**TBIL:** Probiotics: 2.90±0.264 (n=93), Placebo: 2.86±0.355 (n=90)<br>**INR:** Probiotics: 1.44±0.075 (n=93), Placebo: 1.55±0.097 (n=90)<br>**Creatinine:** Probiotics: 1.05±0.07 (n=93), Placebo: 1.11±0.064 (n=90)<br>**ALT:** Probiotics: 30.96±1.58 (n=93), Placebo: 31.55±1.81 (n=90)<br>**AST:** Probiotics: 58.51±3.74 (n=93), Placebo: 56.75±3.82 (n=90)<br>**GGT:** Probiotics: 80.69±9.16 (n=93), Placebo: 52.65±5.94 (n=90)<br>**ALB:** Probiotics: 3.21±0.87 (n=93), Placebo: 3.07±0.098 (n=90)<br>**Serum sodium:** Probiotics: 133.65±0.534 (n=93), Placebo: 133.34±0.528 (n=90)				

(continued from previous page)

Author, Year [Ref]	HE reversal	Safety and tolerability	Liver function measurements	Quality of life	Effect on gut flora	Serum inflammatory cytokines change	Mortality
Roman E, 2019		**3 months:**<br>**Adverse events:**<br>**Adverse Events:**<br>Probiotics: 10/18, Placebo: 12/18<br>**Ascites:**<br>Probiotics: 0/18, Placebo: 1/18<br><br>**Serious adverse events:**<br>**Infections:**<br>Probiotics: 4/18, Placebo: 5/18<br>**Overt HE:**<br>Probiotics: 1/18, Placebo: 1/18	**Baseline:**<br>**INR:** Probiotics: 1.20±0.06 (n=17), Placebo: 1.18± 0.04 (n=18)<br>**Creatinine:** Probiotics: 74.0±5.2 (n=17), Placebo: 76.1±6.2 (n=18)<br>**ALT:** Probiotics: 30.7±8.1 (n=17), Placebo:36.4±5.1 (n=18)<br>**AST:** Probiotics: 38.6±5.7 (n=17), Placebo: 43.5±4.7 (n=18)<br>**GGT:** Probiotics: 68.4±15.4 (n=17), Placebo: 92.7±16.1 (n=18)<br>**ALB:** Probiotics: 37.9± 0.8 (n=17), Placebo: 36.8±1.4 (n=18)<br>**Serum sodium:** Probiotics: 139.7±0.8 (n=17), Placebo: 138.7±0.8 (n=18)<br><br>**3 months:**<br>**INR:** Probiotics: 1.15±0.05 (n=17), Placebo: 1.19 ± 0.04 (n=18)<br>**Creatinine:** Probiotics: 75.0±6.7 (n=17), Placebo: 75.3±6.6 (n=18)<br>**ALT:** Probiotics: 36.3±9.3 (n=17), Placebo: 41.3±6.5 (n=18)<br>**AST:** Probiotics: 36.9±5.4 (n=17), Placebo: 46.1±4.9 (n=18)<br>**GGT:** Probiotics: 55.2±10.4 (n=17), Placebo: 110.0±22.2 (n=18)<br>**ALB:** Probiotics: 38.1±0.7 (n=17), Placebo: 37.3±1.3 (n=18)<br>**Serum sodium:** Probiotics: 139.9±0.8 (n=17), Placebo: 139.2±0.7 (n=18)	**Baseline:**<br>**Hand grip muscular strength:**<br>Probiotics: 20.76±2.27 (n=17), Placebo: 20.98±2.28 (n=18)<br><br>**3 months:**<br>**Hand grip muscular strength:**<br>Probiotics: 20.62±2.05 (n=17), Placebo: 20.24±2.05 (n=18)			**3 months**<br>Probiotic: 1/18, Placebo: 1/18
Saji S, 2011		**Adverse events:**<br>**1 month:**<br>Probiotics: 0/21, Placebo: 2/22					

(continued from previous page)

Author, Year [Ref]	HE reversal	Safety and tolerability	Liver function measurements	Quality of life	Effect on gut flora	Serum inflammatory cytokines change	Mortality
Sharma K, 2014	**Baseline:**<br>**CFF:** Probiotics: 35.7±4.18 (n=32), Placebo: 37.14±3.07 (n=30)<br><br>**2 months:**<br>**MHE reversal:** Probiotic: 16/32, Placebo: 9/30<br>**Neuropsychometric test:**<br>**CFF:** Probiotics: 38.8±2.78 (n=32), Placebo: 37.99±3.41 (n=30)						**2 months**<br>Probiotic: 1/32, Placebo: 2/30

(continued from previous page)

Author, Year [Ref]	HE reversal	Safety and tolerability	Liver function measurements	Quality of life	Effect on gut flora	Serum inflammatory cytokines change	Mortality
Sharma P, 2008	**Baseline:**<br>**Venous ammonia:** Probiotics: 108.2±37.5 (n=35), Lactulose: 102.3±63.1 (n=35)<br>**Neuropsychometric test:**<br>**NCT-A:** Lactulose: 55.6±8.0 (n=35), Probiotics: 54.5±10.7 (n=35)<br>**NCT-B:** Lactulose: 150.9±22.4 (n=35), Probiotics: 145.5±25.7 (n=35)<br>**FCT-A:** Lactulose: 67.9±19.9 (n=35), Probiotics: 73.0±5.1 (n=35)<br>**FCT-B:** Lactulose: 141.7±36.6 (n=35), Probiotics: 145.6±12.6 (n=35)<br>**Neurophysiological test:**<br>**P300 ERP:** Lactulose: 376.8±22.3 (n=35), Probiotics: 385.4±28.5 (n=35)		**Baseline:**<br>**TBIL:** Probiotics: 1.9±1.1 (n=35), Lactulose: 2.0±1.2 (n=35)<br>**ALT:** Probiotics: 53.6±23.5 (n=35), Lactulose: 42.9±20.9 (n=35)<br>**AST:** Probiotics: 64.3±40.4 (n=35), Lactulose: 57.3±23.4 (n=35)<br>**ALB:** Probiotics: 3.2±0.5 (n=35), Lactulose: 3.1±0.5 (n=35)<br><br>**1 month:**<br>**Child–Pugh class A:** Probiotics: 17/31, Lactulose:18/31<br>**TBIL:** Probiotics: 1.6±0.9 (n=31), Lactulose:1.3±0.6 (n=31)<br>**ALT:** Probiotics: 37.0±11.5 (n=31), Lactulose: 34.3±9.6 (n=31)<br>**AST:** Probiotics: 39.6±20.1 (n=31), Lactulose: 36.8±14.3 (n=31)<br>**ALB:** Probiotics: 3.3±0.5 (n=31), Lactulose: 3.3±0.5 (n=31)				

(continued from previous page)

Author, Year [Ref]	HE reversal	Safety and tolerability	Liver function measurements	Quality of life	Effect on gut flora	Serum inflammatory cytokines change	Mortality
Sharma P, 2008	**1 month:**<br>**MHE reversal:** Probiotics: 14/31, Lactulose: 12/31<br>**Venous ammonia:** Probiotics: 75.7±33.0 (n=31), Lactulose: 69.3±33.3 (n=31)<br>**NCT-A:** Lactulose: 32.0±13.5 (n=31), Probiotics: 38.7±12.6 (n=31)<br>**NCT-B:** Lactulose: 76.7±37.1 (n=31), Probiotics: 95.2±47.6 (n=31)<br>**FCT-A:** Lactulose: 42.6±15.4 (n=31), Probiotics: 39.0±14.6 (n=31)<br>**FCT-B:** Lactulose: 86.7±38.6 (n=31), Probiotics: 74.8±31.1 (n=31)<br>**P300 ERP:** Lactulose: 344.3±30.6 (n=31), Probiotics: 355.5±28.1 (n=31)						

(continued from previous page)

Author, Year [Ref]	HE reversal	Safety and tolerability	Liver function measurements	Quality of life	Effect on gut flora	Serum inflammatory cytokines change	Mortality
Shavakhi A, 2014		**10 weeks:**<br>**Adverse events:**<br>**Abdominal pain:** Synbiotics: 2/19, Control: 1/21<br>**Bloating:** Synbiotics: 4/19, Control: 2/21<br><br>**Serious adverse events:**<br>**Hospitalization:** Synbiotics: 1/19, Control: 1/21<br>**Overt HE:** Synbiotics: 0/19, Control: 1/21<br><br>**Nonadherence:**<br>**6 weeks:**<br>Synbiotics: 1/19, Control: 1/21					

(continued from previous page)

Author, Year [Ref]	HE reversal	Safety and tolerability	Liver function measurements	Quality of life	Effect on gut flora	Serum inflammatory cytokines change	Mortality
Shi J, 2023	**Baseline:**<br>**Ammonia:** Synbiotics: 55.74±7.84 (n=44), Lactulose: 54.63±8.36 (n=44)<br>**Neuropsychometric test:**<br>**NCT-A:** Synbiotics: 108.26±28.69 (n=44), Lactulose: 98.63±34.85 (n=44)<br>**DST:** Synbiotics: 18.71±7.68 (n=44), Lactulose: 16.75±9.58 (n=44)<br><br>**2 weeks:**<br>**Ammonia:** Synbiotics: 36.81±7.56 (n=44), Lactulose: 45.95±5.92 (n=44)<br>**NCT-A:** Synbiotics: 68.45±23.82 (n=44), Lactulose: 86.41±24.28 (n=44)<br>**DST:** Synbiotics: 34.48±8.42 (n=44), Lactulose: 27.45±6.87 (n=44)		**Baseline:**<br>**ALT:** Synbiotics: 88.52±11.06 (n=44), Lactulose: 86.49±12.36 (n=44)<br>**AST:** Synbiotics: 102.54±20.48 (n=44), Lactulose: 97.36±15.74 (n=44)<br><br>**2 weeks:**<br>**ALT:** Synbiotics: 49.25±8.26 (n=44), Lactulose: 64.63±10.29 (n=44)<br>**AST:** Synbiotics: 59.45±18.47 (n=44), Lactulose: 72.63± 11.78 (n=44)			**Baseline:**<br>**IL-6:** Synbiotics: 91.23±9.47 (n=44), Lactulose: 87.26±10.56 (n=44)<br>**Endotoxin:** Synbiotics: 40.80±9.79 (n=44), Lactulose: 42.85±8.46 (n=44)<br><br>**2 weeks:**<br>**IL-6:** Synbiotics: 58.24±8.47 (n=44), Lactulose: 72.48±7.28 (n=44)<br>**Endotoxin:** Synbiotics: 23.12±6.74 (n=44), Lactulose: 31.96±7.60 (n=44)	

(continued from previous page)

Author, Year [Ref]	HE reversal	Safety and tolerability	Liver function measurements	Quality of life	Effect on gut flora	Serum inflammatory cytokines change	Mortality
Xia X, 2018	**3 months:**<br>**Venous ammonia:** Probiotic: 76.4±37.3 (n=30), Control: 152.0±48.36 (n=37)<br>**NCT-A:** Probiotic: 41.2±8.9 (n=30), Control: 72.4±11.5 (n=37)<br>**DST:** Probiotic: 29.8±10.7 (n=30), Control: 12.5±8.3 (n=37)	**3 months:**<br>**Adverse events:**<br>**Ascites:** Probiotic: 8/30, Control: 15/37	**3 months:**<br>**Child–Pugh class B:** Probiotics: 18/30, Control: 15/37<br><br>**TBIL:** Probiotics: 17.6±13.5 (n=30), Control: 48.3±6.7 (n=37)<br>**ALT:** Probiotics: 35.9±22.5 (n=30), Control: 89.1±26.4 (n=37)<br>**AST:** Probiotics: 28.5±18.7 (n=30), Control: 95.6±101.7 (n=37)<br>**ALB:** Probiotics: 62.3±22.5 (n=30), Control: 55.3±25.9 (n=37)<br>**INR:** Probiotics: 1.3±0.4 (n=30), Control: 1.4±0.6 (n=37)				

(continued from previous page)

Author, Year [Ref]	HE reversal	Safety and tolerability	Liver function measurements	Quality of life	Effect on gut flora	Serum inflammatory cytokines change	Mortality
Zhao XH, 2013	**1 month:**<br>**MHE reversal:**<br>Probiotics: 12/40, Placrbo: 4/40<br>**Venous ammonia:**<br>Probiotics: 72.9±15.8 (n=40), Placebo: 76.4±10.2 (n=40)	**Serious adverse events:**<br>**1 month:**<br>**Overt HE:**<br>Probiotics: 2/40, Placrbo: 7/40					

(continued from previous page)

Author, Year [Ref]	HE reversal	Safety and tolerability	Liver function measurements	Quality of life	Effect on gut flora	Serum inflammatory cytokines change	Mortality
Ziada DH, 2013	**Baseline:**<br>**Venous ammonia:** Probiotic: 71.1±19.67 (n=26), Lactulose: 72.29±24.50 (n=24), Placebo: 71.8±15.01 (n=25)<br><br>**1 month:**<br>**Venous ammonia:** Probiotic: 52.47±21.72 (n=26), Lactulose: 55.64±28.10 (n=24), Placebo: 74.54±23.33 (n=25)		**Baseline:**<br>**ALB:** Probiotic: 2.64±0.39 (n=26), Lactulose: 2.73±0.41 (n=24), Placebo: 2.63±0.27 (n=25)<br><br>**1 month:**<br>**ALB:** Probiotic: 2.69±0.44 (n=26), Lactulose: 2.76±0.29 (n=24), Placebo: 2.59±0.33 (n=25)		**Baseline:**<br>**Bifidobacterium:** Probiotic: 7.1±2.37 (n=26), Lactulose: 6.7±2 (n=24), Control: 7.2±2.45 (n=25)<br>**Lactobacillus:** Probiotic: 5.3±1.51 (n=26), Lactulose: 5.9±1.12 (n=24), Control: 5.7±1.73 (n=25)<br>**Enterobacteriaceae:** Probiotic: 9.8±0.81 (n=26), Lactulose: 9.6±0.63 (n=24), Control: 9.4±0.76 (n=25)<br>**Enterococcus:** Probiotic: 8.2±1.24 (n=26), Lactulose: 8.6±1.32 (n=24), Control: 8.2±1.24 (n=25)<br>**Bacteroidaceae:** Probiotic: 9.9±0.51 (n=26), Lactulose: 9.5±0.54 (n=24), Control: 9.9±0.51 (n=25)<br>**1 month:**<br>**Bifidobacterium:** Probiotic: 9.3±0.72 (n=26), Lactulose: 10.8±0.88 (n=24), Control: 6.9±1.89 (n=25)<br>**Lactobacillus:** Probiotic: 9.4±1.23 (n=26), Lactulose: 7.1±1.46 (n=24), Control: 5.8±1.34 (n=25)<br>**Enterobacteriaceae:** Probiotic: 7.4±1.69 (n=26), Lactulose: 7.1±2.21 (n=24), Control: 9.5±0.44 (n=25)<br>**Enterococcus:** Probiotic: 6.3±0.63 (n=26), Lactulose: 7.1±2.45 (n=24), Control: 8.4±1.65 (n=25)<br>**Bacteroidaceae:** Probiotic: 10.5±0.43 (n=26), Lactulose: 10.1±0.88 (n=24), Control: 9.7±0.34 (n=25)		

Table S4. The detailed support for judgement of risk of bias

Study	Bias	Authors' judgement	Support for judgement
Agrawal A, 2012	Random sequence generation	Low risk	Randomization was performed using tables of computergenerated random numbers by an independent person who was unaware of the patient characteristics.
	Allocation concealment	High risk	The study was not blinded.
	Blinding of participants and personnel	High risk	The study was not blinded.
	Blinding of outcome assessment	High risk	The study was not blinded.
	Incomplete outcome data	Low risk	Of 235 patients, 38 (16.2 %) were lost to follow-up, with a median follow-up of 4 months (range 1–7 months), 12 in Gp-L, 13 in Gp-P, and 13 in Gp-N. The compliance with follow-up in our study was 83.8%; 68 patients in Gp-L, 64 patients in Gp-P, and 65 patients in Gp-N were followed up for 12 months. Primary end points were the development of overt HE or a follow-up of 12 months. HE was assessed by West Haven criteria.
	Selective reporting	Low risk	All patients were assessed by means of psychometry test, CFF, and arterial ammonia levels at baseline —arterial ammonia, considered a causative agent for HE and lactulose, was measured again after 3 months of follow-up.
	Other bias	Low risk	Financial support: None.<br>Potential competing interests: None.
Bajaj J S, 2008	Random sequence generation	Low risk	Eligible patients were randomized into the yogurt group and the no-treatment groupnthrough a random number allocation in blocks of five.
	Allocation concealment	High risk	The treatment allocation was concealed from the scorer, but not from the principal investigator.
	Blinding of participants and personnel	High risk	This was a randomized, controlled, single tertiary center trial with open allocation.
	Blinding of outcome assessment	Low risk	This trial masked scoring of end points.

	Incomplete outcome data	Low risk	Seventeen patients were enrolled and three dropped out from the study. Onepatient developed Pseudomonas aeruginosa (P. aeruginosa) septicemia from leg cellulitis after spending time in a public hot tub and died on day 67 without coming for his third visit. Two other yogurt patients did not like the taste and dropped out of the study at days 13 and 17, respectively. Fourteen yogurt-assigned patients completed the study. Eight no-treatment patients were enrolled and two developed OHE evidenced by asterixis: one on day 22 and the other on day 35.
	Selective reporting	Low risk	End points including MHE reversal,OHE development, and adherence were all described in the results at baseline, after 30 days, and after 60 days.
	Other bias	Low risk	Financial support: The General Clinical Research Center at the Medical College of Wisconsin sponsored by the NIH(grant MO1 RR00058) supported this study. Potential competing interests: None.
Bajaj J S, 2014	Random sequence generation	Low risk	Subjects were randomised into placebo or LGG for 4 weeks using blocks of 4 created by the VCU Investigational Pharmacy using a random sequence generator.
	Allocation concealment	Low risk	This is a randomised, placebo-controlled, double-blind trial. Allocation were blinded to the research investigators.
	Blinding of participants and personnel	Low risk	This is a randomised, placebo-controlled, double-blind trial. Treatment were blinded to the participants and personnel.
	Blinding of outcome assessment	Low risk	Monitoring was conducted by an independent Data Safety Monitoring Board (DSMB), and an external NIH/NCCAM Clinical Research Organization (CRO) external monitoring oversight that adhered to FDA protocol for IND regulatory procedures for establishing safety.

	Incomplete outcome data	Low risk	Thirty-seven patients were randomised. Two patients withdrew consent within the first month due to logistic reasons without any adverse events (both LGGgroup). One additional patient had to be scheduled for a splenic arterial embolisation for which he would need antibiotics and narcotics (LGG group) and was withdrawn before receiving medication. Four patients withdrew due to infections or other contraindications to continuation of the study [one broke her wrist and needed antibiotics (placebo), one had an asymptomatic urinary tract infection based on urine collected before randomisation with methicillin-sensitive Staphylococcus aureus (placebo), two were found to have dental issues within a week of randomisation that needed antibiotics (one placebo and one LGG).
	Selective reporting	Low risk	Blood was collected for MELD score, ammonia, serum albumin and pre-albumin, and the dietician met with them to confirm continued adherence on the prescribed diet. If there were no adverse events requiring discontinuation, the subjects were re-prescribed their medication for another 4 weeks. The end-of-drug visit was carried out 4 weeks later (8 weeks after drug initiation) where all procedures including physical examination, cognitive testing,HRQOL evaluation, dietary assessment, sample (blood, urine, stool) collection and evaluation of adherence and adverse events were performed.
	Other bias	Low risk	Declaration of funding interests: JSB received funding from NCCAM, NIH grant U01 AT004428 for this trial. No other personal or funding interests exist. Writing and preparation of this paper was performed by the authors. Declaration of personal interests: None.
Dhiman R K, 2014	Random sequence generation	Low risk	Eligible patients were randomized in an unrestricted 1:1 ratio using a computer-generated random number with central allocation (research staff) and concealment.
	Allocation concealment	Low risk	This is a randomised, placebo-controlled, double-blind trial. Randomization was performed using opaque, sealed envelopes opened by a research staff member at the time of randomization. Allocation were concealed to the research investigators.
	Blinding of participants and personnel	Low risk	The VSL#3 and placebo were identical in terms of color, size, and quantity of each sachet. All patients and personnel were blinded to the randomization sequence.

	Blinding of outcome assessment	Unclear risk	Not mentioned, unable to assess.
	Incomplete outcome data	Low risk	130 patients met the eligibility criteria and were included in the study. 91 patients were excluded from the study, few patients had more than 1 reason for exclusion. Finally, 16 patients in the probiotic group and 14 patients in the placebo group completed the study.
	Selective reporting	Low risk	The primary end point was the development of the first episode of a breakthrough overt HE episode during a follow-up period of 6 months after enrollment. The key secondary end points were the time to first hospitalization for any reason; development of complications, such as overt HE, gastrointestinal hemorrhage, SBP, or sepsis; change in severity of liver disease as measured by CTP and MELD scores from baseline to 4 and 6 months; changes in PHES, SF-36, PSQI, and ESS scores from baseline to 6 months; changes in fasting ammonia, cytokine (IL1, IL6, and TNF-a), plasma renin, plasma aldosterone, andplasma indole levels from baseline to 6 months; and overall survival at 6 months.
	Other bias	Low risk	Funding: This study was supported by CD Pharma India Private Limited (New Delhi, India), who also provided VSL#3 and placebo. The funders did not participate in any part of the study, including the study design, data analysis, or manuscript preparation.<br>Conflicts of interest: The authors disclose no conflicts.
Efremova I, 2024	Random sequence generation	Low risk	Patients included in the study were randomized into test and control groups (ratio 1.5:1). The Excel function RANDBETWEEN (1:5) was used as a random number generator.
	Allocation concealment	High risk	This is a randomized, single-blind, placebo-controlled study. At the end of the treatment period, patients returned the containers with drugs/placebo,and compliance was assessed by counting the remaining capsules. Therefore, the research investigators know the grouping.
	Blinding of participants and personnel	Low risk	The placebo did not differ in appearance from the tested drug. The containers that contained the tested drug and placebo also did not differ from each other in appearance. Patients did not know whether they were taking the probiotic or a placebo.

	Blinding of outcome assessment	Unclear risk	Not mentioned, unable to assess.
	Incomplete outcome data	Low risk	20 patients in the probiotic group and 13 patients in the placebo group completed the study. No patients lost to folllow-up or discontinued intervention.
	Selective reporting	Low risk	The primary outcome is the elimination of SIBO at the end of the 3-month treatmentperiod. Secondary outcomes are changes in the severity of cirrhosis manifestations within the 3-month treatment period and the prognosis for the life of patients within the 2-year follow-up period after the end of this treatment period. All these outcomes were reported.
	Other bias	Low risk	Funding: This study was supported by the Biocodex Microbiota Foundation (National Research Grant Russia 2019).<br>Conflicts of Interest: The sponsor did not participate in the development of the design and did not influence the course of the study, the processing of the results and the decision to publish.
Gupta N, 2013	Random sequence generation	Low risk	Randomization was performed using permuted blocks of 30. The randomization list and numbered packing of the intervention were prepared by a person not involved in this study.
	Allocation concealment	Low risk	Individual randomization codes for each recruited subject were concealed in separate opaque envelopes and marked with the patient number on the outerenvelope.
	Blinding of participants and personnel	Low risk	The individual sealed envelope method was used to maintain blinding of the study participants.
	Blinding of outcome assessment	Low risk	All the envelopes were passed on to the principal investigator (Prof. Sarin) before the study initiation with an instruction that the envelope could be opened only in case of an emergency.
	Incomplete outcome data	Low risk	31 patients were randomized into the V3L#3 group, and 32 in the placebo group. After excluding the discontinued patients, 25 patients in the V3L#3 group and 26 in the placebo group were analyzed for efficacy.

	Selective reporting	Low risk	The primary outcome was response rate (proportion of patients with HVPGreduction from baseline of 20% or to 12 mm Hg). The secondary outcomes were changes from baseline in HVPG, TNF-a, IL-6 and nitrate (NO) levels, and adverse events. All these outcomes were measured at baseline and at the end of 2 months of treatment.
	Other bias	Unclear risk	Financial support: C. D. Pharma, India partly supported this study by providing the study treatments and testing kits. They had no role in conduct of this study or analysis of the results. The investigators have no financial interest in the company or the product. Conflicts of interest: No stated.
Horvath A, 2016	Random sequence generation	Low risk	Eligible patients were randomised in a 1:1 ratio to one of two parallel groups (permutated blocks, Randomizer software, Institute of Medical Informatics, Medical University of Graz).
	Allocation concealment	Low risk	An allocation list was kept by an independent trial pharmacist and disclosed after the last patient has finished the study.
	Blinding of participants and personnel	Low risk	Patients, caregivers, and investigators were blinded to the allocation.
	Blinding of outcome assessment	Low risk	Outcome assessors were blinded to the allocation.
	Incomplete outcome data	Low risk	45 patients were allocated to the probiotic group and 47 revieved placebo. One patients discontinued intervention (consent withdrawn) in the probiotic group, and 11 patients discontinued intervention (consent withdrawn n = 6; death n = 1: liver transplantation n = 2; side effects n = 2).
	Selective reporting	Low risk	The serious adverse events and severity of liver disease measurements were measured at 6 months.

	Other bias	Low risk	Declaration of personal interests: VS has received speaker's honoraria from Institut Allergosan. Declaration of funding interests: This study was funded in full by the Austrian Science Fund (FWF): P24362. AH and MT were supported by the Austrian Science Fund (FWF): P24362, SL by the AustrianScience Fund (FWF): P23532. PhD students AH and SL received funding from the Austrian Science Fund FWF (W1241) and theMedical University Graz through the PhD Program Molecular Fundamentals of Inflammation (DK-MOLIN). TM was supported by the Bavarian Ministry of Sciences, Research and the Arts (BavarianMolecular Biosystems Research Network), the German Research Foundation (Emmy Noether program MA 5703/1-1), the Center for Integrated Protein Science Munich (CIPSM) and the Austrian Science Fund (FWF): P28854. The probiotic and the placebo were provided free of charge by Institut Allergosan, Graz, Austria.
Jayakumar S, 2013	Random sequence generation	Low risk	Randomization was carried out by the hospital pharmacy, using a computer randomization plan obtained from http:///www.randomization.com.
	Allocation concealment	Low risk	The study investigators were blinded to the intervention drug.
	Blinding of participants and personnel	Low risk	The patients were blinded to the intervention drug.
	Blinding of outcome assessment	Unclear risk	Not mentioned, unable to assess.
	Incomplete outcome data	Low risk	Of the 17 patients, two patients were removed from this study prior to completion (one owing to heavy ongoing alcohol abuse, and the second because of an episode of spontaneous bacterial peritonitis and progressive renal dysfunction).Both these patients were on placebo. The data were analysed for the remaining 15 patients (7 on probiotics of VSL#3 and 8 on placebo).
	Selective reporting	Unclear risk	The outcomes were reported at the 2, 4 and 6 week mark. However, the results were shown in a form that could not be included in the synthesis analysis.

	Other bias	Low risk	Financial support: Dr. Tandon received funding from the University of Alberta Hospital Foundation to carry out this research. The authors are also thankful toVSL#3Pharmaceuticals Inc. (Fort Lauderdale, Florida) for supplying both active drug and placebo in kind.<br>Conflict of interest: The authors do not have any disclosures to report.
Koga H, 2013	Random sequence generation	Low risk	Randomization was performed at the Kurume University (H.K.) using computer-generated random numbers.
	Allocation concealment	Low risk	The study investigators were blinded to the interventiong. The LcS (Y400) or lactic acid (placebo) were the same in the two beverages, which were poured into unlabeled paper cups to maintain the ‘‘blindness’’ of the study.
	Blinding of participants and personnel	Low risk	The paticipants and personnel were blinded to the interventiong. The LcS (Y400) or lactic acid (placebo) were the same in the two beverages, which were poured into unlabeled paper cups to maintain the ‘‘blindness’’ of the study.
	Blinding of outcome assessment	Unclear risk	No mentioned, unable to assess.
	Incomplete outcome data	Low risk	The eligible patients for the present study were randomly allocated to two groups, the Y400 group (n = 24) and the placebo group (n = 25). After excluding the insufficient fecal samples, 18 patients in the Y400 group and 19 patients in the placebo group were finally analyzed.
	Selective reporting	Low risk	Serum samples were collected weekly to detect serum rapid-turnover protein levels and conduct liver function test. Conventional liver function tests were performed on the samples. The effects of Y400 on gut flora were evaluated.
	Other bias	Unclear risk	Conflict of interest: The authors have no potential conflicts of interest to disclose.<br>Funding: No stated.
	Random sequence generation	Low risk	One sachet was randomly drawn from this pool for each patient at study entry.
	Allocation concealment	Low risk	The sachets were coded and contents were concealed to the investigators.

Liu Q, 2004	Blinding of participants and personnel	Unclear risk	No mentioned, unable to assess.
	Blinding of outcome assessment	Unclear risk	No mentioned, unable to assess.
	Incomplete outcome data	Low risk	No patients were lost to follow-up at 1 month.
	Selective reporting	Low risk	Quantitative bacteriological analysis of fecal samples, Child-Pugh classification, serum ALT level, venous ammonia levels, and serum endotoxin levels were measured at baseline, and following supplementation for 1 month with the synbiotic preparation.
	Other bias	Unclear risk	Conflict of interest: No stated.<br>Funding: No stated.
Loguercio C, 1987	Random sequence generation	Unclear risk	Participants were randomly assigned to a treatment group. However, no further information about random sequence generation.
	Allocation concealment	Unclear risk	No mentioned, unable to assess.
	Blinding of participants and personnel	Unclear risk	No mentioned, unable to assess.
	Blinding of outcome assessment	Unclear risk	No mentioned, unable to assess.
	Incomplete outcome data	Low risk	All patients completed the treatment period. Five patients given lactulose and four given Enterococcus SF68 did not arrive for post-treatment follow-up. On day 15, two patients given lactulose showed marked hyperammonaemia and a worsening of HE and, therefore, were withdrawn from the study.
	Selective reporting	Unclear risk	The blood ammonia levels, mental status (graded from 0 to 4) before (day 0) andimmediately after treatment (day 10) were measured. However, the results were not shown in detail numbers.
	Other bias	Unclear risk	Conflict of interest: No stated.<br>Funding: No stated.
	Random sequence generation	Low risk	After the basal evaluation, the patients enrolled in the study were assigned to one of two treatments according the Broc Plan computerised randomisationscheme.

Loguercio C, 1995	Allocation concealment	Low risk	The randomisation was provided by the Biometrics Division of Bracco SPA, and was concealed to the investigators.
	Blinding of participants and personnel	Low risk	At the beginning of the study, patients received a box of vials containing capsules of SF68 or a box of bottles filled with lactulose for the entire treatment.
	Blinding of outcome assessment	High risk	Compliance with the treatment was verified by asking relatives to confirm the taking of the drug and by counting the number of bottles or vials left in the box. Therefore, outcome assessors could not be blinded to the allocation.
	Incomplete outcome data	Low risk	Due to worsening of HE or drop-out, at 1 month, there were 18 patients in the SF68 group and 19 patients in the lactulose group for analysis. At 2 months, 15 patients in the SF68 group and 16 in the lactulose group, while 14 patients in the SF68 group and 12 in the lactulose group for analysis.
	Selective reporting	Low risk	Arterial ammonia, reitan's test part A, and mental state of patients were measured after receiving treatment for three periods of 4 weeks, each separated by drug-free 2-week intervals.
	Other bias	Unclear risk	Conflict of interest: No stated.<br>Funding: No stated.
Lunia M K, 2014	Random sequence generation	Low risk	Randomization was performed using tables of computer-generated random numbers.
	Allocation concealment	Low risk	The randomisation was provided by an independent person who was unaware of patient characteristics, and was concealed to the investigators.
	Blinding of participants and personnel	High risk	This was an open-labeled, randomized controlled trial. Allocation was not blinded to the paticipants.
	Blinding of outcome assessment	High risk	This was an open-labeled, randomized controlled trial. Allocation was not blinded to the outcome assessors.
	Incomplete outcome data	Low risk	A total of 160 patients who met the eligibility criteria were included in the study and randomized into 2 groups, group 1 (probiotics) included 86 patients, and group 2 (control) included 74 patients. After excluding the discontinued patients, there were 76 patients in the probiotics group and 62 in the placebo group at 3 months for subsequent analysis.

	Selective reporting	Low risk	Arterial ammonia, CTP score, MELD score, CFF, and PHES were comparable between groups at 3 months.
	Other bias	Unclear risk	Conflicts of interest: The authors disclose no conflicts. Funding: No stated.
Macnaughtan J, 2020	Random sequence generation	Low risk	The randomization list was generated by an independent statistician at the University College London Biomedical Research Unit.
	Allocation concealment	Low risk	The investigators were blinded to the intervention allocation.
	Blinding of participants and personnel	Low risk	The participants and personnel were blinded to the intervention allocation.
	Blinding of outcome assessment	High risk	Compliance was measured by counting returned empty bottles. Therefore, outcome assessors were blinded to the allocation.
	Incomplete outcome data	Low risk	A total of 87 patients who met the eligibility criteria received allocated intervention, and randomized to receive LcS treatment (n=44) of placebo treatment (n=43). After excluding the discontinued patients, there were 33 patients in the LcS group and 35 in the placebo group for subsequent analysis.
	Selective reporting	Low risk	The adverse events and quality of life were reported at the 0-, 1-, and 6-month time-points.
	Other bias	High risk	Funding: The clinical study was funded by Yakult. Conflicts of Interest: Rajiv Jalan has received research funding from Yakult. Jane Macnaughtan has received lecture fees from Yakult. Kaori Suzuki is a current employee of Yakult and Linda Thomas a former employeeof Yakult. All other authors have nothing to disclose. The funders had no role in the collection, analyses or interpretation of data; in the writing of the manuscript; or in the decision to publish the results.
Manzhalii E, 2022	Random sequence generation	Low risk	The 45 participants were randomly assigned at a ratio of 1:1:1 to one of the treatment groups using a computer-generated numeric sequence.
	Allocation concealment	High risk	The investigators were not blinded to the intervention allocation.
	Blinding of participants and personnel	High risk	This was an open-labeled, randomized clinical study. Allocation was not blinded to the paticipants.

	Blinding of outcome assessment	High risk	Patient compliance was evaluated by remnant pill counting and direct questions from an investigator after completion of the treatment.
	Incomplete outcome data	Low risk	After excluding the discontinued patients, there were 14 patients in each group for subsequent analysis.
	Selective reporting	Low risk	The serum ammonia and the Stroop test were reported after 1 month intervention period.
	Other bias	Unclear risk	Funding: No stated.<br>Conflict-of-interest statement: All authors declare no potential conflicting interests related to this paper.
Maslennikov R, 2022	Random sequence generation	Low risk	The Excel function RANDBETWEEN (1:5) was used as a random number generator; for numbers 1 to 3, patients were assigned to the test arm and for numbers 4 or 5, patients were assigned to the placebo group.
	Allocation concealment	High risk	This is a single-blind, randomized placebo-controlled trial, the investigators were aware of the allocation.
	Blinding of participants and personnel	Low risk	Patients were not aware whether they were administered a placebo or the experimental drug.
	Blinding of outcome assessment	High risk	This is a single-blind, randomized placebo-controlled trial, the outcome assessors were aware of the allocation.
	Incomplete outcome data	Low risk	A total of 40 cirrhotic patients were randomly allocated into two groups receiving probiotics (n=24) or placebo (n=16) , nobody withdrew from the planned treatment.
	Selective reporting	Low risk	Outcomes included changes in cardiac output, SVR, the extent of systemic inflammation, severity of ascites and hepatic encephalopathy, serum levels of liver biomarkers, and Child-Pugh scale scores were tested before and 3 months after initiation of probiotics or placebo treatment.
	Other bias	Unclear risk	Funding: Supported by Biocodex Microbiota Foundation: National Research Grant Russia 2019.<br>Conflict-of-interest statement: All authors report no relevant conflicts of interest for this article.
Mittal V V, 2011	Random sequence generation	Low risk	The patients with MHE were randomized using computer-generated random tables by statistician not involved directly with patient care.
	Allocation concealment	Low risk	The sequences were concealed until a decision to enroll a patient was taken after assessment for eligibility and after receiving informed consent.

	Blinding of participants and personnel	High risk	Patients knew which treatment they had received due to the different physical state between the drugs.
	Blinding of outcome assessment	High risk	Compliance was assessed primarily using pill and bottle count, therefore, blinding to the assessor was not possible.
	Incomplete outcome data	Low risk	40 MHE patients were randomly into probiotics group, and 40 in the standard treatment group. Six patients in the probiotics group withdrew from the planned treatment, while 9 patients withdrew from the control group.
	Selective reporting	Unclear risk	The results of neuropsychological tests for presence of MHE, ammonia levels, and health-related quality of life were not well reported at 3 months.
	Other bias	Low risk	Funding: This study is not supported by any financial grants.<br>Conflicts of interest: none declared.
Pande C, 2012	Random sequence generation	Low risk	The randomization sequence was conducted by the statistician.
	Allocation concealment	Low risk	The randomization sequence remained concealed from the investigators until the intervention was assigned.
	Blinding of participants and personnel	Low risk	This is a double-blind placebo-controlled randomized-controlled trial. Patients in group 1 received probiotics capsules at a dose of 2 capsules three times daily, whereas patients in group 2 received placebo capsules at the same doses.
	Blinding of outcome assessment	Low risk	Compliance for intake of medicine was assessed by pill count at each visit. Therefore, blinding to the assessor was not possible.
	Incomplete outcome data	Low risk	55 patients were randomly into probiotics group, and 55 to the placebo group. At 6 months, 22 patients had dropped out from the study (10 in probiotics group and 12 in placebo group).
	Selective reporting	Low risk	The occurrence of SBP within a period of 6 months was considered as the primary endpoint. Side-effects of therapy and mortality were taken as the secondary endpoints. All of these results were recorded at 6 months among all participants.
	Other bias	Unclear risk	Conflicts of interest: There are no conflicts of interest.<br>Funding: No stated.

	Random sequence generation	Low risk	Both probiotic and placebo capsules were delivered in identical pillboxes thatwere consecutively numbered. All the boxes were pooled and one box wasrandomly drawn for each participant at the time of enrollment.
	Allocation concealment	Low risk	Each box contained probiotic bacteria or placebo remained unknown to the investigators until after all patients had completed the study and codes were broken.
	Blinding of participants and personnel	Low risk	Each box contained probiotic bacteria or placebo remained unknown to the participants until after all patients had completed the study and codes were broken.
Pereg D, 2011	Blinding of outcome assessment	High risk	Patient's compliance was monitored by collecting empty boxes at each follow-up visit. Therefore, blinding to the assessor was not possible.
	Incomplete outcome data	Low risk	Forty patients with cirrhosis were enrolled in our study and were randomly assigned to the probiotic (n=20) and the placebo (n=20) groups. Four participants (two from each group) dropped out of the study within the first month post enrollment (all of them due to low compliance), leaving 36 participants eligible for analysis (probiotics, n=18; placebo, n=18).
	Selective reporting	Low risk	Patients enrolled in the study were scheduled for three follow-up visits—after 1, 3, and 6 mo (the end of the study). At enrollment and during all follow-upvisits, blood tests were obtained. A psychometric test and an ammonia level were conducted on enrollment and at the completion of the study.
	Other bias	Unclear risk	Conflicts of interest: No stated.<br>Funding: No stated.
	Random sequence generation	Low risk	Block randomization was used to allocate the patients to lactulose and probiotics groups. The random numbers were generated using Stata software (StataCorp, College Station, TX, USA).
	Allocation concealment	Low risk	Allocation of the patients to receive the study intervention drugs was done by using the sequentially numbered, opaque, sealed envelope method. The envelopes were prepared by a statistician not associated with the conduct of the study, and were opened sequentially only after the patient's name, age and sex were written on them by a person not associated with the study.

Pratap Mouli V, 2015	Blinding of participants and personnel	High risk	This is a non-inferiority randomized controlled open-labeled trial. Allocation could not be blinding to the participants due to the different physical state of drugs.
	Blinding of outcome assessment	High risk	Compliance to intervention drugs was checked by pill count and volume assessment of lactulose, therefore, blinding to the assessor was not possible.
	Incomplete outcome data	Low risk	120 patients were randomized into the lactulose (n=60) or probiotics groups (n=60). Four patients were dropouts and 19 were lost to follow up, two patients died and 22 developed overt encephalopathy, and hence discontinued with the trial drugs due to different management protocols for overt HE. At the end of intervention in 2 months, 40 patients in the lactulose group and 33 patients in the probiotics group were taken for analysis who had completed the study medications.
	Selective reporting	Unclear risk	At the end of treatment in 2 months, 40 patients in the lactulose group and 33 patients in the probiotics group were taken for analysis. However, not all of the patients reported the neuropsychometric test results.
	Other bias	Unclear risk	Conflicts of interest: No stated.<br>Funding: No stated.
Ramachandran G, 2023	Random sequence generation	Low risk	The randomization sequence was computer-generated using block randomization with variable block sizes (https://www.sealedenvelope.com/simple-randomiser/v1/lists). The sequence was generated by a staff member who is not a part of the study.
	Allocation concealment	Low risk	The intervention was distributed to the study participants by a staff member not related to research, based on a 1:1 allocation ratio to receive either probiotic VSL#3 or placebo. Investigators were blinded to randomisation and allocation sequences.
	Blinding of participants and personnel	Low risk	A randomised, double-blind, placebo-controlled study. Both VSL#3 capsules and placebo capsules were similar in colour, size and quantity, and all study participants were given an entire course of treatment in uniform labelled treatment boxes identified by the allocation code. Patients and personnel were blinded to randomisation and allocation sequences.
	Blinding of outcome assessment	Low risk	Treatment boxes were identical in colour, size and shape.

	Incomplete outcome data	Low risk	108 patients were randomized into the probiotics groups, and 107 were randomized into placebo groups. 93 patients in the probiotics group (Lost to follow up, n=6; Expired, n=7; Non-compliance, n=2) and 90 patients in the placebo group completed the trial.
	Selective reporting	Low risk	Adverse events, biochemical parameters, assessment of severity of liver disease (CTP and MELD), and compliance were assessed at baseline and 6 weeks.
	Other bias	Unclear risk	Conflicts of interest: The authors declare that they have no conflict of interest.<br>Funding: No stated.
Roman E, 2019	Random sequence generation	Low risk	Randomization was performed by means of a computer-generated sequence using blocks of four and consecutively numbered opaque sealed envelopes.
	Allocation concealment	Low risk	Patients were randomized by a hepatologist, other than those who selected the patients, to take either a probiotic (probiotic group) or a placebo (placebogroup).
	Blinding of participants and personnel	Low risk	The blinding was maintained for participants and the personnel until the end of the study and the analysis of the results.
	Blinding of outcome assessment	Low risk	The blinding was maintained for the study team until the end of the study and the analysis of the results.
	Incomplete outcome data	Low risk	36 patients were randomized to one of two groups: 18 to the probiotic group and 18 to the placebo group. In the probiotic group, 1 patient died during the treatment period and therefore was not evaluated at the 12-week visit (end of treatment), and gait speed and TUG test at the end of treatment in another patient could not be determine due to hip enthesitis. In the placebo group, 1 patient died after the end of treatment but before the 20-week follow-up visit.
	Selective reporting	Low risk	At the end of treatment in 3 months, adverse events, systemic inflammatoryresponse, mortality, quality of life, and fecal microbiota were assessed.
	Other bias	High risk	Funding: Supported by the Instituto de Salud Carlos III (Madrid, Spain) (PI12/00629), Mendes S.A. (Lugano, Switzerland) and Actial Farmaceutica LDA (Funchal, Portugal).<br>Potential conflict of interest: Dr. Soriano received research and travel grants from Mendes, Actial, and Grifols.

Saji S, 2011	Random sequence generation	Low risk	Randomization was done using random table allocation.
	Allocation concealment	Unclear risk	No mentioned, unable to assess.
	Blinding of participants and personnel	Low risk	The patients were randomized to two groups and the drugs were administered ina double blind fashion.
	Blinding of outcome assessment	Unclear risk	No mentioned, unable to assess.
	Incomplete outcome data	Low risk	There were 3 drop-outs, one in the probiotic group and two in the placebo group.The data reported are only for the intent-to-treat population.
	Selective reporting	Low risk	At the end of 4 weeks patients symptoms were recorded and a thorough examination was done for any features of overt encephalopathy. Investigations were done to reassess the Child's score. Arterial ammonia, number connection test-A and evoked responses were repeated. All of the above outcomes except the Child's score were reported.
	Other bias	Unclear risk	Conflicts of interest: No stated.<br>Funding: No stated.
Sharma K, 2014	Random sequence generation	Low risk	The block randomization method was utilized for random allocation of drugs
	Allocation concealment	Low risk	The sequence remained concealed from the investigator and the generator of the random blocks did not participate in screening, enrolment, or drug delivery.
	Blinding of participants and personnel	High risk	The study was not blinded to the participants and personnel.
	Blinding of outcome assessment	High risk	The study was not blinded to the outcome assessors.
	Incomplete outcome data	Unclear risk	Cirrhotic patients with MHE (n = 124) were randomized into four groups and were given LOLA (n = 31), rifaximin (n = 31), probiotics (n = 32), and placebo (n = 30), respectively, and were followed up fortnightly up to 2 months. A total of 20 patients could not be followed up to the end of the study: 10 lost to follow up, 6 went into overt HE, and 4 expired.

	Selective reporting	Unclear risk	Number of patients who were lost to follow-up and developed overt HE was not well reported.
	Other bias	Low risk	Source of Support: Nil.<br>Conflict of Interest: None declared.
Sharma P, 2008	Random sequence generation	Low risk	Patients were randomized according to a computer-generated randomization chart.
	Allocation concealment	High risk	This is an open-label randomized controlled trial, allocation was not blinded.
	Blinding of participants and personnel	High risk	This is an open-label randomized controlled trial, allocation was not blinded to the paticipants.
	Blinding of outcome assessment	High risk	Compliance with treatment was checked by pill count and volume assessment of lactulose. Due to the different physical state of probiotic pill and lactulose, the allocation could not be blinded to the outcome assessors.
	Incomplete outcome data	Low risk	Equal number of paticipants were randomized into synbiotics, probiotics or lactulose group (n=35). After treatment, there remained 31, 31, and 30 participants in the probiotics, lactulose, and synbiotics group, respectively.
	Selective reporting	Low risk	Outcomes including venous ammonia, biochemical examinations, neuropsychometric test, and neurophysiological test were tested at the end-point at 1 month.
	Other bias	Unclear risk	Conflict of interest: none declared.<br>Funding: No stated.
Shavakhi A, 2014	Random sequence generation	Low risk	Using a table of random numbers generated by random allocation software, patients were randomized into Gp-L and Gp-LPr arms.
	Allocation concealment	Unclear risk	No mentioned, unable to assess.
	Blinding of participants and personnel	Low risk	Participants and dattending physician were blinded to the Gp-L and Gp-LPrarms.
	Blinding of outcome assessment	Low risk	The outcome assessor were blinded to the Gp-L and Gp-LPr arms.

	Incomplete outcome data	Low risk	Eligible paticipants were randomized into Gp-L (n=21) or Gp-LPr (n=19) groups. After excluding the participants lost to follow-up, there remained 20 and 18 participants in the Gp-L and Gp-LPr group, respectively for analyzing.
	Selective reporting	Low risk	The PHES score,development of overt HE, admission in hospital for any other complication of cirrhosis, or death were reported at 10 weeks.
	Other bias	Low risk	Funding: Source of Support: Isfahan University of Medical Sciences.<br>Potential competing interests: None declared.
Shi J, 2023	Random sequence generation	Unclear risk	No mentioned, unable to assess.
	Allocation concealment	Unclear risk	No mentioned, unable to assess.
	Blinding of participants and personnel	Unclear risk	No mentioned, unable to assess.
	Blinding of outcome assessment	Unclear risk	No mentioned, unable to assess.
	Incomplete outcome data	Low risk	A total of 88 patients with MHE were randomly divided into the control (n = 44) and synbiotics groups (n = 44). None of the participants lost to follow-up at the end of the treatment at 2 weeks.
	Selective reporting	Low risk	Ammonia level, biochemical examinations, and liver function tests were collected at 2 weeks.
	Other bias	Low risk	Acknowledgements: None.<br>Conflicts of interest: There are no conflicts of interest.
Xia X, 2018	Random sequence generation	Unclear risk	No mentioned, unable to assess.
	Allocation concealment	Unclear risk	No mentioned, unable to assess.
	Blinding of participants and personnel	Unclear risk	No mentioned, unable to assess.
	Blinding of outcome assessment	Unclear risk	No mentioned, unable to assess.

	Incomplete outcome data	Low risk	Sixty-seven patients were enrolled in this study (probiotic treatment, n=30; no probiotic treatment, n=37). All patients were remained after 3 months of probiotic treatment.
	Selective reporting	Low risk	Venous ammonia level and intestinal mucosal barrier function were tested at 3 months.
	Other bias	Low risk	Declaration of conflicting interest: The authors declare that there is no conflict of interest.<br>Funding: This research received no specific grant from any funding agency in the public, commercial, or not-for-profit sectors.
Zhao XH, 2013	Random sequence generation	Low risk	Randomised table used.
	Allocation concealment	Unclear risk	No mentioned, unable to assess.
	Blinding of participants and personnel	Unclear risk	No mentioned, unable to assess.
	Blinding of outcome assessment	Unclear risk	No mentioned, unable to assess.
	Incomplete outcome data	Unclear risk	No mentioned, unable to assess.
	Selective reporting	Unclear risk	No mentioned, unable to assess.
	Other bias	Unclear risk	Conflict of interest: No stated<br>Funding: No stated.
Ziada DH, 2013	Random sequence generation	Unclear risk	No mentioned, unable to assess.
	Allocation concealment	Unclear risk	No mentioned, unable to assess.
	Blinding of participants and personnel	High risk	This is an open-label randomised controlled trial.
	Blinding of outcome assessment	High risk	This is an open-label randomised controlled trial.

	Incomplete outcome data	Low risk	Group probiotics: 30 allocated to probiotics, 30 received probiotics, 2 lost to follow-up, 1 discontinued therapy, 1 overt encephalopathy,m26 participants analysed.Group placebo: 30 allocated, 0 lost to follow-up, 5 overt encephalopathy, 25 analysed.
	Selective reporting	Unclear risk	No mentioned, unable to assess.
	Other bias	Unclear risk	Conflict of interest: The authors declared that there is no conflict of interest.<br>Funding: No stated.
